# Selective amplification of hypermethylated DNA from diverse tumor types via MSRE-PCR

**DOI:** 10.18632/oncotarget.27825

**Published:** 2020-11-24

**Authors:** Karen B. Chapman, Brandon W. Higgs

**Affiliations:** ^1^Center for Biotechnology Education, Krieger School of Arts and Sciences, Johns Hopkins University, Baltimore, MD 21218, USA; ^2^Genmab, Princeton, NJ 08540, USA

**Keywords:** MSRE-PCR, cDMR, DNA methylation biomarkers, epigenetics, cancer

## Abstract

DNA methylation biomarkers are increasingly utilized for the detection, prognosis and monitoring of cancer. Here we use publicly-available whole genome bisulfite sequencing data to identify differentially methylated regions (cDMRs) in diverse tumor types and further define a set of genomic target regions that have optimal characteristics for Methylation Sensitive Restriction Enzyme-PCR (MSRE-PCR)-based detection: conserved hypermethylation in tumors, abundant MSRE sites and low methylation levels in normal tissues. The identified MSRE-PCR target regions (*n* = 1,294) were primarily encompassed within CpG islands (97%) and promoters (81%) with 39% of the target regions overlapping the transcription start site. Gene set enrichment analysis of the target regions identified significant enrichment of genes involved in neuronal development. A multiplexed MSRE-PCR assay was developed interrogating 47 target regions and was tested on a set of genomic DNAs (*n* = 100) from diverse tumor and normal tissue types including colon, breast, lung, stomach and blood. A logistic regression model containing seven target region amplicons distinguished between tumor and normal tissue in the training (*n* = 50) with a ROC AUC of 0.97 (95% CI [0.92, 1]) and independent test set (*n* = 50) with an AUC of 0.93 (95% CI [0.84, 1]). These findings show that genomic regions with conserved hypermethylation across diverse tumor types, abundant MSRE sites and low methylation levels in normal tissues provide target regions for the detection of tumor DNA via MSRE-PCR. The selective amplification of tumor-derived DNA via MSRE-PCR may have utility in the development of non-invasive cancer detection and surveillance strategies.

## INTRODUCTION

Global epigenetic changes, including DNA methylation, are widely regarded as a hallmark of cancer [1]. Alterations include a decrease in overall CpG methylation levels coupled with discrete regions of hypermethylation, typically localized in the promoter-associated CpG islands. Hypermethylation has been associated with cancer progression [2] and the silencing of growth regulating genes and tumor suppressor genes [3]. As a result, a growing number of DNA methylation biomarkers are being utilized in the development of novel assays for monitoring cancer progression [4, 5], treatment response [6] and early detection [7].

Multiple methods to detect DNA methylation of CpG dinucleotides have been described. Bisulfite treatment of DNA converts unmethylated cytosines to uracil, a property that can be used to detect genome-wide CpG methylation at single nucleotide resolution via Whole Genome Bisulfite Sequencing (WGBS) [8]. PCR methodologies such as Methylation Specific PCR (MSP) also employ bisulfite conversion as well as two sets of PCR primers that distinguish between methylated and unmethylated version of a selected amplicon [4, 9].

Alternatively, without employing bisulfite conversion, selective amplification of differentially methylated regions can be achieved by Methylation Sensitive Restriction Enzyme PCR (MSRE-PCR) [10]. PCR primer-binding sites are designed to flank genomic regions that have a differentially methylated CpG encompassed within a methylation-sensitive restriction site. Methylated DNA is protected from MSRE digestion, thereby allowing selectively amplification. Additionally, MSRE-PCR can be multiplexed to interrogate multiple target regions simultaneously [11] and applications of this technology are being explored for colorectal cancer detection [12] and lung cancer detection [13]. For clinical applications, MSRE-PCR is a desirable strategy in that it avoids bisulfite conversion, a chemical treatment that can result in DNA damage and loss [11]. Preserving the integrity and complexity of the DNA in clinical samples is particularly relevant in the analysis of cfDNA, where sample quantities are low and the fraction of tumor-derived DNA can be limiting.

The selective amplification and detection of tumor-derived DNA via MSRE-PCR requires the targeted amplicon to have MSRE site(s) and to be hypermethylated. In theory, the most optimal amplicons for a highly specific MSRE-PCR assay would be fully unmethylated in normal DNA, and thus a complete digestion would preclude amplification of the normal tissue-derived DNA, facilitating the selective amplification of tumor-derived DNA.

Here we describe a bioinformatics strategy identifying genomic regions that incorporate these criteria to optimize MSRE-PCR assays. We identify amplicons with extremely low methylation levels < 2.5% in normal tissues, conserved hypermethylation in diverse tumors and abundant MSRE sites. Furthermore, we demonstrate the feasibility of using these identified target regions in a multiplexed MSRE-PCR assay in an independent cohort of genomic DNAs from diverse tumors and normal tissues derived from lung, breast, stomach, colon and blood. This strategy may provide a path for sensitive and specific assays for non-invasive cancer diagnostics.

## RESULTS

### Identification of cDMRs

To identify cancer-associated Differentially Methylated Regions (cDMRs), publicly available WGBS datasets from a diverse cohort of normal tissues and tumors were analyzed (Table 1). The normal samples used in this analysis were comprised of 5 different tissue types (liver, lung, breast, colon and blood-derived B-cells) in an effort to simulate the epigenetic diversity in methylation patterns that would be seen in normal DNA. The cancer samples were equally diverse representing lung, liver and colon primary tumors as well as cell lines derived from prostate and breast tumors. In total, 18 WGBS samples representing normal tissues (*n* = 9) and diverse primary tumors and tumor cell lines (*n* = 9) were obtained from the NCBI Sequence Read Archive (SRA) database. In an effort to maximize sequencing coverage and interrogate as much of the genome as possible, multiple runs for individual biological samples were combined to yield an average coverage that exceeded 11-fold. The 48 SRA runs encompassed in this analysis are detailed in Supplementary Table 1.

**Table 1:** WGBS samples used in this study

Sample Type	Name	SRA Sample and Reference	Description	SRA Runs	Mbytes	Reference
**Normal Tissues**
	N3	SRS1351498	normal liver	2	22,600	[36]
	N4	SRS1352201	normal liver	2	12,108	[36]
	N5	SRS1352208	normal lung	1	20,202	[36]
	N6	SRS1353345	normal lung	1	26,014	[36]
	N7	SRS1353348	normal lung	1	12,576	[36]
	N8	SRS1352204	normal liver	1	24,195	[36]
	N9	SRS505928	normal colon	5	27,840	[37]
	N10	SRS505949	normal B-cells	3	36,975	[37]
	N11	SRS505934	normal breast	5	29,531	[37]
**Tumors and Tumor Cell Lines**
	C3	SRS1352199	liver tumor	2	16,983	[36]
	C4	SRS1352202	liver tumor	2	13,510	[36]
	C5	SRS1353344	lung tumor	1	20,783	[36]
	C6	SRS1353347	lung tumor	2	24,795	[36]
	C7	SRS1353349	lung tumor	1	12,894	[36]
	C8	SRS1352206	liver tumor	2	24,173	[36]
	C9	SRS505929	colon tumor	6	36,391	[37]
	C10	SRS505931	prostate cancer cell line	6	38,854	[37]
	C11	SRS505933	breast cancer cell line	5	42,117	[37]

### Global CpG methylation characteristics

**Figure 1:**
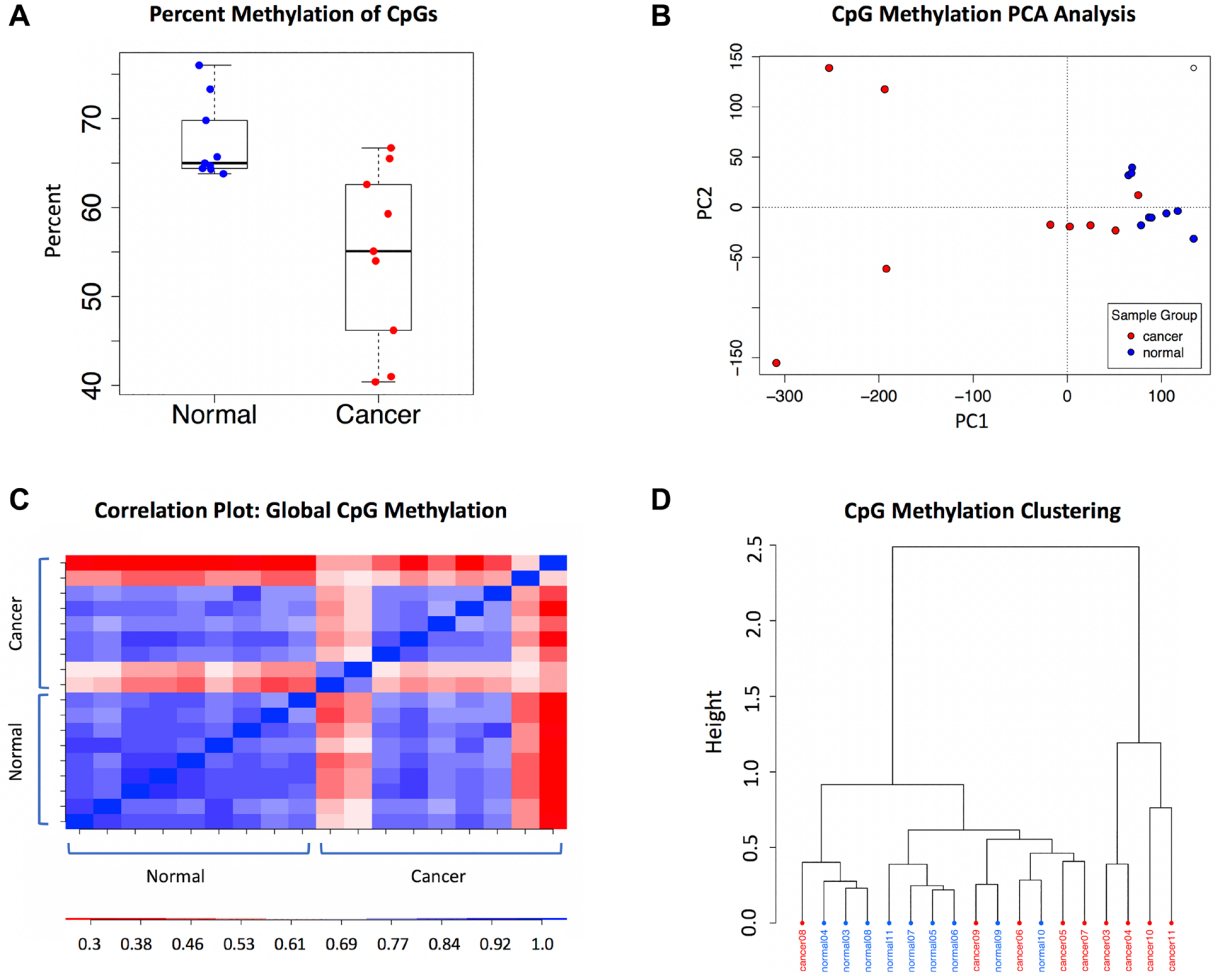
Global methylation characteristics in the WGBS dataset. (**A**) Percent methylation in the sample groups: normal (*n* = 9) and cancer (*n* = 9). (**B**) CpG methylation PCA analysis. Cancer samples are shown in red and normal samples are in blue. (**C**) Pearson correlation plot of global CpG methylation. (**D**) CpG methylation clustering; distance method: correlation, clustering method: ward.

The global genomic CpG methylation levels and patterns in this cohort were characterized to understand the context of differential methylation. Comparing global CpG methylation levels between the cancer and normal samples, the percent methylation in the cancer samples was significantly lower and more variable than the normal sample group (*p* = 0.005). The normal samples had a mean CpG methylation of 67.4%, compared to 54.5% in the cancer samples (Figure 1A). Consistent with the variability observed in the global CpG methylation levels in the cancer samples, principal component analysis (PCA) and Pearson’s correlation analysis also indicated that the cancer samples were more diverse, with the normal samples having an average Pearson’s r = 0.77 versus 0.67 in the cancers (Figure 1B and 1C). Despite the diversity of tissue and tumor types in this WGBS discovery cohort, clustering based on global CpG methylation grouped the majority of the cancer samples together (Figure 1D).

### Identification of cDMRs

**Figure 2:**
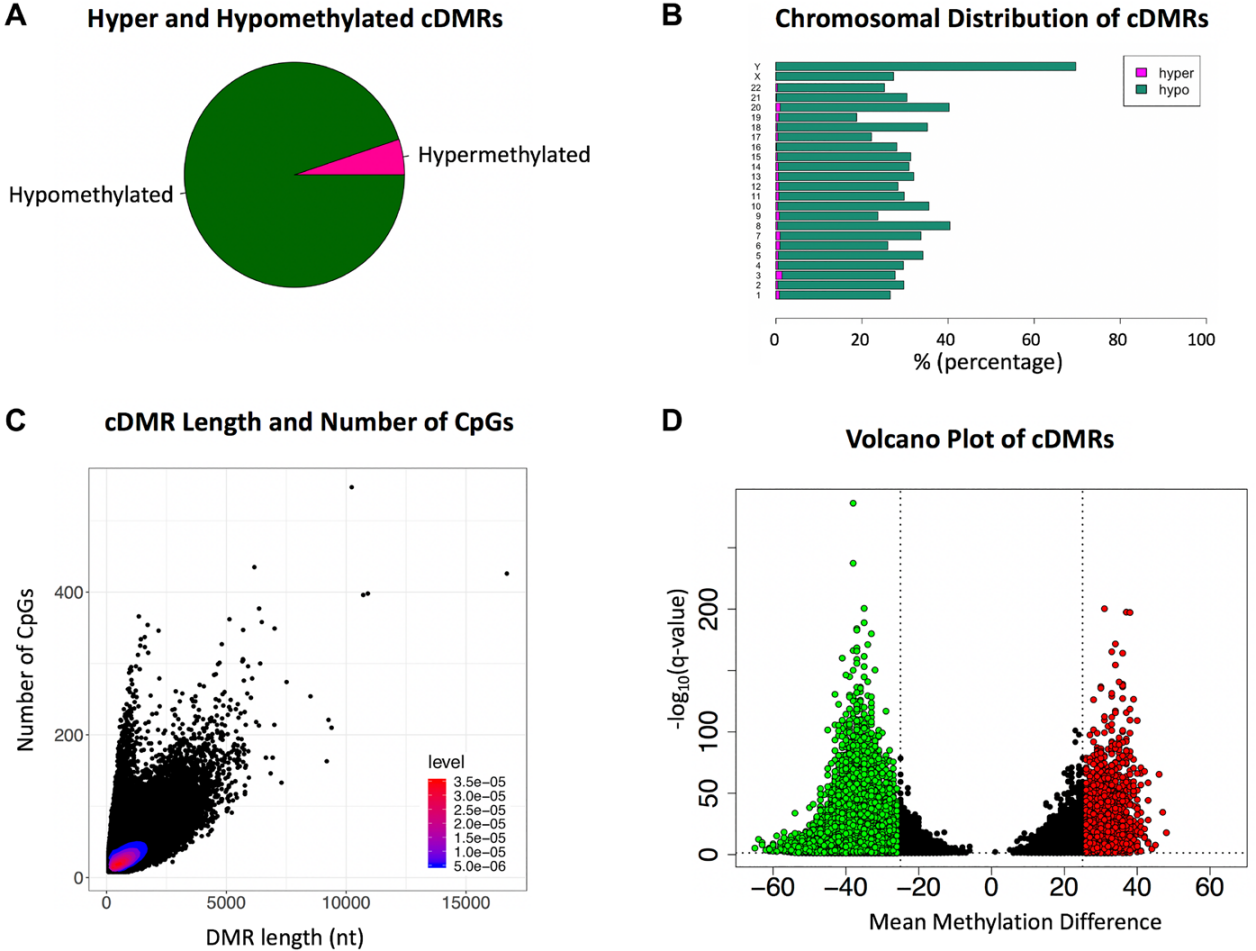
Cancer-associated Differentially Methylated Regions (cDMRs) (**A**) The majority (95%) of the cDMRs are hypomethylated with only 5% hypermethylated in cancer. (**B**) Chromosomal distribution of hypermethylated and hypomethylated cDMRs (**C**) Scatterplot of cDMR length versus number of CpGs, with the density of points indicated via heatmap. (**D**) Volcano plot of the cDMRs showing the mean methylation difference versus the -log_10_ *q*-value. Hypermethylated cDMRs with a *q*-value < 0.05 and mean methylation difference > 25% are indicated in red and hypomethylated cDMRs in green.

Identification of cDMRs was conducted with Metilene [14] using parameters optimized for the identification of regions that would be in a size range suitable for PCR and have multiple differentially methylated CpGs. This analysis yielded 195,590 cDMRs, the majority of which were hypomethylated in cancer (95%), consistent with the lower overall level of CpG methylation observed in the cancer sample group relative to normal (Figure 2A). The genomic location of both the hypermethylated and hypomethylated cDMRs appeared relatively evenly distributed across the chromosomes (Figure 2B). The average size of the identified cDMRs was 885 nt with a mean of 33 CpGs, although some regions exceeded 5 kb with over 200 CpGs (Figure 2C). The more prevalent hypomethylated cDMRs also had a greater maximum methylation difference, with some cDMRs exceeding a mean methylation difference of 60%, compared to a maximum of 48% for the less abundant hypermethylated cDMRs (Figure 2D).

### Identification of target regions for MSRE-PCR

**Figure 3:**
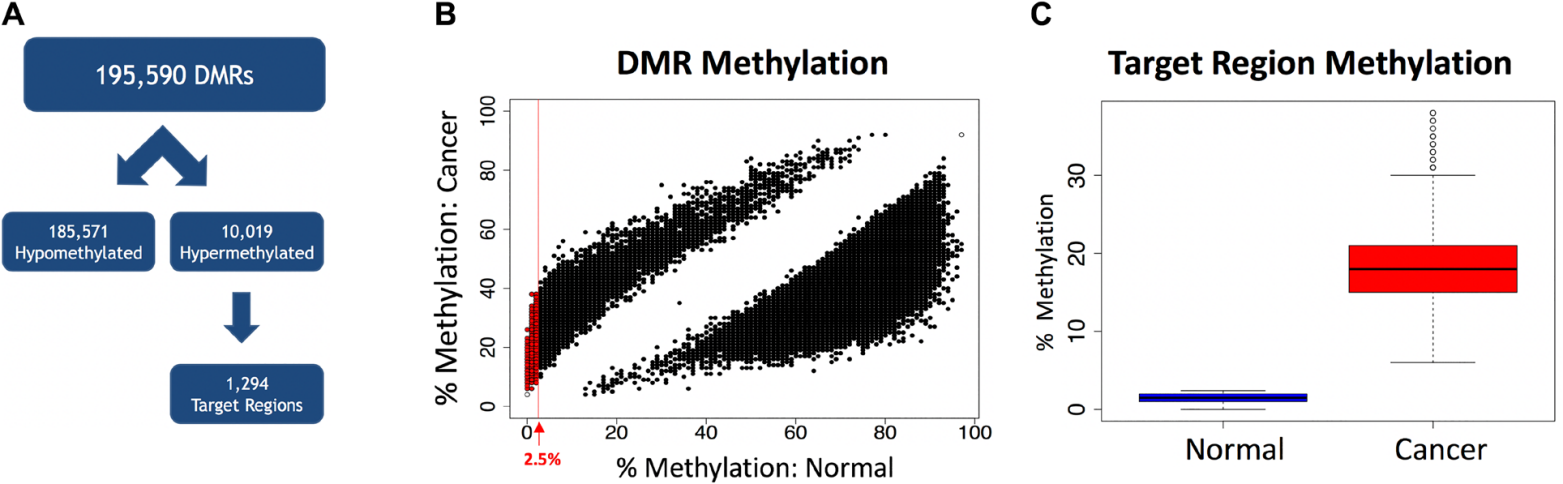
Selection of target regions. (**A**) The 195,590 cDMRs are separated into hypomethylated (*n* = 185,571) and hypermethylated (*n* = 10,019). The hypermethylated cDMRs are filtered for hypermethylated target regions with a mean methylation level < 2.5% in the normal samples, yielding 1,294 target regions. (**B**) A scatterplot of all 195,590 cDMRs showing the percent methylation in normal tissues versus the percent methylation in the cancer samples. The red line shows a mean methylation level = 2.5% in the normal samples. The 1,294 cDMRs passing the filtering criteria (target regions) are shown in red. (**C**) Average methylation levels in the target regions is 1.5% in the normal samples (*n* = 9) and 18.6% in the cancer samples (*n* = 9).

The optimal target region for selective amplification of tumor-derived DNA via MSRE-PCR is a a region methylated in cancer and unmethylated in normal, as this allows for the selective digestion of normal DNA with restriction enzymes that are inhibited by CpG methylation in the recognition site. With these considerations in mind, the 10,019 hypermethylated cDMRs were filtered for those with a mean methylation less than 2.5% in the normal samples, yielding 1,294 target hypermethylated cDMRs (target regions) (Figure 3A and 3B). As a group, the target regions had an average methylation level of 1.5% in the normal samples and 18.6% in the cancers (Figure 3C). The target regions, with an average length of 308 nt and 34 CpGs were smaller and more CpG-dense than the unfiltered cDMRs.

### Target regions had multiple methylation-sensitive restriction sites

**Figure 4:**
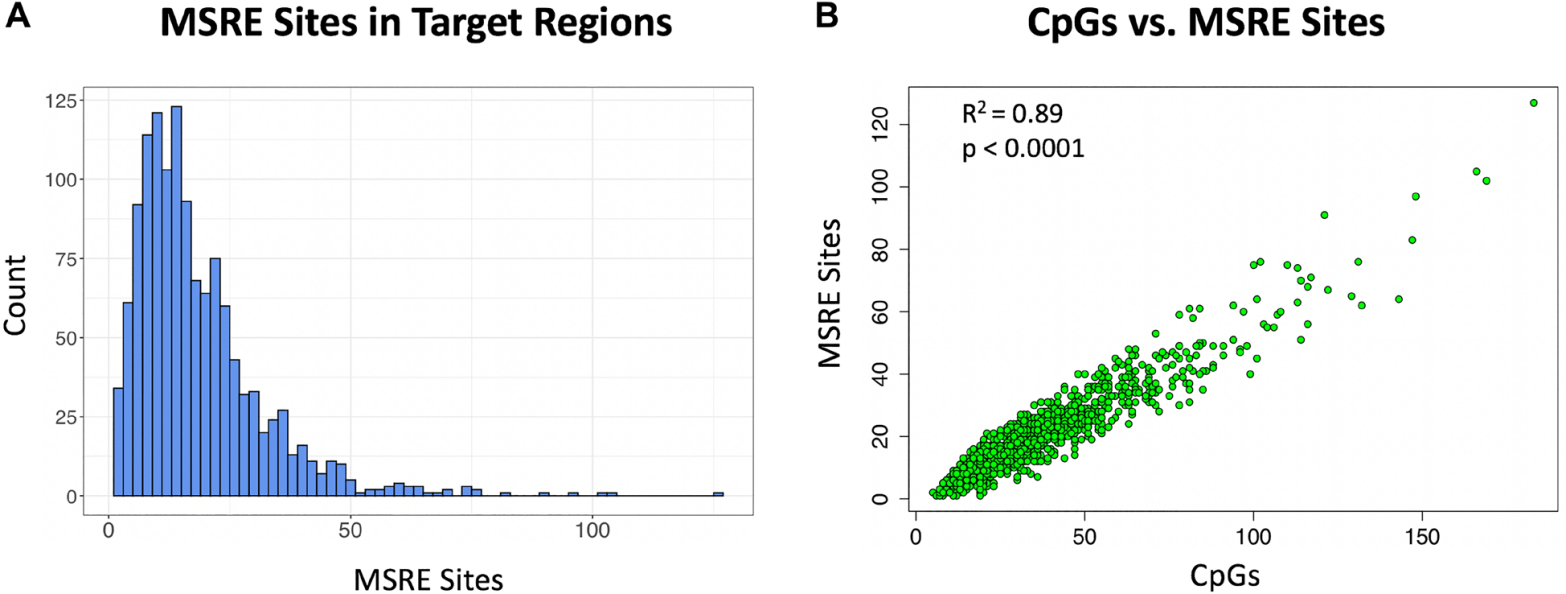
Methylation sensitive restriction sites in target cDMRs. Five MSREs are considered here: HhaI, HpaII, HpyCH4IV, AciI and BstUI. (**A**) Histogram of the number of MSRE sites in the target regions. (**B**) Scatterplot of the number of MSRE sites versus the number of CpGs in the target regions showing a linear relationship (R^2^ = 0.89, *p* < 0.0001).

The selective amplification of target regions via MSRE-PCR requires the presence of MSRE recognition sites that allow for the selective digestion of unmethylated DNA. Here we focused on 5 MSREs: HhaI, HpaII, HpyCH4IV, AciI and BstUI, as each of these enzymes have a 4 base pair (bp) recognition sequence containing CpG and thus sites for these MSREs should be relatively abundant in CpG-rich regions. Indeed, all of the target regions had one or more of these 5 MSRE recognition sites (Figure 4A). The target regions had on average 19 MSRE sites and 34 CpGs and thus more than half of the individual CpGs in these target regions were within recognition sites of one of the five MSREs. The relationship between the number of CpGs in the target regions and the number of MSREs was linear (R^2^ = 0.89, *p* < 0.0001) (Figure 4B).

### Target regions were primarily in promoters and CpG islands

**Figure 5:**
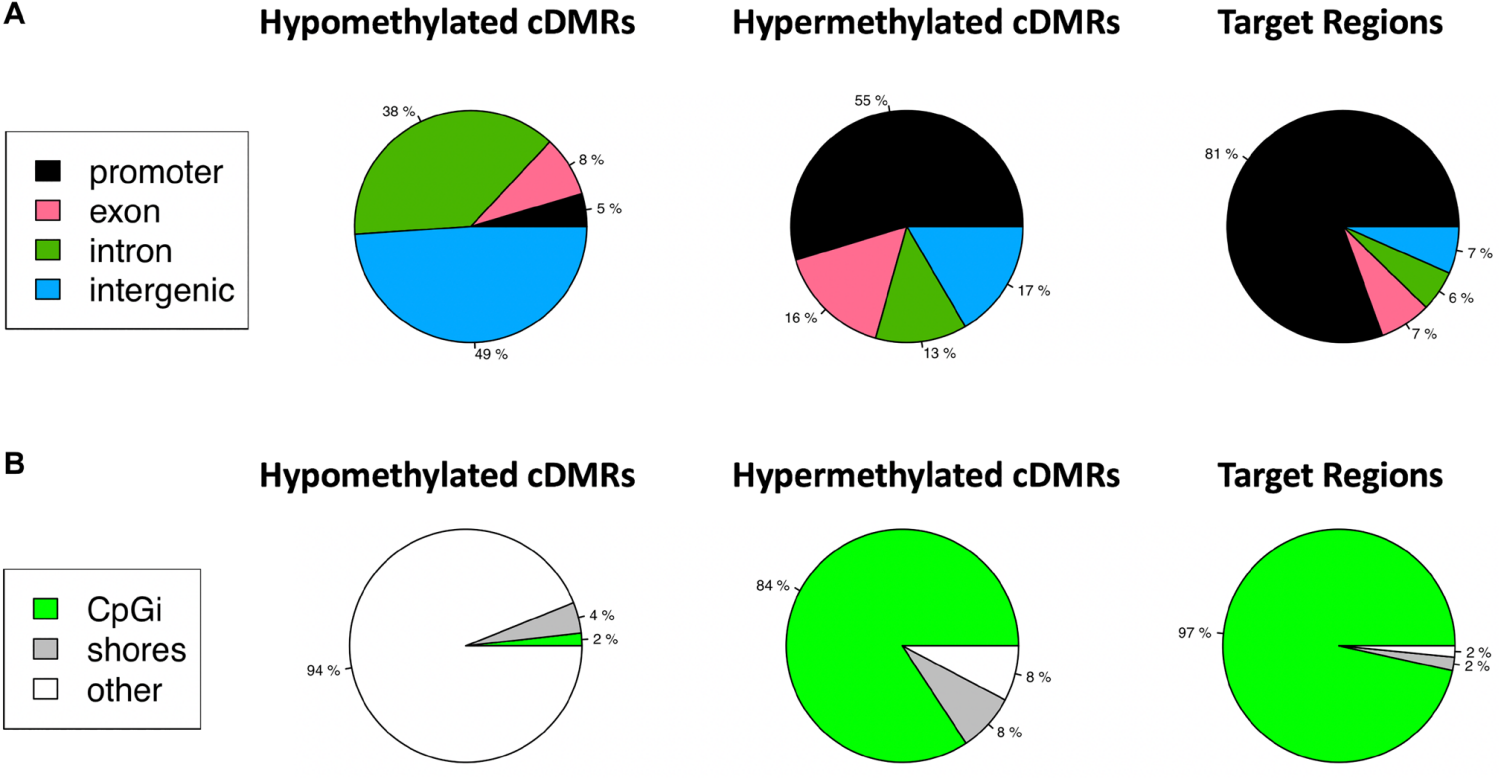
Target regions are enriched for promoter regions and CpG Islands. (**A**) hypomethylated cDMRs, hypermethylated cDMRs, and target regions, a subset of the hypermethylated cDMRs, are annotated with gene parts: promoter, exon, intron or intragenic. 81% of target regions are in promoters. (**B**) cDMRs are annotated with respect to CpG Islands (CpGi), shores or other. 97% of target regions are in a CpGi.

Target regions were not selected based on genomic context or proximity to known genes, rather on optimal amplicon characteristics that would allow for the most selective amplification via MSRE-PCR. These features included size, number of CpGs, low background methylation in normal tissues, and significant methylation across a diverse spectrum of tumor samples. Since the methodology was unbiased with respect to genomic features, we chose to look at the genomic context of the DMRs and selected target regions with respect to known genes, transcripts, CpG islands and shores flanking the CpG islands. The hypomethylated DMRs were located primarily in intergenic regions with only 5% in gene promoters, whereas the hypermethylated DMRs occurred primarily in promoters (Figure 5A). The target regions, which were a subset of the hypermethylated DMRs, showed an even more pronounced enrichment for promoter regions with 81% of the 1,294 target regions located in the promoter. The mean distance to the promoter was 57 nt, with 39% of the target regions overlapping the Transcription Start Site (TSS) (Supplementary Figure 1).

Target regions were located almost exclusively in CpG islands (97%) whereas a lower fraction (84%) of the hypermethylated DMRs were in CpG islands (Figure 5B). This contrasted with the hypomethylated DMRs, 94% of which were located in other regions outside of CpG islands and shores.

### Target regions were enriched for neuronal genes

**Figure 6:**
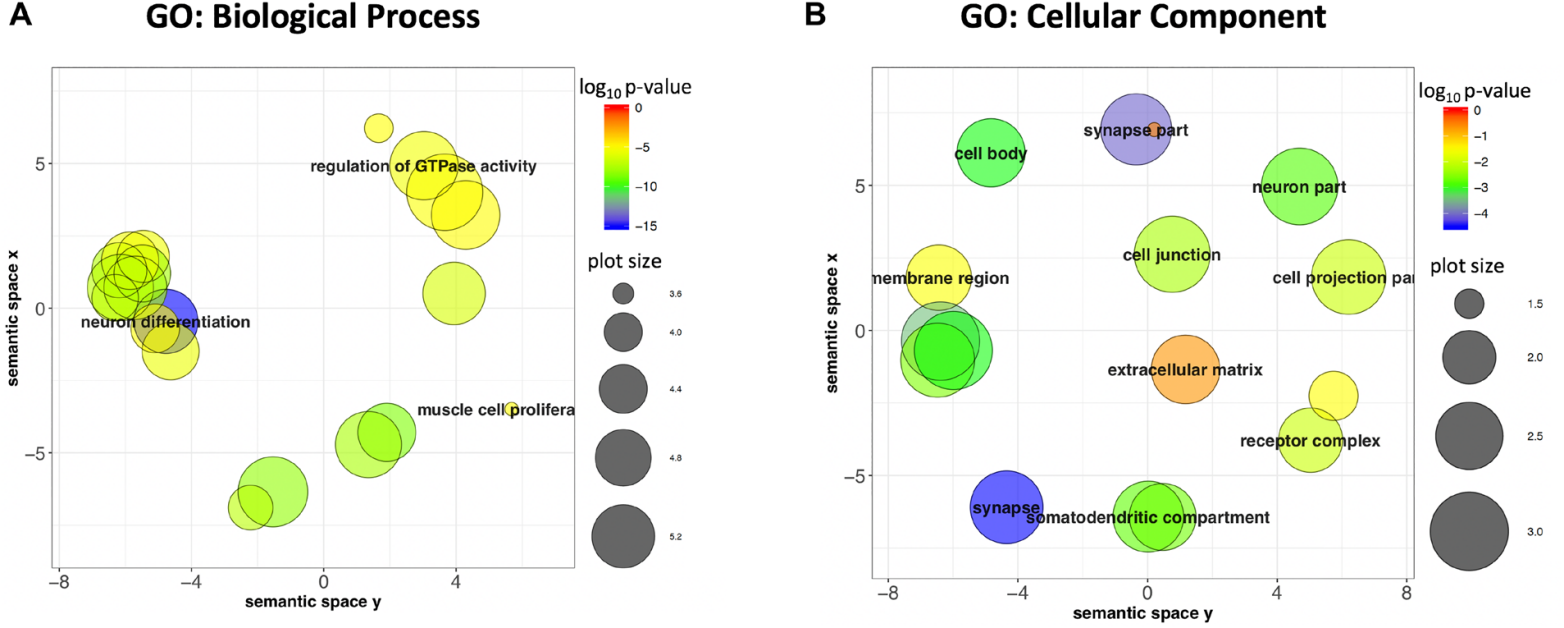
Genes closest to or overlapping target regions are enriched for Neuronal genes. (**A**) GO: Biological Process terms visualized in a semantic similarity-based scatterplot derived in Revigo indicates that neuron differentiation is the most significant term. (**B**) The most significant GO: Cellular Component terms are synapse and synapse part.

Promoter methylation in cancer is largely regarded as being associated with repressive chromatin marks and gene silencing [3]. Since the vast majority of target regions were located in the promoter and 39% overlapped the TSS of known genes, we looked at the function and cellular location of the gene products encoded by the genes closest to the target regions to gain insight into the biological context of the cancer-associated differential methylation observed in these regions. Some of the target regions were adjacent to each other and/or closest to the same gene. Consequently, annotating the 1,293 target regions for the closest genes and then removing redundancies resulted in a reduced list of 562 genes, 39% of which overlapped with the TSS. Functional enrichment analysis using Gene Ontology (GO) molecular function showed significant enrichment for DNA-binding transcription factor activity (FDR-corrected *p*-value 4.3e-11). Analysis of GO:biological processes and GO:cellular components showed concordant enrichment of neuronal function, with significant biological process terms being dominated by neuronal differentiation (FDR-corrected *p*-value 3.9e-14), generation of neurons (FDR-corrected *p*-value 6.1e-14), and the top cellular component terms being glutamatergic synapse (FDR-corrected *p*-value 1.6e-15) and synaptic membrane (FDR-corrected *p*-value 3.1e-13). Since there were a large number of significant GO terms identified, the list of terms was further analyzed in Revigo to summarize clusters of semantically similar GO terms [15]. This analysis also underscored the neuronal theme with the most significant cluster terms being neuronal differentiation for GO:biological process and synapse for GO:cellular component (Figure 6A and 6B).

### Hierarchical clustering

**Figure 7:**
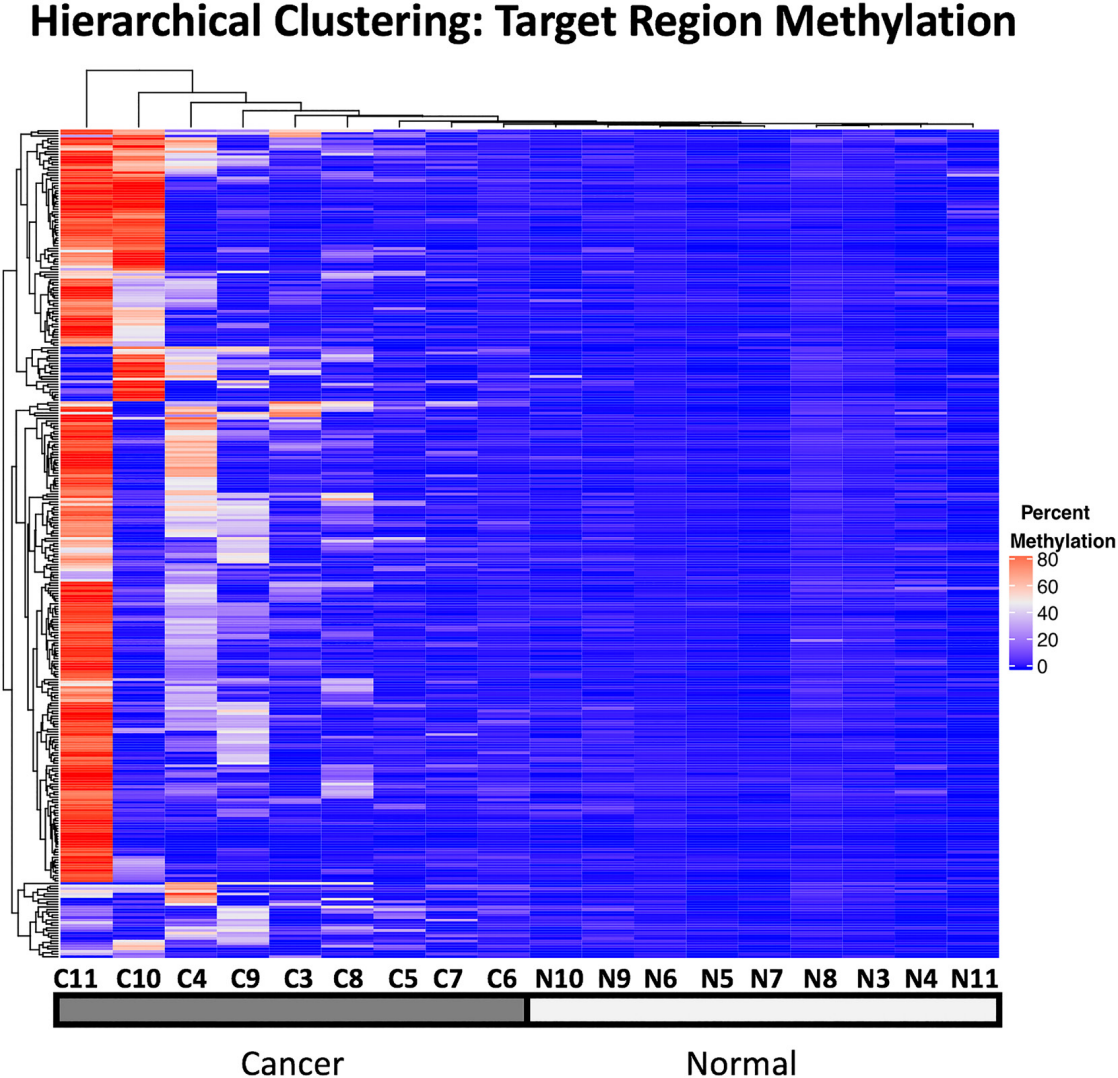
Unsupervised hierarchical clustering of target region methylation in all 18 WGBS samples used in this analysis. Percent methylation is indicated with the color gradient.

Unsupervised hierarchical clustering of the mean methylation in the target regions across the samples shows that hypermethylation of many of these target regions were shared across multiple tumor samples and cancer types, yielding a clustering of the the cancer samples together, distinct from the normal samples which exhibit only low levels of methylation in these regions (Figure 7).

### Multiplexed MSRE-PCR

To test the suitability of the target regions for MSRE-PCR based cancer detection, a multiplexed MSRE-PCR assay was developed and tested on genomic DNAs derived from tumors and normal tissues. The target regions were sorted based on *q*-value and PCR primers for a panel of 47 target amplicons and 3 controls were designed for a multiplexed MSRE assay (Supplementary Table 2). The MSRE assay was carried out by first digesting the DNA samples with 5 MSREs followed by multiplexed end-point PCR with the panel of 50 primer pairs. Quantitation of the individual amplicons was carried out by sequencing the amplification products, quantitating mapped reads and normalizing to an internal control amplicon that was not differentially methylated in the WGBS discovery cohort and thus predicted to amplify consistently across sample types with diverse methylation patterns (Supplementary Table 2).

The performance of the multiplexed MSRE-PCR assay was assessed on an independent, diverse cohort of genomic DNA samples (*n* = 100) encompassing tumor and normal samples from lung, breast, stomach and colon as well as normal peripheral blood leukocytes (Table 2 and Supplementary Table 5). This independent cohort of DNAs is comprised of different samples than were in the WGBS discovery cohort and notably contains several tissue types that were not encompassed in discovery cohort including peripheral blood leukocytes, normal stomach tissue and stomach tumors. The dataset was randomly partitioned into training (*n* = 50) and testing (*n* = 50) sets for predictive modeling.

**Table 2:** Genomic DNA samples used in MSRE-PCR assay

Tissue	Tumors	Normal
Lung	12	8
Breast	12	8
Colon	12	8
Stomach	12	8
Peripheral Blood Leukocytes	0	20

**Figure 8:**
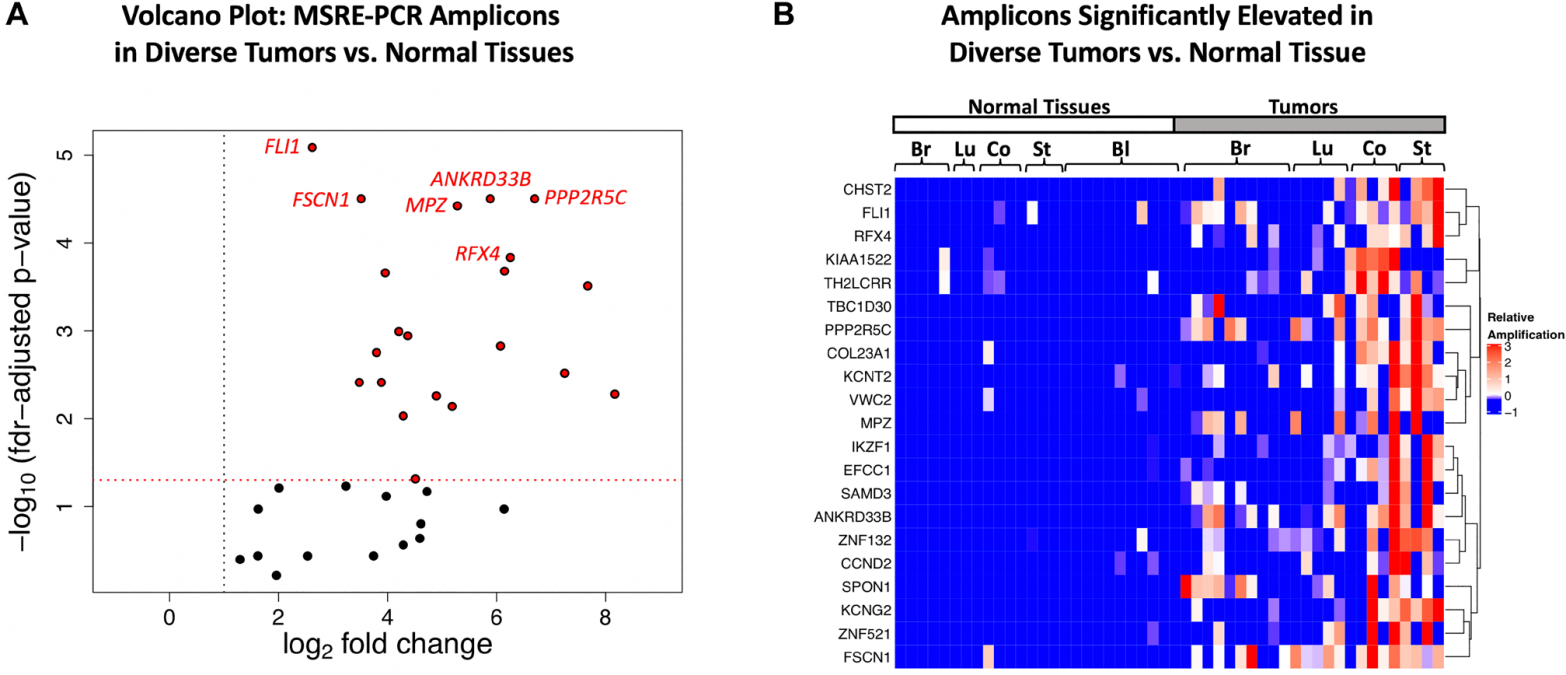
Multiplexed MSRE-PCR of the target regions in genomic DNAs from diverse tumors versus normal tissue. (**A**) Volcano plot of -log_10_ FDR-corrected *p*-values (Mann-Whitney *U* test) versus log2 fold-change in the training set. All 35 amplicons have a linear fold-change > 2 fold elevated in the tumor DNA samples (indicated with the vertical dotted black line). The 21 amplicons with FDR corrected *p*-value < 0.05 (horizontal dotted red line) are indicated with red points. The 6 amplicons with the lowest *p*-values in the training set are labeled with the gene symbol for the overlapping gene or promoter region. (**B**) Hierarchical clustering of the 21 amplicons with FDR corrected *p*-value < 0.05 in the training set with the color indicating the relative level of amplification. The heatmap is supervised in the x-dimension, with samples arranged by tissue type (Br: breast, LU: lung, Co: colon, St: stomach, Bl: blood) and unsupervised in the y-dimension, with amplicons labeled by the gene symbol of the overlapping gene or promoter region.

The performance of the multiplexed MSRE-PCR assay in the training set was assessed by comparing the relative levels of amplification in the two sample groups. Amplicons with fewer than 10 reads per sample on average were removed from the analysis (*n* = 12) leaving 35 amplicons. As shown in the volcano plot in Figure 8A, all 35 of the amplicons had > 2-fold elevated amplification in the tumor DNA samples relative to the normal tissue DNA samples, with 27 exhibiting > 10-fold. Most of the amplicons (21/35) of exhibited significant elevation in tumor samples (FDR corrected *p*-value < 0.05, Mann-Whitney *U* Test). Visualization of these 21 amplicons via hierarchical clustering shows that most of the tumor DNA samples have elevated amplification levels of multiple amplicons relative to the normal tissue DNAs (Figure 8B). Additionally, none of the amplicons are specific for a single tumor type; most are broadly elevated in a subset of samples across the four tumor types surveyed here: breast, lung, colon and stomach tumors. The Receiver Operating Characteristic (ROC) area under the curve (AUC) for individual amplicons in the training set ranged from 0.54 to 0.89, with 9 amplicons having an AUC > 0.8 in the training set (Supplementary Table 6).

### MSRE-PCR panel modeling and prediction of cancer status

**Figure 9:**
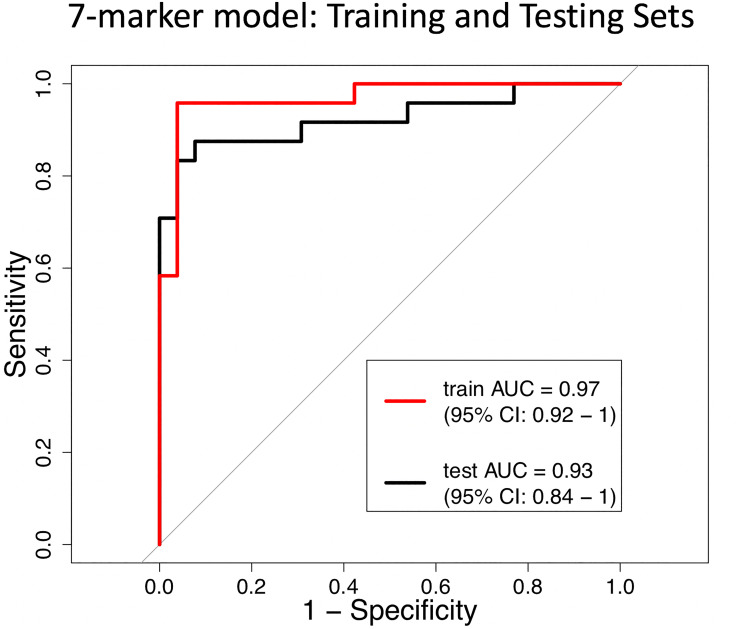
Receiver operating characteristic (ROC) curves of the 7-marker logistic regression model in distinguishing between tumor and normal tissue DNA. The performance of the model in the training set (*n* = 50) is shown in red (AUC = 0.97, 95% CI: 0.92–1) and the independent test set is shown in black (AUC = 0.93, 95% CI: 0.84–1).

The MSRE-PCR training set (*n* = 50) was used to develop a predictive model and also explore the minimum number of amplicons that are required to accurately classify sample DNAs as either tumor or normal tissue. The R package glmnet [16] was used to develop a logistic regression model using a lasso penalty for feature selection. This analysis yielded a 7-marker model that performed with a ROC AUC in the training set of 0.97 (95% CI [0.92, 1]). The performance of this 7-marker model was validated in the independent test set (*n* = 50), yielding an AUC of 0.93 (95% CI [0.84, 1]) (Figure 9). The 7 markers used in this model correspond to amplicons that are contained within the gene or upstream promoter regions of *ANKRD33B*, *CHST2*, *SPON1*, *PPP2R5C*, *KCNG2*, *KIAA1522* and *TH2LCRR*.

**Figure 10:**
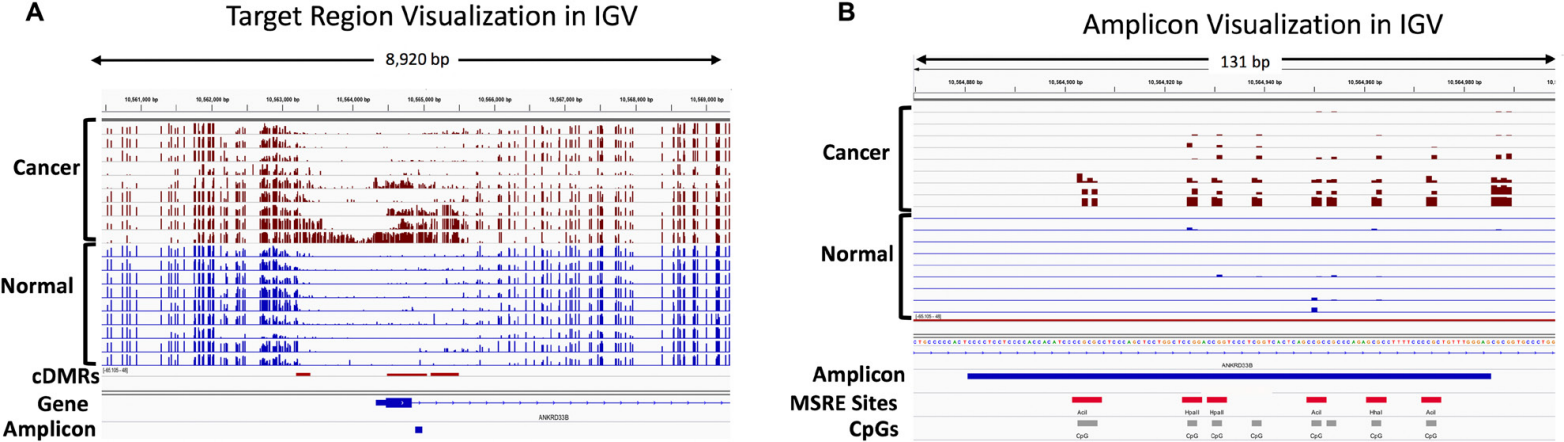
IGV visualization of the WGBS discovery cohort showing hypermethylated cDMRs and the location of an amplicon in the *ANKRD33B* gene. Percent methylation for individual CpGs is indicated with bar graphs for each of the cancer samples (maroon) and normal tissue samples (blue) used in the WGBS data analysis. (**A**) The location of 3 hypermethylated cDMRs in the promoter and gene body of *ANKRD33B* is indicated with the red rectangles in the cDMR track and the location of the MSRE-PCR amplicon is shown in blue in the Amplicon track. (**B**) A zoomed-in view of the MSRE-PCR amplicon shows the position of the individual CpGs (grey) and MSRE sites (red) within this 106 bp amplicon.

Visualization of a representative target region in the *ANKRD33B* gene is shown in Figure 10A. Three identified hypermethylated cDMRs are shown, with the WGBS tracks illustrating the promoter hypermethylation in the cancer samples and the relatively low levels of methylation in the normal tissues in the discovery cohort. Figure 10B shows a close-up of the 106-bp MSRE-PCR amplicon within a target region in the *ANKRD33B* gene containing 9 CpGs, 7 of which overlap with an MSRE site.

## DISCUSSION

Here we describe the identification of cDMRs in diverse tumor types using publicly available WGBS data and further define a set of 1,294 target regions with optimal characteristics for MSRE-PCR-based detection of tumor DNA. The utility of these target regions in the context of MSRE-PCR is shown here with a multiplexed assay demonstrating that all 35 amplicons were > 2-fold elevated in tumor-derived DNA relative to normal tissue DNA. In a diverse cohort of tumor and normal tissue DNAs, most of the individual MSRE-amplicons (21/35) showed significantly elevated amplification in tumor-derived DNA versus normal (FDR-corrected *p* < 0.05). Furthermore, despite the diversity of tumor and tissue types in the MSRE-PCR cohort, a logistic regression model using 7 of the amplicons distinguished between tumor versus normal tissue with an AUC of 0.97 in the training set and 0.93 in the independent test set. This result underscores the conservation of hypermethylation in these regions across diverse tumor types and also demonstrates the utility of using MSRE-PCR to detect tumor-derived DNA.

In the analysis of the WGBS discovery cohort, we observed that overall CpG methylation levels were lower and more variable in the tumors and this global loss of methylation was punctuated by local regions of hypermethylation, an observation consistent with multiple reports in the literature [2, 17]. We further defined target regions for MSRE-PCR, a subset of the hypermethylated cDMRs that had low background methylation in normal tissues. Although the WGBS methodology used to identify targets was genome-wide with an average of 11-fold coverage and hence relatively unbiased with respect to genomic features, we observed significant enrichment of target regions in gene regulatory regions with 81% in promoters and 97% in CpG islands. Furthermore, these target regions were very proximal to, or overlapping the TSS with a mean distance of 57nt and 39% overlapping the TSS. This was in stark contrast to the hypomethylated DMRs with only 5% in promoters and only 2% in CpG islands, perhaps reflecting the global loss of CpG methylation that is observed in these tumor samples.

A recent study that explored the correlation of methylation, transcription and chromatin structure found that in normal tissues, methylation 1 kb upstream of the TSS was most correlated with gene silencing, but in cancer, there was a shift to the TSS [18]. Given that the target regions described here were primarily in the promoter region and overlapping or close to the TSS, it is reasonable to hypothesize that these genes are likely being silenced in cancer.

Despite the diversity of normal tissue types in the WGBS discovery cohort, the MSRE-PCR target regions identified here were nearly universally unmethylated, with an average methylation within these CpG dense region of only 1.5%. The very low levels of methylation in these regions in normal tissues suggest that there are stringent and vigilant mechanisms in place to keep these regions clear of methylation marks. The hypermethylation of these target regions in cancer thus represents a departure from a precisely maintained unmethylated state and indicates a change that is generally conserved to some extent across the diverse tumor types used in this study. This evidence suggests that the genes associated with these target regions were acquiring epigenetic marks in cancer that are likely silencing these genes - changes that may well be relevant to oncogenic transformation and/or disease progression.

The set of genes closest to or overlapping the target regions showed a significant enrichment in neuronal development and synapse function which underscore a neuronal theme to this set of target genes. Interestingly, it has been reported that the neurofilament genes *NEFH*, *NEFL* and *NEFM*, gene products that comprise the major cytoskeletal component of neurons, are frequently silenced via promoter methylation in a variety of cancers including breast, pancreas, gastric and colon and this gene inactivation is correlated with disease progression and adverse clinical parameters [19]. Reintroducing NEFH expression in breast cancer cells reduced invasiveness, suggesting that expression of neuronal filaments in cancer induced a rearrangement of the cytoskeleton that reduced motility and migration. Another study showed that inhibition of epigenetic modification enzymes, including DNA methyl transferases (DNMTs), induced a post-mitotic, neuronal-like terminal differentiation in different types of cancer cells [20]. These studies, taken together with the targeted promoter methylation of neuronal-related genes shown here, underscore an emerging pattern of tumor-associated silencing of neuronal genes, a mechanism which may facilitate the maintenance of an undifferentiated, proliferative state with the oncogenic characteristics of mobility and invasiveness.

The target regions identified here are not only differentially methylated in tumors versus normal tissues, but also have the unique characteristic of very low average methylation levels < 2.5% in normal tissues. In the context of MSRE-PCR, this hypomethylation status in normal DNA in theory allows for the nearly complete digestion of normal DNA, as only methylated MSRE sites will be protected from digestion. Furthermore, in addition to an extremely low levels of methylation in normal tissues, each amplicon contains multiple CpGs that are interrogated by one of the MSREs. For successful amplification, all of the MSRE sites on a single molecule need to be methylated and protected from digestion for amplification of that molecule to proceed. Thus, the hypomethylation in normal tissues taken together with multiple MSRE sites within these regions should facilitate the digestion of the normal DNA relative to tumor-derived DNA and consequently enhance the specificity of the assay. Indeed, here we observed that all 35 amplicons tested in the multiplexed MSRE-PCR assay exhibited > 2-fold elevated amplification in the tumor samples, with the majority of the amplicons (27 of 35) exhibiting > 10-fold elevation.

The multiplexed MSRE-PCR feasibility study on diverse tumor and normal tissue genomic DNAs reported here identified 21 genes with significantly elevated amplification in this assay and further defined a 7-marker logistic regression model that distinguished between tumor and normal tissue with an AUC of 0.94 in the independent test set. The 7 markers used in this model correspond to amplicons contained within the gene or promoter region of *ANKRD33B*, *CHST2*, *SPON1*, *PPP2R5C*, *KCNG2*, *KIAA1522* and *TH2LCRR*. Consistent with the neuronal theme identified in the gene set enrichment analysis, *SPON1* is a cell-surface adhesion molecule involved in sensory neuron attachment; its expression has been associated with metastatic progression in osteosarcomas [21]. Both *PPP2R5C* and *KIAA1522* have also been associated with cancer progression and/or prognosis. *PPP2R5C* encodes an isoform of the B regulatory subunit of protein phosphatase 2A, a serine/threonine phosphatase widely implicated in the regulation of mitogenic pathways [22]. Aberrant expression of *KIAA1522* has been observed in non-small cell lung cancer and is associated with poor outcome [23]. *ANKRD33B*, encoding ankyrin repeat domain 33B, has been shown to be differentially methylated in fetal lung in association with nicotine exposure in utero [24].

The multiplexed MSRE-PCR assay described here interrogated 35 target regions using endpoint PCR and quantitated amplification via sequencing. While this strategy is convenient to assess many amplicons simultaneously, the relatively small number of features required for sample classification enables the development of a qPCR-based assay that interrogates fewer target regions in individual qPCR reactions or as a multiplexed reaction with fluorescent reporter probes. In the multiplexed MSRE-PCR assay described here, there is some variability in the performance of the individual amplicons, with some showing only modest levels of elevated amplification in tumor DNA samples. The relatively poor performance of some amplicons may be attributable to the challenges of moving from the WGBS platform to a PCR platform along with variable amplification efficiencies in these GC-rich regions, particularly in the context of a highly multiplexed amplification reaction. While the multiplexed endpoint MSRE-PCR assay showed that 27 of the 35 target regions had > 10-fold elevated amplification in the tumor DNA samples, this fold-change may well be enhanced by utilizing a qPCR strategy that quantitates amplification in real time. The robust elevated amplification of tumor DNA observed here in genomic DNA samples lays the foundation for applying multiplexed MSRE-PCR strategies for the detection of tumor DNA in more complex sample types such as cfDNA in blood, where the fraction of tumor-derived DNA may be limiting.

While this study included multiple normal tissue and tumor types, it is nevertheless limited in the scope of tumor types examined, with the cohort for the MSRE-PCR assay encompassing breast, stomach, lung, and colon tumors. While this cohort is diverse, it is by no means comprehensive and thus the utility of the MSRE-PCR assay defined here and the conservation of the cDMRs in a large and more comprehensive cohort remains to be investigated.

The data presented here define genomic target regions for MSRE-PCR detection of tumor DNA and establish the utility of these target regions in a multiplexed MSRE-PCR assay. MSRE-PCR enables the selective amplification of hypermethylated tumor-derived DNA while avoiding harsh bisulfite treatment and thus is a strategy ideally suited for the challenges of non-invasive detection. The hypermethylated MSRE-PCR target regions defined here may be useful in the development of non-invasive tests for tumor detection and/or monitoring.

## MATERIALS AND METHODS

### WGBS data analysis

WGBS raw data was obtained from the NCBI Sequence Read Archive (SRA) database [25]. 48 individual SRA runs representing 18 biological samples were analyzed (Table 1 and Supplementary Table 1). Multiple runs for the same biological sample were combined to optimize coverage. The SRA Toolkit [25] was used to extract fasta files and fastQC [26] was used to asses the quality of the raw data. Poor quality sequences and adapters were trimmed with Trimmomatic [27] and the quality of the trimmed reads was assessed with fastQC. The bisulfite converted reads were mapped to hg38 and CpG methylation calls were extracted using Bismark [28]. Differentially methylated Regions (DMRs) were identified with Metilene [14], filtering the output for minimum mean methylation difference of 0.15, minimum size of 80 nucleotides and *Q*-value < 0.01. Summary figures and annotation of DMRs was performed in R using the package methylKit [29]. Target regions were defined as hypermethylated DMRs with a mean methylation < 2.5% in normal samples.

### MSRE-PCR

PCR primers were designed using Benchling [30] with the considerations that the amplicons have at least 2 MSRE sites, an amplicon size < 150 bp and PCR primer melting temperatures of approximately 61 degrees (Supplementary Table 3). Descriptive characteristics of the 47 target regions and 3 controls in the multiplexed MSRE-PCR panel such as the genomic coordinates and the number of CpGs and MSRE sites within each amplicon is detailed in Supplementary Table 2. Genomic DNA samples from lung, breast, stomach and colon tumors and normal tissues were obtained from Biochain (Newark, CA, catalog numbers D8235152-1, D8235086-1, D8235090-1, D8234148-1, D8235248-1) (Supplementary Table 5). Oligonucleotides were synthesized at IDT (Coralville, Iowa). DNA samples (20 ng) were digested with 5 Units each of 5 MSREs: HhaI, HpaII, HpyCH4IV, AciI and BstUI, all from New England Biolabs (Ipswich, MA).

The amplification of the multiplexed panel was done in several steps, incorporating a hybrid selection step to enrich for target sequences and minimize off-target amplification. Briefly, 5 ng of digested genomic DNA was subjected to 10 cycles (94°C 3 min [95°C 30 s, 61°C 30 s, 72°C 30 s] × 10) of multiplexed PCR amplification using the 50 MSRE-PCR primer pairs and Platinum II Hot-Start Green PCR Master Mix (ThermoFisher Scientific). The preamplification reaction was hybridized to a pool of 50 biotinylated oligonucleotides (custom IDT xGen Lockdown probe pool, Supplementary Table 4) complimentary to internal sequences within each amplicon. The xGen Hybridization and wash kit (IDT, Coralville, Iowa) was used for target enrichment according to the manufacturer’s protocol. After hybrid selection, the magnetic beads containing the captured target amplicons were resuspended in 10 uL of dH_2_O and 5 uL was used in a 20 uL amplification reaction with the 50 multiplexed MSRE primer pairs for 35 cycles (94°C 3 min [95°C 30 s, 61°C 30 s, 72°C 30 s] × 35).

Amplification products were sequenced on the Illumina platform at Genewiz (South Plainfield, NJ, USA) at a depth of 50,000 reads per sample. FastQC was used to assess the quality of the raw data. Adapters and poor quality sequences were trimmed with Trimmomatic. Reads were mapped using Bowtie2 [31] and quantitation of reads mapping to the target regions was carried out using Samtools [32]. Samples were normalized to an internal control amplicon (Control3) corresponding to a genomic region that was not differentially methylated in the WGBS discovery cohort (Supplementary Table 2). Digestion by the MSREs was assessed using a different control amplicon (Control1) that had MSRE sites and was consistently unmethylated across sample types in the WGBS discovery cohort and thus would be predicted to be completely digested. Statistical analysis was carried out in R [33] and heatmaps were generated using the R package ComplexHeatmap [34]. Summary statistics from the multiplexed MSRE-PCR assay are available in Supplementary Table 6. Summary statistics for the 1,294 target regions are in Supplementary Table 7.

### Predictive modeling and validation

The MSRE-PCR data was randomly partitioned into training (*n* = 50) and testing (*n* = 50) sets and the R package glmnet [16] was used to fit a logistic regression model on the training set, employing a lasso penalty controlled by the tuning parameter lambda. The optimal value of lambda was determined by K-fold cross-validation with the parameters family set to “binomial”, the number of folds set to 3 and ROC AUC as the type of measurement for model evaluation. The largest value of lambda which yielded an AUC within one standard error of the maximum AUC was chosen, yielding a logistic regression model with 7 markers (Supplementary Table 5). The 7-marker model was validated on the independent test set and ROC AUC and confidence intervals were determined using the pROC R package [35].

### Data accessibility

The WGBS data used in this study was accessed from the NCBI SRA database. Individual accessions used in this study are detailed in Supplementary Table 1. Oligonucleotide sequences used for PCR and hybrid selection are available in Supplementary Tables 2 and 3. MSRE-PCR data is available upon request.

## SUPPLEMENTARY MATERIALS











## Abbreviations

MSRE: Methylation Sensitive Restriction Enzyme
MSRE-PCR: Methylation Sensitive Restriction Enzyme PCR
DMR: Differentially Methylated Region
cDMR: Differentially Methylated Region in cancer
WGBS: Whole Genome Bisulfite Sequencing
cfDNA: Circulating cell-free DNA
ctDNA: Circulating tumor DNA
SRA: Sequence Read Archive
PCA: Principal Component Analysis
ROC: Receiver Operating Characteristic
AUC: Area Under the Curve
