# Supplementary material for: Selective amplification of hypermethylated DNA from diverse tumor types via MSRE-PCR
Source: Oncotarget. 2020 Nov 24;11(47):4387–400. doi: 10.18632/oncotarget.27825 (PMC7720775; doi:10.18632/oncotarget.27825)
Supplement: Supplementary file 1 [file oncotarget-11-4387-s001.pdf]

Selective amplification of hypermethylated DNA from diverse tumor types via MSRE-PCR

SUPPLEMENTARY MATERIALS

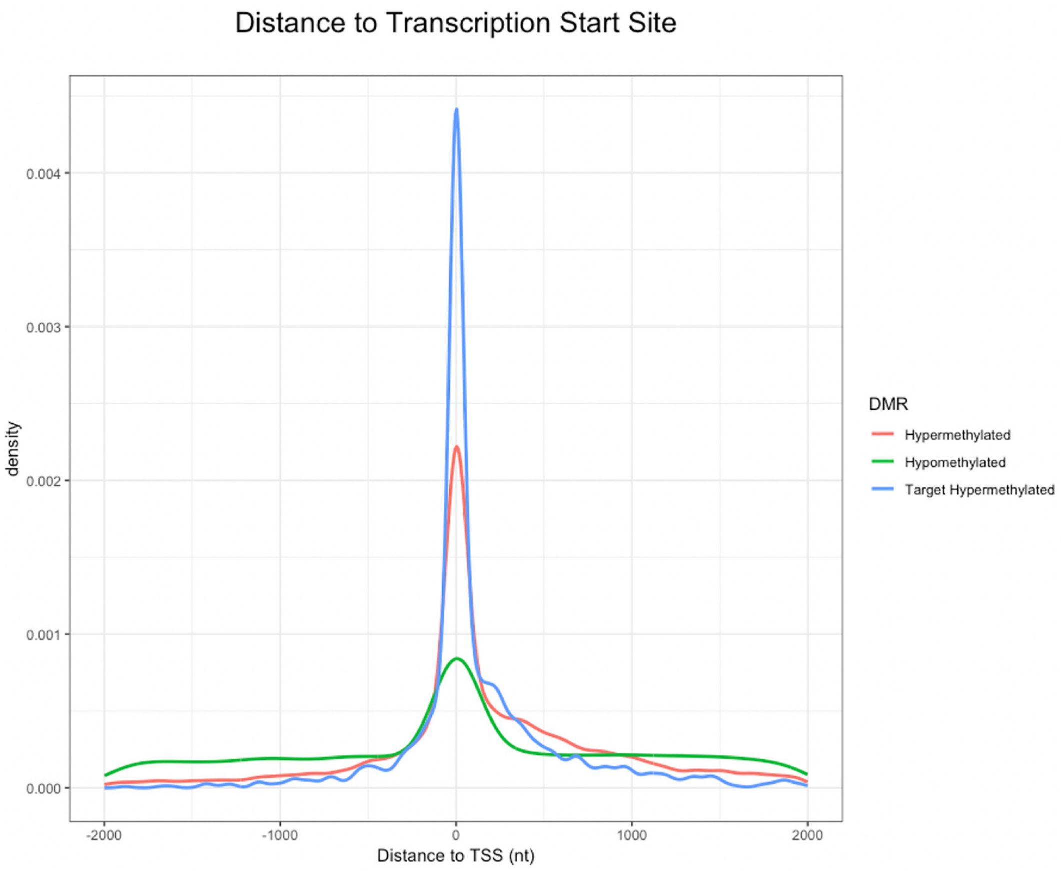

Supplementary Figure 1: DMR distance to the TSS. For the DMRs that are within a range 2000 nt upstream or downstream of the TSS, distance to the TSS is plotted. For the Target regions (shown with the blue line), the mean distance to the promoter is 57 nt, with 39% of the Target regions overlapping the TSS.

Supplementary Table 1: The 48 individual WGBS SRA runs used in this study

Sample Name	Description	MBytes	SRA_Run	SRA_Sample
N3	normal liver	12386	SRR3269805	SRS1351498
		10214	SRR4105107	SRS1351498
N4	normal liver	8200	SRR3269809	SRS1352201
		3908	SRR4105223	SRS1352201
N5	normal lung	20202	SRR3269863	SRS1352208
N6	normal lung	26014	SRR3274240	SRS1353345
N7	normal lung	12576	SRR3274243	SRS1353348
N8	normal liver	24195	SRR3269859	SRS1352204
N9	normal colon	5615	SRR1035727	SRS505928
		5530	SRR1035728	SRS505928
		5574	SRR1035729	SRS505928
		5543	SRR1035730	SRS505928
		5578	SRR1035731	SRS505928
N10	normal blood (B-cells, CD19)	16002	SRR1035894	SRS505949
		11410	SRR1035895	SRS505949
		9563	SRR1035896	SRS505949
N11	normal breast	5906	SRR1035803	SRS505934
		5934	SRR1035804	SRS505934
		6028	SRR1035805	SRS505934
		5731	SRR1035806	SRS505934
		5932	SRR1035807	SRS505934
C3	liver tumor	10862	SRR3269808	SRS1352199
		6121	SRR4105222	SRS1352199
C4	liver tumor	9546	SRR3269811	SRS1352202
		3964	SRR4105224	SRS1352202
C5	lung tumor	20783	SRR3274239	SRS1353344
C6	lung tumor	13149	SRR3274242	SRS1353347
		11646	SRR3306018	SRS1353347
C7	lung tumor	12894	SRR3274244	SRS1353349
C8	liver tumor	10569	SRR3269860	SRS1352206
		13604	SRR3306017	SRS1352206
C9	colon tumor	6019	SRR1035742	SRS505929
		6160	SRR1035743	SRS505929
		6022	SRR1035744	SRS505929
		6078	SRR1035745	SRS505929
		6038	SRR1035746	SRS505929
		6074	SRR1035747	SRS505929
C10	prostate tumor cell line	6628	SRR1035771	SRS505931
		6661	SRR1035772	SRS505931
		6126	SRR1035773	SRS505931
		6562	SRR1035774	SRS505931
		6461	SRR1035775	SRS505931

		6416	SRR1035776	SRS505931
C11	breast tumor cell line	8606	SRR1035790	SRS505933
		8609	SRR1035791	SRS505933
		8649	SRR1035792	SRS505933
		7827	SRR1035793	SRS505933
		8426	SRR1035794	SRS505933

Supplementary Table 2: Description of the multiplexed MSRE-PCR panel. See Supplementary Table 2

Supplementary Table 3: PCR primers for the multiplexed MSRE-PCR panel

Amplicon	Forward Primer	Reverse Primer
Amplicon01	CCTCGAGCGATCTCCTGCCTCA	GCTGGAGGGTCACGGGGACTTA
Amplicon02	TCCCTGTACCGGAGCAGCGATC	CTGCCACCCCGCAAACTTTGGG
Amplicon03	TCGTGACCTCAACGTCCTCCCC	TCAGTCTCGTCCAGAGCGGTGG
Amplicon04	GGGGTTAGCCTCCGGGTAGCAG	CAAAGTGGCCGCTGTGGAGGAG
Amplicon05	CACTTACACTGCACAGGGCCGG	CACTCTGGCCTCGCGAGGGAAG
Amplicon06	CTGCAGCTGCACTTGCTCCGG	ACTCTCGCTGCTCAAGTTCGCG
Amplicon07	GAGGATCACCAGCAGCAGCACC	CTCTGAAGAGGTCCCCGCCCAA
Amplicon08	AACAAAGGCGGCTACGAAGGCC	GGGACTTGGGACGCAGAAGGGA
Amplicon09	CCGATCTGGCCTCCTGCGTTG	TGGAGGATGAAGCCCCCGAGAC
Amplicon10	CGCCACCCACAGAAAGAGCCAG	TCTGCCCTCTTCCCTCTCGCAC
Amplicon11	GCCTGACTCTCCCGGGAGACTC	TCACCTGCGCTCCTCTTCAGCT
Amplicon12	CACCAGCATCCCCACGTTGGTG	CATCAGAGGACCGTCGCCCTCA
Amplicon13	TCGTTCCCCTGAGCGAGACAGG	CCCTGCGGCTCGGGAAGAAAAA
Amplicon14	TCCCGCAAACTTTTCCCGTGGG	GCTGCGGTTCCTTCCCTCGAAG
Amplicon15	TGGGCTTTAGCAGTTTCGGGCG	TGGAAGTCTCTTGAGGGCGCGA
Amplicon16	CTCCCCTCCTCCCCACCACATC	TCCCAAACAGCGGGGAAAAGGC
Amplicon17	GAGCCAGCGCCGTCTCTGAG	CTGCTGCAGCTTCCTGAGTCGG
Amplicon18	CATCGCCATGCACCCTCAGGTC	AAGGCGAGGGTGATGAGCGAGT
Amplicon19	TGTTCGCGCTCATGGACATCGG	TCCAGGGAAGCGCTGACCTCTG
Amplicon20	CTCCAGAGATGTGACCCCCGGG	GTGGAACTGGCGGAAGGTGAGC
Amplicon21	GAGGAGACGGAGGAGGATGCGG	ATCTCAGGAATGGTGCGCGCAG
Amplicon22	CCTTATATCCGGCCCGGCGC	CGCTATAGCAACCGGTGGCTGG
Amplicon23	CAGAGGCTTCGCGGGAGAACG	TTTGCTCTCGCCGAGACCCAGA
Amplicon24	ATCCAGAAGGACCCTCGCGGTG	TCCGAGGACGACGACGGAAGTC
Amplicon25	CAGCTGGAGCGGCGTAGAGC	TGCCTACTTTTCCACGCACCGC
Amplicon26	CACCCTCACTCCACCTAGGGGC	CATCCGCGAGTGTCACTGTGGG
Amplicon27	CGGATTGGAGACTCGAGCGTGC	AAAGTGCTCACGCCGAATCGCA
Amplicon28	TTAGAGACCCCAGCACCCCACG	TAAAGAAGACACGGGCTGCGCG
Amplicon29	TCCCCCTTCTCCCCGGAGATGA	GGAGCTGGGTCTTCTCTCCCCC
Amplicon30	ACCACAAGAGGAAGGCCCCCAG	GACCGCAGTAGGGACCCTCCTC
Amplicon31	GCGACTGGGGCTTGACTGTTCC	CACAAGCCAGGAAAGGACGCGT
Amplicon32	CCCGTTACATAAGGCCACCCCC	CCCTTATCCCCCATGGCCACCA
Amplicon33	TCGACTGGAGAATGGCAGCCCT	GACCAACCTGTCACTCGTCCGC
Amplicon34	GCTTCACGCGGTTCTCATGCCT	AAAACTAGCTGGGCGCGGTAGC
Amplicon35	GAGTCCCCTTCCCTGGAGCCTG	AGTGATGTGGGAGATCCGGGCC
Amplicon36	CCATTTCCCCCGCCCATGGTTC	AAACCTGGCCGGAAAAGACGCG
Amplicon37	CTCCCAGCGCGTGGCCAATAAC	GTGGTGCACCCCTGAAGCGG
Amplicon38	GGGTTGTGTTCCGAGGCCAGTG	GGACGCTGCTTAGCCAGGAACG
Amplicon39	ACGCTGCTAGGCAACATGCTGT	TCTGTTTCAGACACGTTGGCGA
Amplicon40	GCTCCTCCAGATGGAAGCCCCT	CCTAGAGCCGGGCGACTGAGTG
Amplicon41	ACGTGTGTGAGTGTGTGTGCGT	CTGCAAGTGGCACCTCGAGCTC
Amplicon42	TCCTGGGGCTACCTCGCGAG	AGCAAGTTGGGCGAGTTGGCAG
Amplicon43	CACTCCCGGTCTCCACGGCTTA	AGGGCCGAGGAGGAGAGGAC
Amplicon44	AGGACTAGGGGGAGGCGGGTAT	CTTCACCATCAAGCGGCGCTCA
Amplicon45	TATAAAAGTCCCGGACGCGACC	GAGCTCTCCAACTTTCAGCAGC
Amplicon46	TGTGCTCGTTCTGCGCACT	CCAGAGTGAAACTGTGCGTCCT
Amplicon47	CTGGGAATCCAACGTCGCAAAA	GCGCTGTTTTGTTTCCTCCGAA

Control1	TGCAGCGTAGATCCGCCTCGTT	GAGCTGGATAAGATCTATGAGACGCTG
Control2	TACTCTGGGGCATGCAAGTTCTAAAGTTT	GTGTGGTAAAATACCGAGGTGTAAGGAT
Control3	GTGGGGAATGGGTTAGCGTGCC	AGGCACACGTCTGGTCCTGTCA

Supplementary Table 4: Hybrid selection primers. See Supplementary Table 4

Supplementary Table 5: Description of genomic DNAs used in this study. See Supplementary Table 5

Supplementary Table 6: Summary statistics from the multiplexed MSRE-PCR assay

Amplicon	FC	p_adjusted	trainAUC	testAUC	coef_7-marker_model
Amplicon01	71	2E-04	0.83	0.57	.
Amplicon02	152	3E-03	0.71	0.78	.
Amplicon03	70	1E-01	0.60	0.74	.
Amplicon04	15	4E-03	0.75	0.76	.
Amplicon07	3	4E-01	0.56	0.67	.
Amplicon08	24	2E-01	0.60	0.68	.
Amplicon09	104	3E-05	0.88	0.77	1.01E-04
Amplicon10	20	3E-01	0.60	0.71	.
Amplicon13	287	5E-03	0.70	0.82	6.12E-04
Amplicon14	2	4E-01	0.57	0.69	.
Amplicon15	16	8E-02	0.65	0.66	.
Amplicon16	59	3E-05	0.88	0.91	2.17E-05
Amplicon18	11	3E-05	0.89	0.93	.
Amplicon20	67	1E-03	0.77	0.68	.
Amplicon24	14	2E-03	0.78	0.69	.
Amplicon26	36	7E-03	0.73	0.67	.
Amplicon27	4	6E-01	0.54	0.59	.
Amplicon28	26	7E-02	0.65	0.75	.
Amplicon29	30	6E-03	0.75	0.74	3.89E-05
Amplicon30	20	9E-03	0.67	0.62	2.79E-03
Amplicon31	39	4E-05	0.87	0.86	.
Amplicon32	11	4E-03	0.73	0.52	7.75E-04
Amplicon33	23	5E-02	0.65	0.73	.
Amplicon34	9	6E-02	0.67	0.59	.
Amplicon35	21	1E-03	0.79	0.67	.
Amplicon36	204	3E-04	0.81	0.65	.
Amplicon37	13	4E-01	0.58	0.60	.
Amplicon38	4	6E-02	0.67	0.66	.
Amplicon39	6	4E-01	0.54	0.64	.
Amplicon40	6	8E-06	0.89	0.85	.
Amplicon41	76	1E-04	0.84	0.82	.
Amplicon42	24	2E-01	0.61	0.67	.
Amplicon43	3	1E-01	0.64	0.68	.
Amplicon44	16	2E-04	0.83	0.73	.
Amplicon47	18	1E-03	0.80	0.75	5.38E-04

The 35 amplicons with an average of > 10 reads per sample are shown. Definitions: FC: the fold-change difference in amplification levels in the tumor samples versus normal tissue samples in the training set; *p*_adjusted: the FDR corrected *p*-value derived from the Mann-Whitney *U* test comparing multiplexed MSRE-PCR amplification in the tumor samples versus normal tissue samples in the training set; trainAUC: the ROC AUC for the individual amplicon in the multiplexed MSRE-PCR training data set; testAUC: the ROC AUC for the individual amplicon in the multiplexed MSRE-PCR test data set; coef_7-marker_model: the coefficients for the 7-marker logistic regression model (Intercept = -7.82E-01).

Supplementary Table 7: All 1294 target regions: chromosomal location, region size, number of CpGs contained within the region, the methylation difference between cancer samples versus normal tissue samples, the mean methylation level in the cancer samples and the mean methylation in the normal samples. See Supplementary Table 7
