# Supplementary material for: Selective amplification of hypermethylated DNA from diverse tumor types via MSRE-PCR
Source: Oncotarget. 2020 Nov 24;11(47):4387–400. doi: 10.18632/oncotarget.27825 (PMC7720775; doi:10.18632/oncotarget.27825)
Supplement: Supplementary file 2 [file oncotarget-11-4387-s002.docx]

**Supplementary Table 2: Description of the multiplexed MSRE-PCR panel**

| **Amplicon** | **Interval** | **Gene** | **Length** | **MSRE Sites** | **CpGs** |
| --- | --- | --- | --- | --- | --- |
| Amplicon01 | chr12:64824626-64824737 | TBC1D30 | 112 | 4 | 10 |
| Amplicon02 | chr7:50304321-50304442 | IKZF1 | 122 | 6 | 16 |
| Amplicon03 | chr2:143937200-143937289 | LOC101928386 | 90 | 5 | 8 |
| Amplicon04 | chr5:178590308-178590386 | COL23A1 | 79 | 5 | 9 |
| Amplicon05 | chr8:92102850-92102988 | RUNX1T1 | 139 | 9 | 18 |
| Amplicon06 | chr7:3301851-3301957 | SDK1 | 107 | 11 | 17 |
| Amplicon07 | chr10:16521042-16521191 | C1QL3 | 150 | 6 | 10 |
| Amplicon08 | chr2:1743986-1744131 | PXDN | 146 | 10 | 17 |
| Amplicon09 | chr14:101781745-101781848 | PPP2R5C | 104 | 8 | 13 |
| Amplicon10 | chr19:31348851-31348989 | TSHZ3 | 139 | 8 | 10 |
| Amplicon11 | chr1:240092007-240092150 | FMN2 | 144 | 9 | 12 |
| Amplicon12 | chr19:50050788-50050922 | LOC400710 | 135 | 7 | 12 |
| Amplicon13 | chr3:143120231-143120369 | CHST2 | 139 | 9 | 19 |
| Amplicon14 | chr2:134718424-134718544 | TMEM163 | 121 | 6 | 11 |
| Amplicon15 | chr1:8217451-8217528 | LINC01714 | 78 | 5 | 8 |
| Amplicon16 | chr5:10564880-10564985 | ANKRD33B | 106 | 8 | 9 |
| Amplicon17 | chr12:52006980-52007052 | GRASP | 73 | 5 | 8 |
| Amplicon18 | chr7:5593341-5593478 | FSCN1 | 138 | 9 | 21 |
| Amplicon19 | chr1:1540328-1540456 | TMEM240 | 129 | 15 | 19 |
| Amplicon20 | chr3:129001940-129002066 | EFCC1 | 127 | 10 | 15 |
| Amplicon21 | chr3:71753745-71753865 | GPR27 | 121 | 8 | 13 |
| Amplicon22 | chr7:157690941-157691013 | PTPRN2 | 73 | 16 | 16 |
| Amplicon23 | chr9:113801-113894 | LINC01388 | 94 | 9 | 15 |
| Amplicon24 | chr19:58440344-58440471 | ZNF132 | 128 | 2 | 11 |
| Amplicon25 | chr17:9003275-9003375 | NTN1 | 101 | 6 | 13 |
| Amplicon26 | chr18:25350740-25350873 | ZNF521 | 134 | 9 | 16 |
| Amplicon27 | chr15:62164836-62164918 | C2CD4B | 83 | 3 | 10 |
| Amplicon28 | chr12:65741959-65742068 | RPSAP52 | 110 | 8 | 12 |
| Amplicon29 | chr18:79798537-79798656 | KCNG2 | 120 | 6 | 11 |
| Amplicon30 | chr1:32754104-32754235 | KIAA1522 | 132 | 4 | 14 |
| Amplicon31 | chr1:161306009-161306146 | MPZ | 138 | 7 | 10 |
| Amplicon32 | chr5:132656449-132656585 | TH2LCRR | 137 | 12 | 16 |
| Amplicon33 | chr7:49773380-49773465 | VWC2 | 86 | 5 | 10 |
| Amplicon34 | chr6:26044563-26044639 | HIST1H3C | 77 | 4 | 5 |
| Amplicon35 | chr12:4164771-4164885 | CCND2 | 115 | 5 | 11 |
| Amplicon36 | chr6:130365323-130365437 | SAMD3 | 115 | 7 | 13 |
| Amplicon37 | chr18:72543593-72543674 | CBLN2 | 82 | 13 | 12 |
| Amplicon38 | chr2:26299121-26299246 | LOC105374334 | 126 | 3 | 6 |
| Amplicon39 | chr12:132908549-132908618 | ZNF605 | 70 | 3 | 6 |
| Amplicon40 | chr11:128694827-128694902 | FLI1 | 76 | 2 | 4 |
| Amplicon41 | chr12:106583572-106583687 | RFX4 | 116 | 9 | 16 |
| Amplicon42 | chr3:192409743-192409861 | FGF12 | 119 | 6 | 11 |
| Amplicon43 | chr8:9906512-9906587 | MIR124-1 | 76 | 5 | 8 |
| Amplicon44 | chr1:196608625-196608728 | KCNT2 | 104 | 4 | 8 |
| Amplicon45 | chr1:166165011-166165143 | FAM78B | 133 | 11 | 16 |
| Amplicon46 | chr17:77373794-77373904 | LOC112268276 | 133 | 3 | 9 |
| Amplicon47 | chr11:13962548-13962619 | SPON1 | 72 | 6 | 9 |
| Control1 | chr3:138174642-138174763 | DBR1 | 121 | 10 | 15 |
| Control2 | chr3:138171581-138171704 | DBR1 | 124 | 0 | 3 |
| Control3 | chr5:226107-226224 | SDHA | 118 | 3 | 6 |
