# Supplementary material for: Selective amplification of hypermethylated DNA from diverse tumor types via MSRE-PCR
Source: Oncotarget. 2020 Nov 24;11(47):4387–400. doi: 10.18632/oncotarget.27825 (PMC7720775; doi:10.18632/oncotarget.27825)
Supplement: Supplementary file 3 [file oncotarget-11-4387-s003.docx]

**Supplementary Table 4: Hybrid selection primers**

| **Name** | **HybridSelection_Primer** |
| --- | --- |
| Amplicon01 | CCTCAGCCTTGCAGGCTCCGCACTGCAGATGCCTGCTGGCTTCCCTGCGCTCGGCGGCTCCCGCGGTGCCCCGTAAGTC |
| Amplicon02 | CGATCCGGGAGGCGGCCGAGAGGTGCGCGCGGGGCCGAGCCGGCTGCGGGGCAGGTCGAGCAGGGACCGCCAGCGTGCGTCACCCCAAA |
| Amplicon03 | TCCCCCGTCCCTCGACATGTTCCCGCGCCTGAAAGGTGCAGAAAACTCCCCCCACCGNNN |
| Amplicon04 | GCAGCGGATCGCCGCGCACGCCCCCTTCGCCGCAGCCAGCTCCTCCNNNNNNNNNNNNNN |
| Amplicon05 | GCCGGAGAGTCCCACCATCCGCCACAGGCTCCGAGCTGCAAATAAAACTTCCCGTCGTCTCGGCCGGCCCGCGGGGCGACGGGGCGAGGACGCGGCGCGGCTTCCC |
| Amplicon06 | CCGGGCGCTGGCGCAAGGTAGGTGCGCGCGGGGTCGCGGGCCGGGGGCGTCGCCTCGGGGCGCGGAGGCGCGAA |
| Amplicon07 | GCACCATCACCACCCCCAGCGCCCCGGCGGCGATCAGGCGCCTCCTGCTGCCCACCAGCCGGCTCAGCGCGGGGCGATCCTCTTGTCTGCTTTTGGACAGAATTTTGAAGGTTGGGC |
| Amplicon08 | AGGCCGCGCGCACAGCGCTCTGTTCCGCCCCTCGGCGTCTCCCGCAGGGCGAACAAAGGCCCAGCGGGTCCCCGCGCCCAGGTCCCCCCACGTCCCCCTTCGGAGGTTCCCTT |
| Amplicon09 | GTTGCGCGCTCCAACCCTCTGCTTGGCCGCCCGCGAACCGCGCTCTCCCGTTTCCTTTCCGTCCCGTCTCG |
| Amplicon10 | GCCAGGCCGGGGTTGCTTCCCATTCCCTCTGCAGCCGGAGAGCTGAGGAGGTAGGGACCTGGCGCGGCTCAGCGCGCTCCGCGAGCGGCTCCCCAAATGGGTGCGA |
| Amplicon11 | GACTCCCTAGGCCCGGACCTGGGGCCGAGGAGGGCCGGGATGGCCTGAGTGCCCGCGGCGCGGCGGCGCAGCAGCGGGATTGCACCATGGGGAACCAGGATGGGAAGCTGA |
| Amplicon12 | GGTGGCGACGCTGCCCCGGCCCCACGGATACTTCCGCGCCTGTCAGACTCCCTGATGAACTACCCTTCCCAGAGTACCGCGGGAGCTCGGGCTCCTGAGGGC |
| Amplicon13 | ACAGGAAGCTGCGGTCCCGAGAAAGCGGAGGAGACGTCGCTGGAGCCGGGAGGCGCCGGGTTCGGCGGAGCGCGGAGCGGGGCTCTGGGCCGCGTGAAAGTTTTTC |
| Amplicon14 | GTGGGCGCCGGAGGGCCTGGAGCTGGCGGAGAGGAGGGCGCGAGTCCAGGCGGGGTCGGCCTGAAGGCAGCTGGGGTCTGGGCTTCGA |
| Amplicon15 | GGGCGTGCACGTGCGCGGAGGCTCCTGCCTCTGCGGAGCTCGCGCNNNNNNNNNNNNNNN |
| Amplicon16 | CATCCCCGCGCCTCCCAGCTCCTGGCTCCGGACCGGTCCCTCGGTCACTCAGCCGCCGCCCAGAGCGCCTTTT |
| Amplicon17 | GAGGGGCGTCCGGCGCCGGAGCCATGACCCTCCGCCGACTNNNNNNNNNNNNNNNNNNNN |
| Amplicon18 | AGGTCAACATCTACAGCGTCACCCGTAAGCGCTACGCGCACCTGAGCGCGCGGCCGGCCGACGAGATCGCCGTGGACCGCGACGTGCCCTGGGGCGTCGACTCGC |
| Amplicon19 | ATCGGGCGGGGCCGGGCCGGGCCGGAGCGCCGCCCCCCGGCCCCGGCGCCCCCCCGGCCCCGGCCCGATGCTGAGCCCCCGCCGCCTCCGCAGAGG |
| Amplicon20 | CCGGGGATGCGGCCGCTGAGTTGGCCACGGACGGGGACTCAGATACCGATGAAGAGGCGCGCCTGGCGCTGCGCGCCGAGCCGCCGGAGCTCAC |
| Amplicon21 | TGCGGGGGGCTGCGGACGGTGGCGAGGCCCTGTCCGCAGTGAAGCCCGCGGCGCCTAGGGCGCAGGGAGTGGCCGCCTCCTCCTGCGC |
| Amplicon22 | CGTAGCACCAACCAGCGCGGCCGCCGCGCCCCGCCTTATANNNNNNNNNNNNNNNNNNNN |
| Amplicon23 | AACGCGCAGCCCGGGGCGTGGGGCGGGGAACTGCCCGCGCGAGGCTTTCGGCGCGTCTGGG |
| Amplicon24 | CGGTGCCTCAGGGCTGGCCATTGGCAGCCGAGGAGACAGGCACTTCCGGGCGGAGTGTAAGACGCTGGCCAATCACAGCCTGGCAGCGGGACTTC |
| Amplicon25 | AGCCTGCGCGGGGATGGCGGGGGTGGCCGCGGGCCGGGGCCGTCTGGGGGCGGCCATCCCGGGCGGTG |
| Amplicon26 | GGGGCTCAGCGGGGAGGGGAGCGCGCGCGCCCCCGCACTCACACTCACACTCACGCGCGCACACGCCTCGCAGCCACGCAGCCCTCGCTCCGCTACCCACA |
| Amplicon27 | CGTGCAAGGGGCCGGCAGCCGCGGCGGGATGCAGAATTCGGGGATGCGATNNNNNNNNNN |
| Amplicon28 | CCACGCAGACGGCCCGGGCTTAGACCTCTCCACGTCGCTGTGGCGCGCGCCCCGCCAACACCCCCACCCCACGCGCA |
| Amplicon29 | GATGAACGCCCCACTCCCGCTCGGACACACGGCCGCACCCCTACTCTGCCTCCTCCGACCAGCCGGGGCGCGGCCCGAATTGGGGGA |
| Amplicon30 | CCAGCGGTCCCCGGGTCCGCAGCTTCTGGCGGTTCGGGCGGTCGGCGAAGCGGCCGGCAGGTAGGGGCCGGGGTGGGGGCGGGGCAGGCAGGGAGGAGG |
| Amplicon31 | GTTCCCATCCCACCCCTCACTGCTGCCCGGCGGCTCCCAGGGTTCTCCTTCCCATCTTGTCTAGGCCCCAAGTCCCGCTAACCTGCCGCCCGCGCTTCGACGCGT |
| Amplicon32 | CCCCCCTATCTCCGCGGGCCATCGCCGCCGCAACCGCCGCGCCAGCGCCTTCTCCCACGCGCGGGGGCGCCCCTGCCCACCGCTCCCGGCAGGGCTTTTGGTGG |
| Amplicon33 | GCCCTGGCAGGGTCTGCGGCCGGCTGCGGAGGTGGGGACGCGGCCGCGCGGACNNNNNNN |
| Amplicon34 | TGCCTCAGCCTCCTGTGTACTTGGGATTACAGGCGTCTGCTACCNNNNNNNNNNNNNNNN |
| Amplicon35 | GCCTGGGGGACCGACAGTCGCGGGCCACGCACGCGGTCCCAGCCTCGCCGTCCGCCCCGGGGAGAGCTGGAAGGTGGGCCCG |
| Amplicon36 | GGTTCTCGCCCCCCCAAGCCGTTCTCCGGAACCCCTTGCCGGGCCGGCGGGAAGCGTGGACGGAGCGGGCCTTGGCCGCGTC |
| Amplicon37 | TAACCGCGCCGCCCCGCCCTGCCGCTTTCCCGCGCCAGCCTGCGCCGCTNNNNNNNNNNN |
| Amplicon38 | CAGTGTGGGGGAAACCAGACCTAGAACGTTGTATACGGCTGATAGGACCAGGTCCACCGCTGCAGCAGGCTGTGAGAGGGGAAGGCACGTTCC |
| Amplicon39 | GCTGTCTCCCGGCAACGTAGAGGCGGGGAATTCGCCANNNNNNNNNNNNNNNNNNNNNNN |
| Amplicon40 | CCCTGTTTACATGTCAGCGCTTTCCCTTCCTCTCGGCACTCAGNNNNNNNNNNNNNNNNN |
| Amplicon41 | TGCGTGCGCGCGCGCGAAGGAGCGCGCTTTCGCTTGCCCTGGACACAAAGTTACGTAAAGATGCGCTGGCTCGCGGCGAGCTC |
| Amplicon42 | GAGGCAGCCGAGGGCGCAACCCGGGCGCTTGGGGCCGGAGGCGGAATCAGGGGCCGGGGCCAGGAGGCAGGTGCAGGCGGCTGCCA |
| Amplicon43 | GCTTAGGCAGACGGAGTGGGGGACTCCGGGGACCCGCGCGTCCNNNNNNNNNNNNNNNNN |
| Amplicon44 | GGTATAAAAAGTTACTTCGCCGAAAAAGACGCGCGGTGCCTAGAAAGAGGGGAGAGGGGTGGGAGTGAGCG |
| Amplicon45 | CGACCGCCCGTAGCCTGCGGCCCCTGGCGGTTGAGGAGCCGCGGCGATCCTTGGTCTTCACCGCAGCAGCCAAGCCCGGCGCGCACCAACCGGCGCTGCT |
| Amplicon46 | TGCCGCCTGGGTTTCCTTCCTTTTATTGTTGTTTGTGTTTGCCAAGCGACAGCGACCTCCTCGAGGGCTCGCGAGGCT |
| Amplicon47 | CAAAATAAACACGGCCGCGCCGCTAATCGCCAGTTCGGANNNNNNNNNNNNNNNNNNNNN |
| Control1 | TCGTTGCGCACCGCCTGGAAGTCGCCGCAGCACAGCAAGAGGTCGACAGGCCCCGGGCCGCGCCGCTCTGCCAGCGCCAGCGTCTCATA |
| Control2 | GTTCTAAAGTTTAAAAATAATTCTCAAAACAATACCTTTTCGATAGTCATGAGATTTAAAGATACCAGAGATTCCACCGATCCTTACACCT |
| Control3 | TGCCCAGTGAGTCAGCCAGAGATTACGTAAAAAGCAACAGAGAACAGCAGCGTGGGGCGCATGCAGCGACTGTGGATGTGACAGG |
