# Supplementary material for: Selective amplification of hypermethylated DNA from diverse tumor types via MSRE-PCR
Source: Oncotarget. 2020 Nov 24;11(47):4387–400. doi: 10.18632/oncotarget.27825 (PMC7720775; doi:10.18632/oncotarget.27825)
Supplement: Supplementary file 4 [file oncotarget-11-4387-s004.docx]

**Supplementary Table 5: Description of genomic DNAs used in this study**

| **ID** | **Well** | **Tissue** | **Status** | **Description** | **Age** | **Sex** |
| --- | --- | --- | --- | --- | --- | --- |
| Seq119 | A1 | Breast | Normal | Normal | 62 | F |
| Seq120 | C1 | Breast | Normal | Normal | 45 | F |
| Seq121 | E1 | Breast | Normal | Normal | 34 | F |
| Seq122 | G1 | Breast | Normal | Normal | 83 | F |
| Seq123 | A2 | Breast | Normal | Normal | 78 | F |
| Seq124 | C2 | Breast | Normal | Normal | 76 | F |
| Seq125 | E2 | Breast | Normal | Normal | 64 | F |
| Seq126 | G2 | Breast | Normal | Normal | 60 | F |
| Seq127 | C4 | Breast | Tumor | Invasive Ductal Carcinoma | 48 | F |
| Seq128 | E4 | Breast | Tumor | Invasive Ductal Carcinoma | 67 | F |
| Seq129 | G4 | Breast | Tumor | Invasive Ductal Carcinoma | 40 | F |
| Seq130 | E5 | Breast | Tumor | Invasive Ductal Carcinoma | 77 | F |
| Seq131 | G5 | Breast | Tumor | Invasive Ductal Carcinoma | 42 | F |
| Seq132 | A6 | Breast | Tumor | Invasive Ductal Carcinoma | 47 | F |
| Seq133 | C6 | Breast | Tumor | Invasive Ductal Carcinoma | 34 | F |
| Seq134 | E6 | Breast | Tumor | Invasive Ductal Carcinoma | 56 | F |
| Seq135 | G6 | Breast | Tumor | Invasive Ductal Carcinoma | 46 | F |
| Seq136 | A7 | Breast | Tumor | Invasive Ductal Carcinoma | 57 | F |
| Seq137 | C7 | Breast | Tumor | Invasive Ductal Carcinoma | 36 | F |
| Seq138 | E7 | Breast | Tumor | Invasive Ductal Carcinoma | 37 | F |
| Seq035 | B1 | lung | Normal | Normal | 23 | M |
| Seq036 | D1 | lung | Normal | Normal | 33 | M |
| Seq037 | F1 | lung | Normal | Normal | 23 | M |
| Seq041 | H1 | lung | Normal | Normal | 33 | M |
| Seq042 | B2 | lung | Normal | Normal | 36 | M |
| Seq043 | D2 | lung | Normal | Normal | 24 | M |
| Seq044 | F2 | lung | Normal | Normal | 70 | F |
| Seq045 | H2 | lung | Normal | Normal | 26 | M |
| Seq038 | B3 | lung | Tumor | Adenocarcinoma, Moderately Differentiated | 44 | M |
| Seq039 | D3 | lung | Tumor | Adenocarcinoma, Poorly Differentiated | 48 | F |
| Seq040 | F3 | lung | Tumor | Adenocarcinoma, Well Differentiated | 42 | M |
| Seq046 | H3 | lung | Tumor | Adenocarcinoma, Well Differentiated | 64 | F |
| Seq047 | B4 | lung | Tumor | Adenocarcinoma, Moderately Differentiated | 39 | F |
| Seq048 | D4 | lung | Tumor | Adenocarcinoma, Moderately Differentiated | 50 | M |
| Seq049 | F4 | lung | Tumor | Adenocarcinoma, Moderately Differentiated | 70 | M |
| Seq050 | H4 | lung | Tumor | Adenocarcinoma, Moderately Differentiated | 70 | M |
| Seq051 | B5 | lung | Tumor | Adenocarcinoma, Poorly Differentiated | 66 | M |
| Seq052 | B7 | lung | Tumor | Squamous cell carcinoma, Moderately Differentiated | 47 | M |
| Seq053 | D7 | lung | Tumor | Squamous cell carcinoma, Moderately Differentiated | 53 | M |
| Seq054 | F7 | lung | Tumor | Squamous cell carcinoma, Poorly Differentiated | 53 | M |
| Seq139 | A1 | Colon | Normal | Normal | 87 | M |
| Seq140 | C1 | Colon | Normal | Normal | 72 | F |
| Seq141 | E1 | Colon | Normal | Normal | 30 | M |
| Seq142 | G1 | Colon | Normal | Normal | 21 | M |
| Seq143 | A2 | Colon | Normal | Normal | 41 | M |
| Seq144 | C2 | Colon | Normal | Normal | 25 | M |
| Seq145 | E2 | Colon | Normal | Normal | 23 | M |
| Seq146 | G2 | Colon | Normal | Normal | 34 | M |
| Seq147 | C4 | Colon | Tumor | Adenocarcinoma, Moderately Differentiated | 61 | M |
| Seq148 | E4 | Colon | Tumor | Adenocarcinoma, Moderately Differentiated | 77 | F |
| Seq149 | G4 | Colon | Tumor | Adenocarcinoma, Moderately Differentiated | 46 | F |
| Seq150 | A5 | Colon | Tumor | Adenocarcinoma, Moderately Differentiated | 46 | M |
| Seq151 | C5 | Colon | Tumor | Adenocarcinoma, Moderately Differentiated | 81 | M |
| Seq152 | E5 | Colon | Tumor | Adenocarcinoma, Moderately Differentiated | 57 | M |
| Seq153 | G5 | Colon | Tumor | Adenocarcinoma, Moderately Differentiated | 68 | M |
| Seq154 | A6 | Colon | Tumor | Adenocarcinoma, Moderately Differentiated | 57 | M |
| Seq155 | C6 | Colon | Tumor | Adenocarcinoma, Moderately Differentiated | 41 | M |
| Seq156 | E6 | Colon | Tumor | Adenocarcinoma, Moderately Differentiated | 62 | M |
| Seq157 | G6 | Colon | Tumor | Adenocarcinoma, Moderately Differentiated | 62 | F |
| Seq158 | A7 | Colon | Tumor | Adenocarcinoma, Moderately Differentiated | 60 | M |
| Seq159 | A1 | Stomach | Normal | Normal | 83 | F |
| Seq160 | C1 | Stomach | Normal | Normal | 33 | M |
| Seq161 | E1 | Stomach | Normal | Normal | 66 | M |
| Seq162 | G1 | Stomach | Normal | Normal | 58 | M |
| Seq163 | A2 | Stomach | Normal | Normal | 71 | M |
| Seq164 | C2 | Stomach | Normal | Normal | 79 | M |
| Seq165 | E2 | Stomach | Normal | Normal | 24 | M |
| Seq166 | G2 | Stomach | Normal | Normal | 50 | M |
| Seq167 | C4 | Stomach | Tumor | Adenocarcinoma | 59 | F |
| Seq168 | E4 | Stomach | Tumor | Adenocarcinoma | 69 | F |
| Seq169 | G4 | Stomach | Tumor | Adenocarcinoma | 60 | F |
| Seq170 | A5 | Stomach | Tumor | Adenocarcinoma | 63 | M |
| Seq171 | C5 | Stomach | Tumor | Adenocarcinoma | 61 | M |
| Seq172 | E5 | Stomach | Tumor | Adenocarcinoma | 62 | F |
| Seq173 | G5 | Stomach | Tumor | Adenocarcinoma | 66 | M |
| Seq174 | A6 | Stomach | Tumor | Adenocarcinoma | 74 | M |
| Seq175 | C6 | Stomach | Tumor | Adenocarcinoma | 65 | M |
| Seq176 | E6 | Stomach | Tumor | Adenocarcinoma | 41 | M |
| Seq177 | G6 | Stomach | Tumor | Adenocarcinoma | 70 | M |
| Seq178 | A7 | Stomach | Tumor | Adenocarcinoma | 56 | M |
| Seq179 | A1 | Blood | Normal | Normal | 50 | F |
| Seq180 | A2 | Blood | Normal | Normal | 62 | F |
| Seq181 | A3 | Blood | Normal | Normal | 59 | M |
| Seq182 | A4 | Blood | Normal | Normal | 77 | M |
| Seq183 | A5 | Blood | Normal | Normal | 58 | F |
| Seq184 | A6 | Blood | Normal | Normal | 45 | F |
| Seq185 | A7 | Blood | Normal | Normal | 59 | F |
| Seq186 | A8 | Blood | Normal | Normal | 66 | M |
| Seq187 | A9 | Blood | Normal | Normal | 66 | M |
| Seq188 | A10 | Blood | Normal | Normal | 28 | M |
| Seq189 | A11 | Blood | Normal | Normal | 60 | M |
| Seq190 | A12 | Blood | Normal | Normal | 55 | M |
| Seq191 | C1 | Blood | Normal | Normal | 55 | M |
| Seq192 | C2 | Blood | Normal | Normal | 54 | M |
| Seq193 | C3 | Blood | Normal | Normal | 28 | F |
| Seq194 | C4 | Blood | Normal | Normal | 37 | M |
| Seq195 | C5 | Blood | Normal | Normal | 29 | M |
| Seq196 | C6 | Blood | Normal | Normal | 38 | M |
| Seq197 | C7 | Blood | Normal | Normal | 40 | M |
| Seq198 | C8 | Blood | Normal | Normal | 50 | F |

All DNAs were sourced from Biochain (Newark, CA, USA), catalog numbers D8235152-1, D8235086-1, D8235090-1, D8234148-1 and D8235248-1.
