# Supplementary material for: Selective amplification of hypermethylated DNA from diverse tumor types via MSRE-PCR
Source: Oncotarget. 2020 Nov 24;11(47):4387–400. doi: 10.18632/oncotarget.27825 (PMC7720775; doi:10.18632/oncotarget.27825)
Supplement: Supplementary file 5 [file oncotarget-11-4387-s005.docx]

**Supplementary Table 7: All 1294 target regions: chromosomal location, region size, number of CpGs contained within the region, the methylation difference between cancer samples versus normal tissue samples, the mean methylation level in the cancer samples and the mean methylation in the normal samples**

| **chr** | **start** | **stop** | **region size** | **number CpGs** | **Q-value** | **mean meth difference** | **mean meth cancer** | **mean meth normal** |
| --- | --- | --- | --- | --- | --- | --- | --- | --- |
| 1 | 910141 | 910314 | 173 | 47 | 0.023 | 17 | 20 | 2.2 |
| 1 | 998858 | 999205 | 347 | 77 | 2.50E-09 | 17 | 18 | 0.5 |
| 1 | 1540198 | 1540534 | 336 | 70 | 7.00E-40 | 36 | 38 | 2.2 |
| 1 | 1599537 | 1599887 | 350 | 122 | 4.30E-09 | 16 | 17 | 1.4 |
| 1 | 3068592 | 3068839 | 247 | 68 | 3.10E-05 | 17 | 18 | 1.1 |
| 1 | 3070266 | 3070498 | 232 | 81 | 0.0002 | 13 | 14 | 0.6 |
| 1 | 3611355 | 3611514 | 159 | 44 | 9.40E-05 | 19 | 20 | 0.7 |
| 1 | 4654486 | 4654592 | 106 | 29 | 0.00013 | 16 | 18 | 2.1 |
| 1 | 4655292 | 4655415 | 123 | 47 | 9.80E-12 | 22 | 24 | 2.4 |
| 1 | 4656010 | 4656140 | 130 | 33 | 2.40E-09 | 19 | 21 | 2.4 |
| 1 | 4656393 | 4656644 | 251 | 38 | 0.022 | 11 | 12 | 1.6 |
| 1 | 6179529 | 6179694 | 165 | 54 | 0.00014 | 16 | 18 | 1.8 |
| 1 | 6205451 | 6206068 | 617 | 91 | 0.039 | 14 | 16 | 2.4 |
| 1 | 6448969 | 6449116 | 147 | 36 | 0.02 | 14 | 15 | 0.9 |
| 1 | 8217209 | 8217762 | 553 | 131 | 2.80E-44 | 24 | 26 | 2.1 |
| 1 | 10888877 | 10888985 | 108 | 38 | 6.20E-12 | 18 | 20 | 2.3 |
| 1 | 11690605 | 11691586 | 981 | 194 | 4.60E-07 | 12 | 14 | 1.9 |
| 1 | 13513557 | 13513639 | 82 | 32 | 0.0086 | 17 | 19 | 1.9 |
| 1 | 13700193 | 13700312 | 119 | 46 | 0.001 | 18 | 19 | 1.0 |
| 1 | 14599323 | 14599497 | 174 | 36 | 0.01 | 15 | 16 | 1.2 |
| 1 | 14924291 | 14924746 | 455 | 163 | 3.20E-06 | 13 | 14 | 0.9 |
| 1 | 15758690 | 15759223 | 533 | 121 | 1.00E-31 | 22 | 24 | 2.1 |
| 1 | 20483787 | 20484637 | 850 | 128 | 3.30E-10 | 20 | 22 | 2.2 |
| 1 | 20731673 | 20731869 | 196 | 47 | 5.20E-09 | 16 | 18 | 2.3 |
| 1 | 21290222 | 21290498 | 276 | 74 | 4.90E-13 | 15 | 15 | 0.8 |
| 1 | 23568180 | 23568507 | 327 | 64 | 0.019 | 15 | 17 | 1.4 |
| 1 | 24321897 | 24322153 | 256 | 60 | 1.20E-07 | 20 | 22 | 1.7 |
| 1 | 28592226 | 28592564 | 338 | 67 | 0.00023 | 13 | 13 | 0.5 |
| 1 | 28592564 | 28592810 | 246 | 69 | 7.10E-07 | 15 | 16 | 1.0 |
| 1 | 28812056 | 28812605 | 549 | 178 | 3.70E-07 | 13 | 15 | 2.1 |
| 1 | 29121903 | 29122179 | 276 | 72 | 8.00E-11 | 18 | 19 | 1.6 |
| 1 | 31760615 | 31760933 | 318 | 63 | 2.60E-09 | 23 | 25 | 2.0 |
| 1 | 32465342 | 32465460 | 118 | 30 | 0.0079 | 12 | 14 | 1.9 |
| 1 | 32753644 | 32754259 | 615 | 123 | 2.00E-35 | 23 | 25 | 2.2 |
| 1 | 34929821 | 34929919 | 98 | 26 | 0.016 | 17 | 19 | 2.4 |
| 1 | 37034352 | 37034538 | 186 | 59 | 1.30E-11 | 17 | 19 | 1.5 |
| 1 | 38046438 | 38046783 | 345 | 118 | 3.90E-15 | 20 | 21 | 1.4 |
| 1 | 38804120 | 38804212 | 92 | 38 | 2.10E-06 | 20 | 22 | 2.0 |
| 1 | 39788980 | 39789115 | 135 | 39 | 0.032 | 15 | 16 | 1.0 |
| 1 | 39789169 | 39789457 | 288 | 61 | 3.40E-12 | 16 | 19 | 2.3 |
| 1 | 41382455 | 41382607 | 152 | 44 | 0.021 | 18 | 20 | 1.7 |
| 1 | 44784226 | 44784672 | 446 | 109 | 5.10E-09 | 20 | 22 | 2.3 |
| 1 | 44842950 | 44843437 | 487 | 107 | 0.0012 | 12 | 13 | 1.4 |
| 1 | 45326846 | 45327156 | 310 | 55 | 0.0008 | 15 | 16 | 1.1 |
| 1 | 52602160 | 52602325 | 165 | 33 | 8.10E-11 | 25 | 27 | 2.1 |
| 1 | 52633075 | 52633229 | 154 | 40 | 0.022 | 17 | 19 | 1.9 |
| 1 | 53062240 | 53062609 | 369 | 62 | 8.30E-11 | 19 | 21 | 2.3 |
| 1 | 56477405 | 56478036 | 631 | 108 | 2.90E-06 | 19 | 21 | 1.8 |
| 1 | 57423822 | 57424080 | 258 | 67 | 0.005 | 15 | 17 | 2.0 |
| 1 | 58781635 | 58782738 | 1103 | 172 | 2.10E-14 | 17 | 17 | 0.6 |
| 1 | 61053680 | 61054326 | 646 | 101 | 3.50E-11 | 19 | 21 | 1.6 |
| 1 | 62318806 | 62319156 | 350 | 69 | 9.60E-06 | 14 | 16 | 1.7 |
| 1 | 62319156 | 62319245 | 89 | 25 | 0.00048 | 21 | 22 | 0.9 |
| 1 | 64471379 | 64471602 | 223 | 44 | 1.40E-11 | 28 | 31 | 2.4 |
| 1 | 65002421 | 65003217 | 796 | 139 | 1.10E-10 | 19 | 20 | 0.9 |
| 1 | 75614948 | 75615199 | 251 | 50 | 0.00052 | 18 | 21 | 2.1 |
| 1 | 77281701 | 77281954 | 253 | 74 | 0.00051 | 11 | 13 | 1.7 |
| 1 | 81800630 | 81800971 | 341 | 60 | 0.0078 | 15 | 17 | 1.7 |
| 1 | 84892910 | 84893036 | 126 | 33 | 0.00031 | 19 | 21 | 2.3 |
| 1 | 89632602 | 89632825 | 223 | 33 | 1.90E-06 | 26 | 28 | 1.8 |
| 1 | 92485504 | 92486252 | 748 | 143 | 2.70E-27 | 18 | 19 | 1.8 |
| 1 | 100539331 | 100539714 | 383 | 96 | 1.30E-17 | 19 | 20 | 1.0 |
| 1 | 107140267 | 107140398 | 131 | 22 | 1.40E-05 | 15 | 17 | 1.4 |
| 1 | 107140432 | 107140583 | 151 | 26 | 8.30E-05 | 16 | 17 | 1.1 |
| 1 | 107141202 | 107141472 | 270 | 93 | 2.30E-26 | 20 | 21 | 1.0 |
| 1 | 107141539 | 107141745 | 206 | 41 | 3.20E-12 | 13 | 14 | 1.2 |
| 1 | 107964860 | 107965062 | 202 | 79 | 1.40E-09 | 14 | 15 | 1.1 |
| 1 | 108661197 | 108661384 | 187 | 41 | 2.40E-13 | 21 | 22 | 1.5 |
| 1 | 110084560 | 110084800 | 240 | 74 | 3.30E-07 | 20 | 22 | 1.3 |
| 1 | 110555399 | 110555735 | 336 | 67 | 0.0013 | 14 | 16 | 2.2 |
| 1 | 111740222 | 111740474 | 252 | 57 | 0.00011 | 17 | 19 | 2.0 |
| 1 | 114152714 | 114153089 | 375 | 86 | 5.30E-08 | 16 | 18 | 1.7 |
| 1 | 115089864 | 115089963 | 99 | 19 | 5.70E-07 | 27 | 29 | 1.9 |
| 1 | 115839128 | 115839432 | 304 | 55 | 0.00033 | 14 | 15 | 1.7 |
| 1 | 120665162 | 120665490 | 328 | 56 | 0.0064 | 6 | 6 | 0.2 |
| 1 | 120844532 | 120845010 | 478 | 61 | 3.80E-07 | 12 | 13 | 0.7 |
| 1 | 143497998 | 143498522 | 524 | 85 | 2.60E-25 | 16 | 17 | 1.3 |
| 1 | 143498522 | 143498835 | 313 | 50 | 1.90E-26 | 24 | 26 | 1.3 |
| 1 | 144412523 | 144412798 | 275 | 15 | 4.00E-05 | 19 | 21 | 1.9 |
| 1 | 145960498 | 145960963 | 465 | 73 | 0.0047 | 7 | 8 | 0.7 |
| 1 | 145961320 | 145961501 | 181 | 39 | 6.20E-05 | 13 | 14 | 0.8 |
| 1 | 145961501 | 145961588 | 87 | 23 | 6.40E-13 | 23 | 25 | 2.3 |
| 1 | 145961738 | 145962095 | 357 | 43 | 0.028 | 12 | 14 | 1.7 |
| 1 | 146369906 | 146370174 | 268 | 33 | 0.0044 | 8 | 8 | 0.2 |
| 1 | 148280716 | 148280903 | 187 | 30 | 2.10E-05 | 16 | 18 | 1.8 |
| 1 | 148522439 | 148523169 | 730 | 25 | 0.034 | 9 | 9 | 0.0 |
| 1 | 153261240 | 153261466 | 226 | 55 | 1.40E-06 | 15 | 18 | 2.4 |
| 1 | 153261639 | 153261786 | 147 | 40 | 9.90E-05 | 15 | 17 | 2.2 |
| 1 | 153679337 | 153679580 | 243 | 66 | 1.10E-11 | 18 | 19 | 1.6 |
| 1 | 154328587 | 154328957 | 370 | 103 | 0.0004 | 13 | 13 | 0.6 |
| 1 | 154501994 | 154502268 | 274 | 85 | 1.30E-11 | 13 | 14 | 1.5 |
| 1 | 154999730 | 155000549 | 819 | 74 | 0.018 | 12 | 13 | 1.1 |
| 1 | 156624656 | 156624776 | 120 | 44 | 4.30E-09 | 17 | 19 | 1.9 |
| 1 | 158180864 | 158181032 | 168 | 28 | 0.012 | 9 | 10 | 0.9 |
| 1 | 161305849 | 161306273 | 424 | 49 | 5.90E-34 | 26 | 27 | 0.8 |
| 1 | 166164945 | 166165150 | 205 | 38 | 4.70E-14 | 9 | 10 | 0.8 |
| 1 | 166165150 | 166165316 | 166 | 27 | 2.20E-07 | 14 | 16 | 2.0 |
| 1 | 166165327 | 166165656 | 329 | 52 | 3.60E-07 | 8 | 10 | 1.7 |
| 1 | 166166193 | 166166319 | 126 | 39 | 3.10E-05 | 7 | 8 | 1.1 |
| 1 | 167630238 | 167630381 | 143 | 45 | 0.035 | 15 | 16 | 0.8 |
| 1 | 171841463 | 171841642 | 179 | 43 | 5.00E-10 | 25 | 28 | 2.2 |
| 1 | 180229403 | 180229705 | 302 | 77 | 6.50E-05 | 17 | 18 | 1.0 |
| 1 | 182392056 | 182392525 | 469 | 74 | 1.20E-07 | 19 | 20 | 0.9 |
| 1 | 183417550 | 183417638 | 88 | 11 | 0.0053 | 25 | 28 | 2.3 |
| 1 | 184036684 | 184036906 | 222 | 25 | 0.0032 | 19 | 20 | 1.3 |
| 1 | 186680283 | 186680507 | 224 | 35 | 0.02 | 13 | 16 | 2.4 |
| 1 | 196608256 | 196608675 | 419 | 30 | 0.0015 | 7 | 9 | 1.8 |
| 1 | 206506867 | 206507001 | 134 | 33 | 0.0027 | 24 | 25 | 1.5 |
| 1 | 207496527 | 207496726 | 199 | 33 | 9.60E-05 | 16 | 18 | 2.1 |
| 1 | 207910959 | 207911146 | 187 | 56 | 9.90E-16 | 22 | 24 | 1.7 |
| 1 | 218164974 | 218165230 | 256 | 66 | 2.80E-09 | 18 | 20 | 1.9 |
| 1 | 218346998 | 218347590 | 592 | 40 | 0.00056 | 17 | 19 | 2.0 |
| 1 | 219928080 | 219928257 | 177 | 51 | 0.0018 | 15 | 17 | 1.3 |
| 1 | 226223579 | 226223810 | 231 | 63 | 3.40E-07 | 20 | 23 | 2.1 |
| 1 | 227542359 | 227542453 | 94 | 22 | 0.00031 | 19 | 20 | 0.4 |
| 1 | 227542470 | 227542661 | 191 | 44 | 1.70E-07 | 16 | 16 | 0.7 |
| 1 | 227788420 | 227788906 | 486 | 78 | 3.30E-06 | 16 | 18 | 1.7 |
| 1 | 227947319 | 227947566 | 247 | 40 | 0.00024 | 20 | 22 | 1.9 |
| 1 | 227948137 | 227948413 | 276 | 48 | 0.031 | 11 | 12 | 0.8 |
| 1 | 228006684 | 228006836 | 152 | 48 | 0.021 | 12 | 13 | 1.6 |
| 1 | 228037746 | 228038253 | 507 | 112 | 7.30E-25 | 18 | 20 | 2.1 |
| 1 | 228213445 | 228213716 | 271 | 64 | 0.0024 | 17 | 18 | 0.5 |
| 1 | 228275632 | 228275817 | 185 | 31 | 1.10E-06 | 15 | 17 | 1.7 |
| 1 | 228457932 | 228458585 | 653 | 95 | 0.00012 | 11 | 13 | 2.4 |
| 1 | 231161769 | 231162500 | 731 | 127 | 6.60E-20 | 16 | 18 | 2.0 |
| 1 | 232805243 | 232805701 | 458 | 89 | 2.10E-16 | 19 | 21 | 1.9 |
| 1 | 235649406 | 235649739 | 333 | 88 | 2.70E-07 | 17 | 18 | 1.1 |
| 1 | 240091659 | 240091861 | 202 | 51 | 7.90E-20 | 18 | 19 | 1.2 |
| 1 | 240091861 | 240092167 | 306 | 79 | 2.70E-47 | 24 | 26 | 1.2 |
| 1 | 241356680 | 241356805 | 125 | 52 | 5.10E-09 | 20 | 22 | 2.1 |
| 1 | 241356865 | 241357102 | 237 | 61 | 9.90E-07 | 16 | 18 | 2.0 |
| 1 | 242524602 | 242524687 | 85 | 23 | 0.00023 | 27 | 28 | 1.9 |
| 1 | 243849661 | 243850055 | 394 | 64 | 0.042 | 13 | 13 | 0.9 |
| 2 | 287689 | 287910 | 221 | 60 | 2.80E-09 | 15 | 16 | 0.6 |
| 2 | 288018 | 288391 | 373 | 123 | 2.70E-13 | 16 | 17 | 1.1 |
| 2 | 950855 | 951173 | 318 | 78 | 3.90E-20 | 16 | 19 | 2.3 |
| 2 | 1743327 | 1743533 | 206 | 49 | 0.023 | 14 | 16 | 1.6 |
| 2 | 1743913 | 1744520 | 607 | 146 | 9.50E-56 | 22 | 24 | 1.9 |
| 2 | 1744526 | 1744906 | 380 | 82 | 1.60E-32 | 19 | 21 | 2.0 |
| 2 | 5696223 | 5696403 | 180 | 35 | 2.70E-11 | 22 | 24 | 1.9 |
| 2 | 10303763 | 10304383 | 620 | 100 | 9.90E-06 | 19 | 19 | 0.6 |
| 2 | 12718341 | 12718455 | 114 | 24 | 0.0021 | 20 | 22 | 1.9 |
| 2 | 14635340 | 14635723 | 383 | 51 | 1.70E-05 | 10 | 12 | 1.3 |
| 2 | 23386232 | 23386884 | 652 | 119 | 1.20E-13 | 16 | 18 | 2.0 |
| 2 | 23386933 | 23387336 | 403 | 81 | 4.80E-05 | 13 | 14 | 1.0 |
| 2 | 24009810 | 24010054 | 244 | 56 | 3.90E-10 | 25 | 27 | 1.7 |
| 2 | 26184764 | 26184980 | 216 | 81 | 7.70E-19 | 19 | 21 | 1.8 |
| 2 | 26185012 | 26185102 | 90 | 32 | 0.032 | 20 | 22 | 2.2 |
| 2 | 26299093 | 26299281 | 188 | 19 | 4.20E-09 | 33 | 35 | 1.9 |
| 2 | 26299281 | 26299634 | 353 | 19 | 1.40E-05 | 18 | 19 | 0.8 |
| 2 | 28810420 | 28810618 | 198 | 30 | 5.20E-12 | 30 | 31 | 1.7 |
| 2 | 29115301 | 29115493 | 192 | 66 | 0.0029 | 13 | 15 | 2.1 |
| 2 | 30231147 | 30231229 | 82 | 25 | 0.00048 | 20 | 21 | 0.9 |
| 2 | 39665664 | 39666309 | 645 | 121 | 1.80E-23 | 18 | 20 | 1.4 |
| 2 | 42493831 | 42494009 | 178 | 54 | 0.043 | 16 | 18 | 1.8 |
| 2 | 42792676 | 42793306 | 630 | 129 | 5.80E-21 | 22 | 23 | 1.0 |
| 2 | 45009241 | 45009508 | 267 | 76 | 7.20E-06 | 18 | 20 | 2.2 |
| 2 | 47369344 | 47369501 | 157 | 58 | 0.023 | 16 | 18 | 1.7 |
| 2 | 47520995 | 47521504 | 509 | 111 | 4.10E-18 | 15 | 17 | 2.4 |
| 2 | 47569933 | 47570298 | 365 | 113 | 1.20E-27 | 21 | 22 | 1.8 |
| 2 | 58428808 | 58429049 | 241 | 45 | 1.10E-11 | 29 | 31 | 2.2 |
| 2 | 64609100 | 64609708 | 608 | 157 | 6.20E-13 | 20 | 21 | 1.2 |
| 2 | 69306646 | 69307030 | 384 | 68 | 0.0017 | 15 | 16 | 1.0 |
| 2 | 70767329 | 70767486 | 157 | 50 | 0.0028 | 15 | 17 | 1.7 |
| 2 | 71453569 | 71454074 | 505 | 94 | 0.00016 | 12 | 13 | 1.3 |
| 2 | 72143202 | 72143330 | 128 | 32 | 0.014 | 25 | 28 | 2.4 |
| 2 | 72147663 | 72147845 | 182 | 32 | 0.00018 | 21 | 24 | 2.2 |
| 2 | 73293194 | 73293370 | 176 | 44 | 5.50E-10 | 13 | 15 | 2.2 |
| 2 | 73293411 | 73293524 | 113 | 32 | 0.0034 | 13 | 15 | 1.6 |
| 2 | 74554716 | 74554913 | 197 | 30 | 5.20E-05 | 20 | 20 | 0.5 |
| 2 | 85133425 | 85133969 | 544 | 163 | 0.021 | 9 | 10 | 0.4 |
| 2 | 88452465 | 88452752 | 287 | 85 | 1.20E-11 | 21 | 23 | 1.8 |
| 2 | 96325323 | 96325482 | 159 | 41 | 2.10E-05 | 11 | 12 | 1.5 |
| 2 | 100104834 | 100105356 | 522 | 159 | 9.40E-07 | 10 | 11 | 0.5 |
| 2 | 100321252 | 100322597 | 1345 | 366 | 9.80E-43 | 15 | 16 | 1.0 |
| 2 | 104855581 | 104855682 | 101 | 28 | 0.015 | 19 | 20 | 1.0 |
| 2 | 104857126 | 104857231 | 105 | 19 | 0.0066 | 13 | 15 | 2.2 |
| 2 | 115161658 | 115161755 | 97 | 25 | 8.10E-05 | 27 | 29 | 1.7 |
| 2 | 115161847 | 115162037 | 190 | 61 | 8.60E-27 | 21 | 23 | 1.6 |
| 2 | 120346006 | 120346246 | 240 | 73 | 1.40E-05 | 10 | 11 | 0.5 |
| 2 | 130964139 | 130964274 | 135 | 43 | 2.80E-12 | 19 | 21 | 2.0 |
| 2 | 134718033 | 134718644 | 611 | 132 | 1.70E-45 | 25 | 26 | 1.8 |
| 2 | 136116968 | 136117285 | 317 | 56 | 0.00064 | 17 | 18 | 1.3 |
| 2 | 136765763 | 136765963 | 200 | 50 | 1.00E-08 | 17 | 19 | 2.3 |
| 2 | 143936856 | 143937709 | 853 | 214 | 8.90E-72 | 22 | 23 | 1.1 |
| 2 | 159904485 | 159904733 | 248 | 53 | 2.10E-10 | 25 | 27 | 1.9 |
| 2 | 165793657 | 165794878 | 1221 | 190 | 2.70E-07 | 13 | 15 | 2.2 |
| 2 | 167293461 | 167293640 | 179 | 36 | 3.00E-06 | 11 | 13 | 1.5 |
| 2 | 170817596 | 170817937 | 341 | 61 | 0.00038 | 15 | 17 | 1.9 |
| 2 | 174682489 | 174682744 | 255 | 76 | 7.80E-07 | 8 | 9 | 0.6 |
| 2 | 174682979 | 174683269 | 290 | 59 | 0.00079 | 15 | 16 | 1.7 |
| 2 | 184598565 | 184598717 | 152 | 56 | 2.50E-08 | 15 | 16 | 0.8 |
| 2 | 184598876 | 184599077 | 201 | 44 | 4.40E-18 | 21 | 22 | 1.5 |
| 2 | 190180263 | 190181282 | 1019 | 183 | 1.60E-17 | 20 | 22 | 1.3 |
| 2 | 212537350 | 212538481 | 1131 | 165 | 2.30E-21 | 14 | 16 | 2.4 |
| 2 | 212538482 | 212538829 | 347 | 84 | 1.10E-09 | 16 | 18 | 1.8 |
| 2 | 213284380 | 213284477 | 97 | 20 | 0.049 | 14 | 14 | 0.3 |
| 2 | 213284554 | 213284675 | 121 | 34 | 2.80E-07 | 16 | 17 | 0.2 |
| 2 | 216694380 | 216694739 | 359 | 76 | 2.10E-12 | 19 | 21 | 1.8 |
| 2 | 218981782 | 218982293 | 511 | 126 | 1.50E-07 | 14 | 16 | 1.6 |
| 2 | 219552549 | 219552668 | 119 | 44 | 2.50E-06 | 10 | 12 | 1.1 |
| 2 | 222305767 | 222306481 | 714 | 127 | 0.037 | 12 | 14 | 2.4 |
| 2 | 222318915 | 222319722 | 807 | 223 | 2.70E-12 | 14 | 16 | 1.7 |
| 2 | 222319735 | 222320186 | 451 | 110 | 5.00E-09 | 19 | 20 | 1.4 |
| 2 | 222320196 | 222320657 | 461 | 57 | 2.70E-09 | 15 | 16 | 1.5 |
| 2 | 222424640 | 222425141 | 501 | 119 | 0.00029 | 12 | 14 | 1.4 |
| 2 | 226791010 | 226792068 | 1058 | 158 | 0.0091 | 11 | 13 | 2.3 |
| 2 | 229714222 | 229714317 | 95 | 30 | 0.0083 | 16 | 18 | 1.4 |
| 2 | 231530323 | 231530407 | 84 | 30 | 0.0031 | 22 | 24 | 2.0 |
| 2 | 231925752 | 231925931 | 179 | 39 | 0.00086 | 16 | 18 | 2.2 |
| 2 | 231925958 | 231926195 | 237 | 52 | 1.20E-09 | 20 | 22 | 2.0 |
| 2 | 232420732 | 232421051 | 319 | 84 | 0.0045 | 16 | 18 | 1.3 |
| 2 | 232487590 | 232487802 | 212 | 70 | 3.40E-09 | 18 | 19 | 1.4 |
| 2 | 232487863 | 232487976 | 113 | 27 | 0.0053 | 14 | 15 | 0.9 |
| 2 | 232633628 | 232633833 | 205 | 57 | 9.30E-12 | 15 | 17 | 1.9 |
| 2 | 235669301 | 235670278 | 977 | 292 | 5.00E-24 | 15 | 18 | 2.4 |
| 2 | 237627456 | 237627630 | 174 | 49 | 2.20E-06 | 12 | 13 | 0.5 |
| 2 | 240820047 | 240820396 | 349 | 121 | 0.011 | 12 | 13 | 1.3 |
| 2 | 241217441 | 241217838 | 397 | 100 | 7.00E-08 | 14 | 15 | 1.0 |
| 3 | 3799066 | 3799295 | 229 | 43 | 0.0012 | 19 | 21 | 1.6 |
| 3 | 3799325 | 3799577 | 252 | 76 | 8.50E-14 | 19 | 20 | 0.7 |
| 3 | 3800401 | 3800959 | 558 | 65 | 6.10E-08 | 19 | 21 | 2.3 |
| 3 | 9915400 | 9915653 | 253 | 82 | 3.00E-08 | 16 | 16 | 0.6 |
| 3 | 9915653 | 9916172 | 519 | 77 | 0.00037 | 9 | 10 | 1.0 |
| 3 | 13283077 | 13283468 | 391 | 120 | 1.30E-05 | 12 | 14 | 1.3 |
| 3 | 13880597 | 13880774 | 177 | 38 | 0.0032 | 17 | 18 | 1.5 |
| 3 | 14810696 | 14810807 | 111 | 46 | 1.80E-06 | 23 | 25 | 1.9 |
| 3 | 14810885 | 14811050 | 165 | 43 | 2.20E-10 | 17 | 20 | 2.2 |
| 3 | 16884157 | 16884569 | 412 | 99 | 0.0027 | 12 | 14 | 1.7 |
| 3 | 18443710 | 18444193 | 483 | 71 | 2.70E-22 | 20 | 22 | 1.1 |
| 3 | 25427844 | 25428435 | 591 | 64 | 0.036 | 15 | 16 | 1.3 |
| 3 | 32816640 | 32816998 | 358 | 85 | 2.20E-20 | 17 | 18 | 1.1 |
| 3 | 32818165 | 32818417 | 252 | 86 | 1.30E-29 | 28 | 29 | 1.4 |
| 3 | 32818439 | 32818649 | 210 | 83 | 3.30E-23 | 25 | 27 | 2.4 |
| 3 | 33218693 | 33218963 | 270 | 72 | 2.30E-15 | 22 | 24 | 1.8 |
| 3 | 38029481 | 38029649 | 168 | 45 | 2.00E-05 | 16 | 16 | 0.4 |
| 3 | 42773110 | 42773612 | 502 | 73 | 0.028 | 13 | 13 | 0.6 |
| 3 | 42905935 | 42906179 | 244 | 42 | 7.80E-05 | 19 | 21 | 2.3 |
| 3 | 43995080 | 43995762 | 682 | 136 | 2.10E-25 | 18 | 20 | 1.7 |
| 3 | 43996817 | 43997085 | 268 | 41 | 4.00E-05 | 20 | 22 | 1.9 |
| 3 | 43999281 | 43999460 | 179 | 54 | 9.40E-06 | 14 | 17 | 2.2 |
| 3 | 44555240 | 44555339 | 99 | 34 | 1.30E-09 | 18 | 19 | 1.0 |
| 3 | 44555339 | 44555611 | 272 | 45 | 8.80E-07 | 11 | 12 | 1.1 |
| 3 | 44861797 | 44862093 | 296 | 86 | 1.20E-05 | 16 | 16 | 0.3 |
| 3 | 46882439 | 46882655 | 216 | 57 | 5.40E-08 | 19 | 21 | 2.0 |
| 3 | 48989512 | 48990569 | 1057 | 208 | 0.012 | 11 | 11 | 0.4 |
| 3 | 48990592 | 48990758 | 166 | 40 | 0.0003 | 19 | 20 | 1.2 |
| 3 | 49869791 | 49869921 | 130 | 42 | 0.0043 | 20 | 22 | 1.7 |
| 3 | 54122206 | 54122452 | 246 | 78 | 0.0062 | 15 | 16 | 0.5 |
| 3 | 62874598 | 62874920 | 322 | 71 | 2.40E-10 | 13 | 15 | 2.0 |
| 3 | 71581307 | 71581668 | 361 | 78 | 4.10E-13 | 27 | 28 | 1.1 |
| 3 | 71581668 | 71582164 | 496 | 54 | 0.00012 | 19 | 20 | 0.6 |
| 3 | 71582227 | 71582894 | 667 | 162 | 4.60E-17 | 20 | 20 | 0.3 |
| 3 | 71753381 | 71753653 | 272 | 80 | 3.10E-20 | 21 | 23 | 1.9 |
| 3 | 71753654 | 71754547 | 893 | 264 | 1.20E-39 | 16 | 18 | 2.0 |
| 3 | 72738803 | 72739110 | 307 | 66 | 2.60E-11 | 27 | 29 | 2.4 |
| 3 | 96813516 | 96813706 | 190 | 53 | 0.0018 | 13 | 15 | 2.0 |
| 3 | 96814073 | 96814323 | 250 | 50 | 0.0024 | 15 | 16 | 1.7 |
| 3 | 96814325 | 96814433 | 108 | 19 | 0.012 | 21 | 23 | 1.6 |
| 3 | 101779223 | 101779493 | 270 | 62 | 0.0022 | 13 | 14 | 0.8 |
| 3 | 112333046 | 112333503 | 457 | 65 | 1.00E-09 | 17 | 18 | 1.3 |
| 3 | 120450857 | 120451143 | 286 | 55 | 7.70E-12 | 16 | 18 | 1.5 |
| 3 | 127822479 | 127822792 | 313 | 45 | 0.011 | 12 | 12 | 0.3 |
| 3 | 129001340 | 129001646 | 306 | 64 | 8.40E-10 | 15 | 16 | 1.0 |
| 3 | 129001648 | 129002087 | 439 | 113 | 8.10E-40 | 22 | 23 | 1.8 |
| 3 | 129002142 | 129002340 | 198 | 62 | 1.70E-16 | 20 | 22 | 1.5 |
| 3 | 129627293 | 129628296 | 1003 | 88 | 0.00091 | 14 | 15 | 1.4 |
| 3 | 133927488 | 133927610 | 122 | 40 | 8.80E-05 | 20 | 22 | 2.2 |
| 3 | 134796108 | 134796247 | 139 | 31 | 4.60E-05 | 12 | 14 | 1.7 |
| 3 | 136818939 | 136819411 | 472 | 111 | 2.80E-08 | 15 | 16 | 0.8 |
| 3 | 138434449 | 138435138 | 689 | 140 | 3.60E-11 | 12 | 13 | 1.3 |
| 3 | 138435168 | 138435436 | 268 | 53 | 2.60E-16 | 22 | 24 | 1.9 |
| 3 | 138946918 | 138947089 | 171 | 25 | 0.012 | 16 | 17 | 1.5 |
| 3 | 139539440 | 139539674 | 234 | 50 | 7.20E-10 | 12 | 13 | 1.0 |
| 3 | 139935158 | 139935244 | 86 | 32 | 6.70E-06 | 15 | 16 | 0.9 |
| 3 | 139935245 | 139935345 | 100 | 36 | 4.90E-06 | 17 | 18 | 1.0 |
| 3 | 141051045 | 141051178 | 133 | 35 | 2.10E-06 | 19 | 20 | 1.7 |
| 3 | 141051556 | 141051739 | 183 | 51 | 6.90E-16 | 16 | 17 | 1.7 |
| 3 | 141052771 | 141053120 | 349 | 48 | 1.10E-06 | 12 | 14 | 2.0 |
| 3 | 142963267 | 142964108 | 841 | 243 | 1.20E-20 | 13 | 14 | 0.8 |
| 3 | 143119700 | 143120533 | 833 | 213 | 6.40E-47 | 18 | 19 | 0.9 |
| 3 | 143120533 | 143120896 | 363 | 85 | 9.40E-17 | 18 | 19 | 1.3 |
| 3 | 151085561 | 151085702 | 141 | 48 | 6.80E-12 | 22 | 22 | 0.4 |
| 3 | 161105244 | 161105420 | 176 | 54 | 0.0016 | 10 | 11 | 1.0 |
| 3 | 169661298 | 169661905 | 607 | 65 | 1.20E-05 | 16 | 18 | 2.0 |
| 3 | 169764864 | 169765264 | 400 | 75 | 0.00056 | 11 | 11 | 0.4 |
| 3 | 169812238 | 169812435 | 197 | 43 | 1.20E-07 | 19 | 20 | 1.0 |
| 3 | 169812435 | 169812853 | 418 | 58 | 1.30E-10 | 19 | 20 | 1.5 |
| 3 | 183254409 | 183254687 | 278 | 73 | 3.90E-07 | 13 | 14 | 1.6 |
| 3 | 183825198 | 183825702 | 504 | 164 | 8.10E-24 | 19 | 21 | 1.3 |
| 3 | 187139608 | 187139845 | 237 | 30 | 0.042 | 11 | 12 | 0.7 |
| 3 | 187737510 | 187738394 | 884 | 99 | 2.30E-07 | 19 | 20 | 1.1 |
| 3 | 192408696 | 192408841 | 145 | 18 | 0.0083 | 21 | 22 | 1.1 |
| 3 | 192409285 | 192409673 | 388 | 91 | 4.00E-22 | 20 | 21 | 1.6 |
| 3 | 192409757 | 192409911 | 154 | 40 | 3.10E-05 | 16 | 18 | 2.1 |
| 3 | 194058258 | 194058536 | 278 | 49 | 1.60E-14 | 25 | 26 | 0.5 |
| 3 | 194686699 | 194686989 | 290 | 75 | 0.0096 | 14 | 15 | 0.7 |
| 4 | 663904 | 664234 | 330 | 62 | 3.80E-09 | 17 | 19 | 1.8 |
| 4 | 3765489 | 3765852 | 363 | 106 | 0.018 | 12 | 14 | 1.1 |
| 4 | 3766050 | 3766585 | 535 | 190 | 1.00E-09 | 12 | 14 | 1.0 |
| 4 | 4386190 | 4386454 | 264 | 37 | 0.0028 | 18 | 19 | 1.7 |
| 4 | 4386877 | 4387022 | 145 | 39 | 1.90E-05 | 22 | 23 | 1.0 |
| 4 | 4387022 | 4387471 | 449 | 115 | 1.20E-16 | 17 | 18 | 1.6 |
| 4 | 5888104 | 5888385 | 281 | 67 | 2.50E-10 | 17 | 19 | 2.0 |
| 4 | 6472531 | 6472648 | 117 | 52 | 0.028 | 15 | 16 | 1.0 |
| 4 | 13544166 | 13544503 | 337 | 91 | 4.60E-15 | 20 | 22 | 2.3 |
| 4 | 13547276 | 13547495 | 219 | 61 | 0.00045 | 15 | 17 | 1.9 |
| 4 | 13547498 | 13547998 | 500 | 108 | 0.0076 | 13 | 15 | 2.3 |
| 4 | 16083489 | 16083714 | 225 | 35 | 0.047 | 24 | 27 | 2.4 |
| 4 | 37244561 | 37244677 | 116 | 24 | 4.40E-07 | 19 | 22 | 2.3 |
| 4 | 41257218 | 41257529 | 311 | 84 | 1.30E-09 | 15 | 17 | 2.1 |
| 4 | 42151994 | 42152340 | 346 | 83 | 1.90E-26 | 23 | 24 | 1.7 |
| 4 | 42398199 | 42398465 | 266 | 66 | 9.90E-10 | 15 | 17 | 1.3 |
| 4 | 52051286 | 52051378 | 92 | 39 | 3.80E-05 | 21 | 24 | 2.4 |
| 4 | 52862112 | 52862627 | 515 | 144 | 1.50E-22 | 19 | 21 | 1.4 |
| 4 | 72569113 | 72569348 | 235 | 29 | 0.041 | 18 | 18 | 0.8 |
| 4 | 75939764 | 75940240 | 476 | 69 | 0.011 | 18 | 19 | 1.3 |
| 4 | 80202316 | 80202435 | 119 | 38 | 0.0052 | 21 | 22 | 1.0 |
| 4 | 80202439 | 80203160 | 721 | 193 | 2.20E-30 | 16 | 18 | 2.2 |
| 4 | 86594226 | 86594493 | 267 | 55 | 0.0071 | 15 | 16 | 1.3 |
| 4 | 90127925 | 90128227 | 302 | 90 | 5.90E-05 | 13 | 14 | 1.3 |
| 4 | 92303934 | 92304014 | 80 | 22 | 0.01 | 22 | 23 | 1.5 |
| 4 | 94758076 | 94758271 | 195 | 71 | 2.20E-06 | 12 | 13 | 1.9 |
| 4 | 100189989 | 100190486 | 497 | 81 | 7.80E-09 | 16 | 17 | 1.6 |
| 4 | 114599543 | 114599682 | 139 | 31 | 2.50E-10 | 32 | 35 | 2.4 |
| 4 | 120922368 | 120922501 | 133 | 43 | 0.00018 | 16 | 17 | 1.2 |
| 4 | 121072188 | 121073082 | 894 | 147 | 1.50E-09 | 11 | 13 | 1.5 |
| 4 | 123398727 | 123400185 | 1458 | 141 | 2.10E-11 | 19 | 21 | 1.2 |
| 4 | 148443548 | 148444556 | 1008 | 172 | 0.022 | 11 | 11 | 0.4 |
| 4 | 150079136 | 150079463 | 327 | 53 | 0.0015 | 17 | 19 | 1.9 |
| 4 | 153153180 | 153153893 | 713 | 144 | 1.50E-06 | 13 | 14 | 0.7 |
| 4 | 153222901 | 153223610 | 709 | 107 | 3.40E-16 | 16 | 19 | 2.4 |
| 4 | 155667145 | 155667442 | 297 | 57 | 1.70E-05 | 16 | 18 | 2.4 |
| 4 | 155759120 | 155759417 | 297 | 62 | 1.70E-14 | 21 | 23 | 2.1 |
| 4 | 155759732 | 155759982 | 250 | 41 | 9.90E-07 | 19 | 21 | 1.5 |
| 4 | 165873421 | 165873747 | 326 | 80 | 4.90E-05 | 11 | 12 | 1.1 |
| 4 | 180058275 | 180058550 | 275 | 69 | 1.20E-08 | 14 | 15 | 1.0 |
| 4 | 183797801 | 183798132 | 331 | 83 | 1.20E-09 | 13 | 15 | 1.3 |
| 4 | 183905240 | 183905858 | 618 | 108 | 0.00031 | 13 | 15 | 2.1 |
| 4 | 183905888 | 183906274 | 386 | 89 | 0.0037 | 12 | 14 | 1.3 |
| 4 | 185128573 | 185128730 | 157 | 45 | 0.00011 | 16 | 17 | 1.2 |
| 4 | 186104473 | 186104765 | 292 | 49 | 2.90E-05 | 19 | 21 | 1.2 |
| 4 | 186104813 | 186105125 | 312 | 78 | 6.60E-05 | 16 | 17 | 1.2 |
| 4 | 186144646 | 186144936 | 290 | 45 | 0.00026 | 11 | 11 | 0.0 |
| 5 | 321080 | 321386 | 306 | 54 | 3.40E-06 | 16 | 18 | 1.6 |
| 5 | 528571 | 528669 | 98 | 32 | 0.00015 | 13 | 14 | 1.0 |
| 5 | 691213 | 691310 | 97 | 15 | 1.80E-05 | 11 | 11 | 0.3 |
| 5 | 2751623 | 2751743 | 120 | 29 | 1.70E-09 | 22 | 24 | 2.1 |
| 5 | 6448601 | 6448806 | 205 | 52 | 1.50E-09 | 12 | 14 | 1.9 |
| 5 | 6448810 | 6449275 | 465 | 101 | 7.70E-10 | 15 | 17 | 1.7 |
| 5 | 10333738 | 10334007 | 269 | 72 | 0.011 | 12 | 14 | 2.0 |
| 5 | 10564488 | 10565033 | 545 | 132 | 8.60E-43 | 22 | 24 | 1.3 |
| 5 | 11904147 | 11904324 | 177 | 75 | 4.50E-14 | 16 | 18 | 2.0 |
| 5 | 11904350 | 11904530 | 180 | 57 | 6.80E-07 | 17 | 18 | 1.6 |
| 5 | 15500085 | 15500481 | 396 | 114 | 3.90E-18 | 9 | 11 | 2.2 |
| 5 | 17218420 | 17219021 | 601 | 86 | 1.50E-29 | 23 | 24 | 1.5 |
| 5 | 32711774 | 32711896 | 122 | 37 | 3.40E-17 | 20 | 22 | 1.1 |
| 5 | 38556774 | 38557042 | 268 | 75 | 5.90E-06 | 8 | 8 | 0.4 |
| 5 | 59039751 | 59040015 | 264 | 76 | 0.0096 | 20 | 21 | 1.4 |
| 5 | 65924516 | 65925629 | 1113 | 137 | 0.0018 | 13 | 14 | 1.4 |
| 5 | 72108730 | 72109001 | 271 | 45 | 4.30E-06 | 17 | 18 | 0.7 |
| 5 | 76082208 | 76082459 | 251 | 36 | 0.013 | 12 | 14 | 2.1 |
| 5 | 76084804 | 76084886 | 82 | 26 | 0.035 | 16 | 17 | 1.3 |
| 5 | 77210760 | 77210841 | 81 | 33 | 0.0044 | 14 | 15 | 0.3 |
| 5 | 77210862 | 77211019 | 157 | 48 | 7.30E-09 | 20 | 21 | 1.0 |
| 5 | 77211126 | 77211226 | 100 | 26 | 8.00E-05 | 21 | 23 | 2.1 |
| 5 | 77852696 | 77852868 | 172 | 37 | 1.00E-08 | 18 | 20 | 1.8 |
| 5 | 80569988 | 80570201 | 213 | 46 | 1.10E-06 | 17 | 19 | 1.9 |
| 5 | 81394212 | 81394409 | 197 | 40 | 1.10E-07 | 22 | 23 | 1.5 |
| 5 | 88889586 | 88890171 | 585 | 43 | 1.70E-15 | 24 | 26 | 1.8 |
| 5 | 95620306 | 95620443 | 137 | 20 | 0.011 | 25 | 27 | 1.4 |
| 5 | 95620470 | 95620863 | 393 | 110 | 2.50E-32 | 19 | 19 | 0.7 |
| 5 | 102295841 | 102296798 | 957 | 115 | 1.80E-31 | 25 | 27 | 1.3 |
| 5 | 111224039 | 111224585 | 546 | 136 | 1.20E-06 | 11 | 11 | 0.7 |
| 5 | 114362273 | 114363031 | 758 | 169 | 7.50E-27 | 16 | 19 | 2.4 |
| 5 | 115601623 | 115602763 | 1140 | 148 | 2.80E-14 | 20 | 22 | 1.3 |
| 5 | 116573868 | 116574357 | 489 | 99 | 0.0064 | 13 | 15 | 1.8 |
| 5 | 119355528 | 119356215 | 687 | 95 | 3.70E-08 | 16 | 19 | 2.3 |
| 5 | 122077493 | 122077716 | 223 | 64 | 1.70E-06 | 14 | 16 | 1.8 |
| 5 | 123090100 | 123090229 | 129 | 38 | 7.60E-05 | 14 | 15 | 0.4 |
| 5 | 127229480 | 127229813 | 333 | 61 | 0.013 | 13 | 14 | 0.8 |
| 5 | 129905350 | 129905857 | 507 | 72 | 0.004 | 11 | 12 | 1.4 |
| 5 | 132257582 | 132257685 | 103 | 26 | 1.10E-09 | 22 | 23 | 0.8 |
| 5 | 132656177 | 132656588 | 411 | 110 | 2.10E-32 | 17 | 19 | 1.7 |
| 5 | 132747181 | 132747366 | 185 | 48 | 6.10E-08 | 17 | 19 | 1.4 |
| 5 | 132747463 | 132747618 | 155 | 38 | 4.50E-07 | 17 | 19 | 2.0 |
| 5 | 133611579 | 133611818 | 239 | 47 | 0.0042 | 17 | 19 | 2.0 |
| 5 | 135033686 | 135034047 | 361 | 100 | 0.0019 | 12 | 13 | 1.2 |
| 5 | 135543875 | 135544105 | 230 | 56 | 1.00E-10 | 14 | 15 | 1.6 |
| 5 | 135578838 | 135578981 | 143 | 38 | 1.00E-14 | 17 | 20 | 2.3 |
| 5 | 136192319 | 136193493 | 1174 | 217 | 1.00E-09 | 12 | 14 | 2.3 |
| 5 | 139637344 | 139637987 | 643 | 120 | 3.20E-10 | 20 | 21 | 1.1 |
| 5 | 139650353 | 139650836 | 483 | 80 | 0.0056 | 20 | 21 | 1.5 |
| 5 | 139848103 | 139848288 | 185 | 85 | 2.70E-18 | 18 | 20 | 1.6 |
| 5 | 139848288 | 139848471 | 183 | 57 | 5.20E-12 | 16 | 17 | 1.1 |
| 5 | 139904030 | 139904316 | 286 | 81 | 2.20E-13 | 14 | 15 | 0.7 |
| 5 | 140632048 | 140633185 | 1137 | 171 | 5.00E-16 | 19 | 21 | 1.9 |
| 5 | 141476357 | 141476826 | 469 | 72 | 7.70E-14 | 18 | 20 | 2.2 |
| 5 | 141876304 | 141876926 | 622 | 88 | 0.0073 | 13 | 14 | 1.2 |
| 5 | 157459961 | 157460544 | 583 | 104 | 4.20E-16 | 17 | 19 | 2.0 |
| 5 | 168529407 | 168529500 | 93 | 23 | 2.00E-15 | 33 | 35 | 1.8 |
| 5 | 169637356 | 169637472 | 116 | 34 | 4.80E-17 | 31 | 34 | 2.2 |
| 5 | 171419083 | 171420488 | 1405 | 325 | 5.80E-08 | 11 | 12 | 1.1 |
| 5 | 171420576 | 171421035 | 459 | 85 | 3.50E-13 | 19 | 22 | 2.4 |
| 5 | 176365291 | 176365863 | 572 | 130 | 3.50E-06 | 11 | 12 | 0.3 |
| 5 | 176809735 | 176809839 | 104 | 31 | 1.00E-08 | 21 | 23 | 2.2 |
| 5 | 177455603 | 177456153 | 550 | 130 | 2.70E-07 | 15 | 15 | 0.7 |
| 5 | 177944021 | 177944790 | 769 | 98 | 2.90E-17 | 18 | 19 | 0.9 |
| 5 | 178006855 | 178006952 | 97 | 23 | 3.00E-17 | 33 | 35 | 1.8 |
| 5 | 178589724 | 178590595 | 871 | 232 | 1.40E-70 | 21 | 23 | 2.1 |
| 5 | 179023505 | 179023816 | 311 | 55 | 9.20E-15 | 19 | 19 | 0.8 |
| 5 | 179023820 | 179024005 | 185 | 50 | 2.50E-09 | 12 | 13 | 0.5 |
| 5 | 179060001 | 179060274 | 273 | 32 | 0.0064 | 13 | 15 | 2.4 |
| 5 | 179345219 | 179345385 | 166 | 56 | 6.60E-06 | 17 | 18 | 1.0 |
| 5 | 180209132 | 180209419 | 287 | 90 | 0.025 | 12 | 14 | 1.2 |
| 5 | 180353013 | 180353296 | 283 | 81 | 2.40E-17 | 20 | 22 | 1.7 |
| 5 | 180353540 | 180354031 | 491 | 102 | 1.10E-26 | 18 | 19 | 1.0 |
| 5 | 180590875 | 180590972 | 97 | 25 | 0.00028 | 15 | 17 | 2.4 |
| 6 | 1390092 | 1390342 | 250 | 71 | 5.60E-11 | 12 | 14 | 2.1 |
| 6 | 4775251 | 4775646 | 395 | 74 | 1.70E-14 | 21 | 23 | 1.9 |
| 6 | 7726563 | 7727278 | 715 | 168 | 5.30E-11 | 15 | 17 | 1.3 |
| 6 | 7727347 | 7727586 | 239 | 60 | 2.30E-09 | 18 | 20 | 1.8 |
| 6 | 17280884 | 17280975 | 91 | 33 | 1.40E-09 | 20 | 23 | 2.3 |
| 6 | 17281106 | 17281785 | 679 | 158 | 0.0029 | 10 | 12 | 2.0 |
| 6 | 18277258 | 18277656 | 398 | 77 | 2.10E-18 | 16 | 18 | 2.1 |
| 6 | 21587015 | 21587456 | 441 | 51 | 2.10E-11 | 26 | 28 | 2.3 |
| 6 | 21596333 | 21597330 | 997 | 73 | 0.024 | 10 | 10 | 0.2 |
| 6 | 26043801 | 26044538 | 737 | 72 | 0.00042 | 7 | 9 | 1.5 |
| 6 | 26044558 | 26045105 | 547 | 40 | 0.0088 | 11 | 12 | 0.8 |
| 6 | 26045460 | 26045804 | 344 | 64 | 0.029 | 7 | 9 | 1.8 |
| 6 | 29926698 | 29926903 | 205 | 53 | 0.0063 | 19 | 20 | 1.2 |
| 6 | 30006592 | 30006736 | 144 | 24 | 0.0045 | 16 | 18 | 1.5 |
| 6 | 30883861 | 30884985 | 1124 | 182 | 1.60E-05 | 10 | 11 | 1.1 |
| 6 | 41638214 | 41638355 | 141 | 37 | 0.023 | 15 | 16 | 1.3 |
| 6 | 46735187 | 46735281 | 94 | 29 | 0.01 | 22 | 24 | 2.3 |
| 6 | 62286131 | 62286489 | 358 | 56 | 1.70E-11 | 18 | 20 | 2.4 |
| 6 | 70956751 | 70956858 | 107 | 26 | 0.022 | 20 | 20 | 0.9 |
| 6 | 73451654 | 73452012 | 358 | 76 | 6.60E-16 | 15 | 17 | 1.3 |
| 6 | 73452021 | 73452167 | 146 | 28 | 7.50E-06 | 21 | 22 | 0.7 |
| 6 | 79947003 | 79947589 | 586 | 131 | 2.00E-29 | 18 | 20 | 1.8 |
| 6 | 83709093 | 83709267 | 174 | 43 | 0.023 | 16 | 18 | 2.1 |
| 6 | 83853377 | 83853523 | 146 | 46 | 3.30E-06 | 20 | 22 | 2.2 |
| 6 | 87151923 | 87152640 | 717 | 141 | 2.90E-08 | 16 | 19 | 2.2 |
| 6 | 88166693 | 88166855 | 162 | 44 | 0.00051 | 19 | 20 | 0.6 |
| 6 | 88166868 | 88167209 | 341 | 102 | 3.10E-28 | 28 | 29 | 1.4 |
| 6 | 90611112 | 90611353 | 241 | 51 | 0.001 | 16 | 17 | 1.3 |
| 6 | 99613765 | 99614094 | 329 | 75 | 1.00E-05 | 13 | 15 | 1.8 |
| 6 | 106510119 | 106510681 | 562 | 72 | 0.02 | 14 | 17 | 2.3 |
| 6 | 110357792 | 110358006 | 214 | 50 | 8.20E-19 | 21 | 23 | 1.7 |
| 6 | 110358166 | 110358302 | 136 | 40 | 8.80E-20 | 26 | 27 | 1.2 |
| 6 | 110358308 | 110358405 | 97 | 20 | 4.10E-06 | 18 | 19 | 0.8 |
| 6 | 113856304 | 113856763 | 459 | 39 | 0.005 | 13 | 14 | 1.8 |
| 6 | 117265544 | 117265639 | 95 | 22 | 0.017 | 18 | 20 | 2.0 |
| 6 | 117907243 | 117907564 | 321 | 91 | 1.30E-28 | 27 | 28 | 1.5 |
| 6 | 117907566 | 117907909 | 343 | 94 | 2.70E-26 | 21 | 22 | 1.0 |
| 6 | 117908011 | 117908120 | 109 | 26 | 8.30E-05 | 24 | 24 | 0.7 |
| 6 | 123803254 | 123803380 | 126 | 36 | 2.80E-11 | 21 | 22 | 0.9 |
| 6 | 123803384 | 123803605 | 221 | 46 | 0.00013 | 14 | 15 | 0.9 |
| 6 | 123803686 | 123803860 | 174 | 69 | 1.70E-14 | 18 | 19 | 1.2 |
| 6 | 123803900 | 123804028 | 128 | 47 | 6.10E-15 | 15 | 16 | 0.6 |
| 6 | 125363337 | 125363668 | 331 | 59 | 6.60E-08 | 14 | 16 | 1.5 |
| 6 | 127119634 | 127119912 | 278 | 72 | 2.10E-13 | 17 | 19 | 1.8 |
| 6 | 130365365 | 130365447 | 82 | 21 | 0.014 | 25 | 28 | 2.4 |
| 6 | 133241054 | 133241197 | 143 | 34 | 5.50E-10 | 25 | 27 | 1.4 |
| 6 | 136791195 | 136791735 | 540 | 65 | 0.0012 | 16 | 16 | 0.8 |
| 6 | 136922168 | 136922361 | 193 | 48 | 0.00012 | 21 | 23 | 1.5 |
| 6 | 142926519 | 142927523 | 1004 | 163 | 2.90E-15 | 18 | 18 | 0.5 |
| 6 | 148747976 | 148748522 | 546 | 71 | 5.10E-13 | 19 | 22 | 2.0 |
| 6 | 151240542 | 151240996 | 454 | 97 | 2.50E-10 | 11 | 12 | 1.4 |
| 6 | 158537197 | 158537290 | 93 | 41 | 0.0016 | 21 | 22 | 1.3 |
| 6 | 159169382 | 159169606 | 224 | 71 | 2.60E-08 | 17 | 19 | 2.2 |
| 6 | 159169610 | 159169761 | 151 | 54 | 1.10E-22 | 24 | 26 | 2.0 |
| 6 | 165660972 | 165661276 | 304 | 48 | 3.80E-08 | 17 | 20 | 2.4 |
| 6 | 165663028 | 165663225 | 197 | 60 | 0.00015 | 18 | 20 | 1.5 |
| 6 | 165663233 | 165663864 | 631 | 102 | 4.40E-08 | 14 | 16 | 1.6 |
| 6 | 166168571 | 166168765 | 194 | 28 | 0.0057 | 20 | 21 | 1.0 |
| 6 | 170612401 | 170612687 | 286 | 23 | 1.60E-06 | 20 | 22 | 2.2 |
| 7 | 712464 | 712572 | 108 | 42 | 3.60E-15 | 30 | 33 | 2.4 |
| 7 | 954547 | 954684 | 137 | 56 | 0.015 | 16 | 17 | 0.6 |
| 7 | 3301059 | 3301330 | 271 | 103 | 5.10E-17 | 19 | 19 | 0.5 |
| 7 | 3301390 | 3301975 | 585 | 221 | 3.10E-60 | 21 | 22 | 0.9 |
| 7 | 4883630 | 4883788 | 158 | 39 | 0.00038 | 17 | 19 | 1.8 |
| 7 | 4959229 | 4959705 | 476 | 32 | 0.0027 | 14 | 15 | 1.0 |
| 7 | 5427297 | 5428175 | 878 | 153 | 9.90E-11 | 14 | 15 | 1.3 |
| 7 | 5592941 | 5593662 | 721 | 198 | 1.30E-40 | 20 | 22 | 2.2 |
| 7 | 5593688 | 5593909 | 221 | 51 | 0.0045 | 13 | 15 | 1.9 |
| 7 | 6531133 | 6531239 | 106 | 34 | 0.0056 | 16 | 17 | 1.3 |
| 7 | 6536324 | 6536612 | 288 | 51 | 4.20E-07 | 17 | 19 | 2.1 |
| 7 | 6615327 | 6615415 | 88 | 32 | 2.00E-08 | 23 | 25 | 2.1 |
| 7 | 6854918 | 6855317 | 399 | 69 | 4.80E-06 | 18 | 20 | 2.4 |
| 7 | 18086323 | 18086480 | 157 | 39 | 1.90E-07 | 20 | 22 | 2.1 |
| 7 | 18086568 | 18086705 | 137 | 48 | 4.60E-09 | 19 | 19 | 0.4 |
| 7 | 18086765 | 18087043 | 278 | 87 | 1.10E-14 | 20 | 22 | 1.4 |
| 7 | 20331421 | 20332017 | 596 | 112 | 0.00012 | 11 | 12 | 1.6 |
| 7 | 23469295 | 23469540 | 245 | 53 | 8.30E-09 | 17 | 19 | 2.1 |
| 7 | 27173484 | 27173646 | 162 | 57 | 8.20E-13 | 17 | 19 | 2.1 |
| 7 | 27200236 | 27200378 | 142 | 49 | 0.033 | 11 | 12 | 1.4 |
| 7 | 28956447 | 28956990 | 543 | 98 | 3.20E-10 | 16 | 18 | 2.1 |
| 7 | 29989053 | 29989188 | 135 | 52 | 2.10E-08 | 13 | 13 | 0.7 |
| 7 | 30682318 | 30682517 | 199 | 50 | 1.20E-14 | 18 | 19 | 1.4 |
| 7 | 33904132 | 33904408 | 276 | 47 | 0.0089 | 17 | 20 | 2.2 |
| 7 | 35186491 | 35187078 | 587 | 82 | 1.70E-07 | 10 | 11 | 1.1 |
| 7 | 37447801 | 37448128 | 327 | 64 | 5.70E-10 | 13 | 14 | 0.6 |
| 7 | 38631217 | 38631409 | 192 | 46 | 2.90E-07 | 15 | 17 | 2.3 |
| 7 | 43113290 | 43113690 | 400 | 64 | 0.0039 | 13 | 15 | 1.9 |
| 7 | 44104426 | 44104746 | 320 | 47 | 0.0014 | 12 | 13 | 1.5 |
| 7 | 44309836 | 44310071 | 235 | 67 | 7.20E-09 | 17 | 19 | 2.1 |
| 7 | 45574445 | 45574771 | 326 | 110 | 1.10E-24 | 19 | 19 | 0.6 |
| 7 | 47581224 | 47582218 | 994 | 199 | 9.20E-06 | 10 | 11 | 0.9 |
| 7 | 49773412 | 49773530 | 118 | 34 | 9.20E-24 | 28 | 30 | 2.2 |
| 7 | 49773584 | 49773689 | 105 | 36 | 1.70E-37 | 35 | 37 | 2.0 |
| 7 | 49775701 | 49775808 | 107 | 19 | 0.0026 | 11 | 13 | 1.9 |
| 7 | 50303893 | 50303992 | 99 | 46 | 5.50E-24 | 30 | 31 | 0.9 |
| 7 | 50304077 | 50304498 | 421 | 133 | 1.90E-74 | 28 | 29 | 0.8 |
| 7 | 50304540 | 50304761 | 221 | 50 | 1.30E-26 | 33 | 33 | 0.7 |
| 7 | 64947463 | 64947871 | 408 | 74 | 2.60E-07 | 14 | 16 | 2.2 |
| 7 | 69598274 | 69598420 | 146 | 63 | 0.019 | 12 | 13 | 0.5 |
| 7 | 69599513 | 69599737 | 224 | 56 | 4.30E-09 | 16 | 16 | 0.8 |
| 7 | 70695600 | 70696208 | 608 | 45 | 0.0055 | 15 | 17 | 2.3 |
| 7 | 76267192 | 76267509 | 317 | 94 | 2.00E-10 | 16 | 18 | 2.3 |
| 7 | 79452350 | 79452722 | 372 | 42 | 6.50E-06 | 13 | 15 | 1.6 |
| 7 | 79453068 | 79453848 | 780 | 79 | 0.045 | 6 | 8 | 1.5 |
| 7 | 87600164 | 87600540 | 376 | 70 | 2.30E-11 | 20 | 22 | 1.9 |
| 7 | 92179121 | 92179430 | 309 | 54 | 0.015 | 11 | 13 | 1.6 |
| 7 | 97006034 | 97006232 | 198 | 47 | 0.00064 | 8 | 9 | 1.1 |
| 7 | 98869829 | 98870075 | 246 | 81 | 1.20E-18 | 20 | 22 | 2.0 |
| 7 | 99580314 | 99580459 | 145 | 30 | 0.033 | 18 | 18 | 0.8 |
| 7 | 100675722 | 100676032 | 310 | 46 | 4.60E-05 | 19 | 19 | 0.3 |
| 7 | 100676049 | 100676222 | 173 | 36 | 0.0044 | 16 | 17 | 0.7 |
| 7 | 100720860 | 100721290 | 430 | 57 | 3.90E-07 | 12 | 14 | 2.3 |
| 7 | 101180119 | 101180369 | 250 | 72 | 3.40E-08 | 15 | 17 | 2.2 |
| 7 | 102671533 | 102671766 | 233 | 43 | 1.50E-11 | 7 | 7 | 0.1 |
| 7 | 104328715 | 104328890 | 175 | 40 | 2.80E-08 | 19 | 21 | 2.0 |
| 7 | 104328890 | 104329116 | 226 | 35 | 2.50E-05 | 13 | 16 | 2.4 |
| 7 | 104983827 | 104984443 | 616 | 94 | 0.00014 | 5 | 6 | 0.7 |
| 7 | 107661154 | 107661425 | 271 | 34 | 0.02 | 13 | 14 | 1.3 |
| 7 | 107661516 | 107661674 | 158 | 31 | 0.017 | 17 | 18 | 0.9 |
| 7 | 113086480 | 113086602 | 122 | 39 | 0.0039 | 16 | 18 | 2.3 |
| 7 | 117323078 | 117323165 | 87 | 34 | 3.00E-08 | 16 | 18 | 2.1 |
| 7 | 117873172 | 117873386 | 214 | 65 | 0.031 | 8 | 9 | 1.2 |
| 7 | 117873390 | 117873613 | 223 | 78 | 4.00E-11 | 11 | 12 | 0.9 |
| 7 | 120273234 | 120273390 | 156 | 40 | 1.20E-06 | 18 | 21 | 2.1 |
| 7 | 127251864 | 127251952 | 88 | 22 | 2.60E-09 | 20 | 21 | 1.3 |
| 7 | 128104048 | 128104341 | 293 | 67 | 1.10E-24 | 28 | 30 | 2.1 |
| 7 | 129779226 | 129779697 | 471 | 108 | 1.20E-05 | 14 | 16 | 1.6 |
| 7 | 130273387 | 130273493 | 106 | 12 | 0.012 | 27 | 27 | 0.6 |
| 7 | 134458372 | 134458656 | 284 | 40 | 1.30E-19 | 23 | 24 | 1.2 |
| 7 | 134458675 | 134458821 | 146 | 22 | 0.0058 | 9 | 10 | 1.0 |
| 7 | 134458962 | 134459112 | 150 | 27 | 0.00023 | 14 | 15 | 0.9 |
| 7 | 134459112 | 134459202 | 90 | 24 | 6.80E-07 | 20 | 21 | 1.0 |
| 7 | 134459253 | 134459367 | 114 | 22 | 2.70E-05 | 25 | 27 | 1.9 |
| 7 | 139523659 | 139523753 | 94 | 36 | 1.70E-05 | 22 | 25 | 2.4 |
| 7 | 141073224 | 141073552 | 328 | 93 | 9.40E-13 | 16 | 17 | 1.2 |
| 7 | 141073553 | 141073975 | 422 | 65 | 6.50E-11 | 13 | 14 | 0.8 |
| 7 | 141073979 | 141074185 | 206 | 50 | 8.00E-08 | 18 | 20 | 1.5 |
| 7 | 141074263 | 141074706 | 443 | 87 | 2.60E-24 | 24 | 26 | 2.1 |
| 7 | 142797062 | 142797321 | 259 | 60 | 1.20E-13 | 10 | 12 | 1.5 |
| 7 | 149714454 | 149714678 | 224 | 44 | 0.00014 | 19 | 20 | 1.7 |
| 7 | 150047372 | 150047484 | 112 | 30 | 5.50E-05 | 13 | 15 | 2.1 |
| 7 | 150220231 | 150220400 | 169 | 19 | 0.001 | 23 | 26 | 2.2 |
| 7 | 150800257 | 150800616 | 359 | 92 | 2.00E-05 | 14 | 16 | 1.7 |
| 7 | 150974691 | 150974990 | 299 | 78 | 3.50E-06 | 15 | 17 | 1.3 |
| 7 | 151018834 | 151018930 | 96 | 36 | 0.0023 | 17 | 19 | 1.8 |
| 7 | 151051196 | 151051306 | 110 | 34 | 0.00013 | 19 | 21 | 2.3 |
| 7 | 151632016 | 151632296 | 280 | 81 | 0.0047 | 14 | 15 | 0.7 |
| 7 | 151632371 | 151632744 | 373 | 85 | 2.30E-12 | 22 | 22 | 0.6 |
| 7 | 157009645 | 157010029 | 384 | 113 | 7.10E-07 | 16 | 17 | 1.5 |
| 7 | 157010029 | 157010424 | 395 | 138 | 1.40E-07 | 15 | 16 | 1.0 |
| 7 | 157690510 | 157690650 | 140 | 45 | 2.20E-06 | 14 | 16 | 1.4 |
| 7 | 157690677 | 157691063 | 386 | 130 | 4.20E-39 | 22 | 23 | 1.3 |
| 8 | 1973676 | 1974211 | 535 | 103 | 0.012 | 10 | 10 | 0.4 |
| 8 | 2001417 | 2001552 | 135 | 49 | 1.90E-07 | 21 | 21 | 0.5 |
| 8 | 2001552 | 2001925 | 373 | 126 | 1.30E-19 | 17 | 19 | 1.8 |
| 8 | 7680076 | 7680210 | 134 | 30 | 0.00071 | 16 | 18 | 2.0 |
| 8 | 9906500 | 9906601 | 101 | 26 | 0.00041 | 15 | 17 | 1.5 |
| 8 | 9906613 | 9906696 | 83 | 30 | 2.70E-12 | 24 | 25 | 1.5 |
| 8 | 10729938 | 10730112 | 174 | 40 | 0.0003 | 15 | 16 | 1.2 |
| 8 | 11347544 | 11347698 | 154 | 48 | 8.00E-06 | 20 | 21 | 1.6 |
| 8 | 11347719 | 11347938 | 219 | 58 | 8.70E-06 | 12 | 13 | 1.3 |
| 8 | 21788997 | 21789245 | 248 | 66 | 8.20E-10 | 13 | 14 | 1.3 |
| 8 | 21789838 | 21789970 | 132 | 28 | 0.0035 | 14 | 17 | 2.2 |
| 8 | 22130421 | 22130815 | 394 | 113 | 0.0005 | 13 | 15 | 2.1 |
| 8 | 22551559 | 22551826 | 267 | 73 | 7.20E-09 | 16 | 17 | 0.8 |
| 8 | 22551826 | 22551995 | 169 | 51 | 8.00E-05 | 23 | 24 | 1.0 |
| 8 | 31032866 | 31032976 | 110 | 42 | 2.60E-06 | 19 | 20 | 1.2 |
| 8 | 32548273 | 32548561 | 288 | 82 | 5.80E-13 | 15 | 18 | 2.4 |
| 8 | 38465841 | 38466734 | 893 | 104 | 1.90E-09 | 18 | 19 | 0.7 |
| 8 | 38757252 | 38757713 | 461 | 85 | 2.40E-05 | 19 | 21 | 2.1 |
| 8 | 52565048 | 52565162 | 114 | 30 | 0.017 | 12 | 14 | 1.8 |
| 8 | 53880111 | 53880377 | 266 | 47 | 2.80E-06 | 21 | 24 | 2.4 |
| 8 | 53880745 | 53880963 | 218 | 48 | 0.0023 | 11 | 12 | 1.0 |
| 8 | 53880980 | 53881376 | 396 | 102 | 0.00022 | 6 | 8 | 2.0 |
| 8 | 57994529 | 57994781 | 252 | 73 | 0.028 | 13 | 14 | 1.0 |
| 8 | 59117796 | 59118336 | 540 | 93 | 6.40E-22 | 26 | 28 | 2.4 |
| 8 | 59119242 | 59119385 | 143 | 33 | 0.014 | 13 | 14 | 0.5 |
| 8 | 64580444 | 64580797 | 353 | 89 | 1.90E-08 | 16 | 17 | 1.2 |
| 8 | 64580797 | 64581001 | 204 | 51 | 0.00085 | 12 | 13 | 1.1 |
| 8 | 64798160 | 64798874 | 714 | 125 | 3.70E-12 | 14 | 17 | 2.2 |
| 8 | 71843730 | 71844022 | 292 | 60 | 2.30E-27 | 17 | 19 | 2.2 |
| 8 | 74984472 | 74984609 | 137 | 41 | 5.20E-06 | 21 | 23 | 1.8 |
| 8 | 79611985 | 79612168 | 183 | 33 | 3.40E-11 | 19 | 21 | 1.4 |
| 8 | 81280427 | 81280836 | 409 | 86 | 4.40E-08 | 14 | 16 | 1.1 |
| 8 | 84183177 | 84183278 | 101 | 21 | 0.033 | 11 | 13 | 1.5 |
| 8 | 90984976 | 90985137 | 161 | 27 | 7.90E-15 | 22 | 23 | 1.9 |
| 8 | 92102829 | 92103084 | 255 | 84 | 5.80E-63 | 31 | 34 | 2.3 |
| 8 | 94642312 | 94642593 | 281 | 36 | 0.00037 | 21 | 23 | 2.3 |
| 8 | 96157535 | 96157723 | 188 | 34 | 0.00068 | 11 | 14 | 2.3 |
| 8 | 96494911 | 96495555 | 644 | 110 | 7.30E-15 | 15 | 16 | 0.8 |
| 8 | 98426870 | 98427279 | 409 | 139 | 8.70E-35 | 22 | 24 | 1.8 |
| 8 | 103499990 | 103500080 | 90 | 28 | 7.00E-07 | 18 | 19 | 1.4 |
| 8 | 103500333 | 103500421 | 88 | 23 | 0.016 | 16 | 16 | 0.6 |
| 8 | 108083071 | 108083235 | 164 | 33 | 4.60E-05 | 20 | 22 | 2.3 |
| 8 | 109974255 | 109974357 | 102 | 16 | 0.0028 | 14 | 16 | 1.9 |
| 8 | 123160904 | 123161130 | 226 | 40 | 1.50E-14 | 18 | 19 | 1.2 |
| 8 | 132480339 | 132480459 | 120 | 38 | 3.90E-05 | 17 | 18 | 0.9 |
| 8 | 139703925 | 139704026 | 101 | 44 | 0.00022 | 17 | 19 | 1.6 |
| 8 | 142451837 | 142452102 | 265 | 73 | 0.0025 | 13 | 14 | 1.5 |
| 8 | 142510397 | 142510597 | 200 | 50 | 1.80E-12 | 21 | 24 | 2.2 |
| 8 | 143771850 | 143771972 | 122 | 38 | 0.0033 | 21 | 23 | 1.8 |
| 8 | 143814793 | 143816225 | 1432 | 334 | 1.20E-06 | 10 | 11 | 0.5 |
| 9 | 113739 | 113916 | 177 | 67 | 5.90E-39 | 26 | 28 | 2.1 |
| 9 | 113917 | 114628 | 711 | 150 | 2.50E-49 | 21 | 23 | 2.3 |
| 9 | 504290 | 504415 | 125 | 39 | 0.0041 | 21 | 22 | 0.8 |
| 9 | 504487 | 504923 | 436 | 144 | 1.90E-13 | 16 | 17 | 0.8 |
| 9 | 505385 | 505662 | 277 | 54 | 0.044 | 14 | 16 | 2.3 |
| 9 | 17579168 | 17579461 | 293 | 79 | 0.02 | 13 | 15 | 1.9 |
| 9 | 20620450 | 20620550 | 100 | 38 | 2.70E-06 | 25 | 26 | 1.1 |
| 9 | 20620553 | 20620951 | 398 | 62 | 0.011 | 18 | 19 | 1.0 |
| 9 | 21974774 | 21975199 | 425 | 61 | 6.90E-07 | 10 | 11 | 1.0 |
| 9 | 29212199 | 29212295 | 96 | 36 | 3.00E-06 | 14 | 15 | 1.6 |
| 9 | 34589040 | 34589128 | 88 | 30 | 9.20E-05 | 22 | 24 | 2.0 |
| 9 | 35675632 | 35675796 | 164 | 24 | 4.90E-08 | 19 | 20 | 1.5 |
| 9 | 35675796 | 35675914 | 118 | 24 | 1.80E-15 | 30 | 33 | 2.2 |
| 9 | 37034613 | 37034699 | 86 | 16 | 1.30E-06 | 26 | 28 | 1.6 |
| 9 | 38067271 | 38067858 | 587 | 74 | 4.20E-05 | 21 | 22 | 1.2 |
| 9 | 38621332 | 38622467 | 1135 | 176 | 1.60E-14 | 18 | 20 | 2.2 |
| 9 | 42129324 | 42129476 | 152 | 45 | 8.40E-06 | 10 | 12 | 1.3 |
| 9 | 74497813 | 74498300 | 487 | 69 | 0.013 | 10 | 11 | 1.0 |
| 9 | 86946014 | 86946247 | 233 | 52 | 1.50E-07 | 11 | 12 | 1.6 |
| 9 | 88991564 | 88991809 | 245 | 66 | 2.80E-10 | 14 | 15 | 0.7 |
| 9 | 94639132 | 94639937 | 805 | 141 | 1.70E-10 | 19 | 20 | 1.4 |
| 9 | 96021025 | 96021890 | 865 | 192 | 3.60E-06 | 11 | 12 | 0.7 |
| 9 | 97221614 | 97221902 | 288 | 66 | 2.10E-11 | 16 | 17 | 1.2 |
| 9 | 98708625 | 98708804 | 179 | 61 | 2.70E-06 | 17 | 19 | 1.5 |
| 9 | 98708899 | 98709150 | 251 | 84 | 1.70E-23 | 19 | 20 | 1.0 |
| 9 | 98709250 | 98709396 | 146 | 39 | 0.0003 | 17 | 18 | 1.4 |
| 9 | 98807653 | 98807820 | 167 | 54 | 0.00093 | 18 | 20 | 1.2 |
| 9 | 100472942 | 100473124 | 182 | 50 | 3.00E-07 | 17 | 19 | 1.7 |
| 9 | 100473175 | 100473319 | 144 | 50 | 3.30E-06 | 20 | 21 | 1.9 |
| 9 | 107465806 | 107466111 | 305 | 53 | 1.70E-07 | 19 | 21 | 2.2 |
| 9 | 114154059 | 114154246 | 187 | 64 | 0.00014 | 17 | 19 | 1.4 |
| 9 | 121299849 | 121300161 | 312 | 66 | 0.0022 | 16 | 17 | 0.5 |
| 9 | 126614101 | 126614315 | 214 | 70 | 8.60E-06 | 17 | 18 | 1.3 |
| 9 | 129620435 | 129620675 | 240 | 52 | 0.00024 | 8 | 9 | 1.1 |
| 9 | 130938803 | 130939047 | 244 | 56 | 0.0032 | 9 | 9 | 0.6 |
| 9 | 131276788 | 131276906 | 118 | 47 | 3.00E-05 | 15 | 16 | 1.5 |
| 9 | 131282773 | 131282898 | 125 | 32 | 0.0043 | 19 | 21 | 2.4 |
| 9 | 131546331 | 131546434 | 103 | 27 | 0.0053 | 18 | 20 | 2.0 |
| 9 | 132161407 | 132161514 | 107 | 24 | 0.013 | 19 | 21 | 2.4 |
| 9 | 132410381 | 132410579 | 198 | 54 | 2.20E-06 | 16 | 19 | 2.3 |
| 9 | 133428620 | 133428817 | 197 | 58 | 1.00E-14 | 22 | 23 | 0.9 |
| 9 | 134163224 | 134164057 | 833 | 158 | 0.00014 | 13 | 15 | 2.4 |
| 9 | 134642139 | 134642227 | 88 | 35 | 6.60E-11 | 22 | 24 | 2.2 |
| 9 | 136193583 | 136193734 | 151 | 52 | 1.00E-11 | 17 | 18 | 1.5 |
| 9 | 137406863 | 137407459 | 596 | 115 | 9.10E-08 | 20 | 21 | 1.1 |
| 10 | 3067001 | 3067337 | 336 | 60 | 0.029 | 16 | 18 | 1.9 |
| 10 | 3069026 | 3069372 | 346 | 69 | 0.019 | 16 | 19 | 2.3 |
| 10 | 6200613 | 6201200 | 587 | 77 | 5.90E-07 | 19 | 20 | 0.6 |
| 10 | 8054479 | 8054610 | 131 | 17 | 0.022 | 23 | 24 | 1.3 |
| 10 | 11017968 | 11018319 | 351 | 124 | 2.90E-22 | 16 | 17 | 0.6 |
| 10 | 12348663 | 12349020 | 357 | 65 | 0.026 | 14 | 15 | 1.5 |
| 10 | 13891521 | 13891734 | 213 | 44 | 1.10E-18 | 22 | 25 | 2.4 |
| 10 | 13891786 | 13891884 | 98 | 36 | 0.00092 | 16 | 16 | 0.4 |
| 10 | 13891884 | 13892001 | 117 | 47 | 3.60E-24 | 28 | 30 | 2.0 |
| 10 | 16520711 | 16521318 | 607 | 130 | 1.90E-59 | 27 | 29 | 2.1 |
| 10 | 17228350 | 17228524 | 174 | 30 | 0.00092 | 25 | 26 | 1.5 |
| 10 | 17228724 | 17228902 | 178 | 42 | 9.50E-10 | 17 | 18 | 1.0 |
| 10 | 18140277 | 18140621 | 344 | 102 | 8.30E-14 | 11 | 12 | 1.6 |
| 10 | 18140713 | 18140916 | 203 | 49 | 0.00068 | 15 | 16 | 1.4 |
| 10 | 21173971 | 21174233 | 262 | 83 | 4.60E-05 | 14 | 15 | 0.8 |
| 10 | 22252618 | 22252770 | 152 | 31 | 2.50E-09 | 22 | 22 | 0.6 |
| 10 | 22252786 | 22252937 | 151 | 42 | 7.20E-23 | 32 | 34 | 1.2 |
| 10 | 23694566 | 23694650 | 84 | 26 | 0.0001 | 19 | 20 | 1.0 |
| 10 | 24952359 | 24952631 | 272 | 52 | 0.00063 | 18 | 19 | 1.2 |
| 10 | 27744440 | 27744878 | 438 | 107 | 0.00071 | 13 | 16 | 2.3 |
| 10 | 27745913 | 27745996 | 83 | 29 | 0.00011 | 23 | 25 | 1.8 |
| 10 | 27998826 | 27999091 | 265 | 80 | 1.80E-08 | 15 | 16 | 1.4 |
| 10 | 30784913 | 30785291 | 378 | 83 | 0.0053 | 13 | 14 | 0.7 |
| 10 | 31134012 | 31134211 | 199 | 30 | 0.0049 | 29 | 31 | 2.0 |
| 10 | 31319826 | 31320038 | 212 | 94 | 6.10E-16 | 11 | 11 | 0.4 |
| 10 | 35640562 | 35641152 | 590 | 177 | 4.40E-18 | 15 | 15 | 0.5 |
| 10 | 42753798 | 42754207 | 409 | 76 | 0.00022 | 14 | 16 | 1.7 |
| 10 | 42754425 | 42754616 | 191 | 30 | 1.10E-05 | 21 | 23 | 2.1 |
| 10 | 43077389 | 43077512 | 123 | 34 | 0.032 | 16 | 16 | 0.7 |
| 10 | 43266828 | 43266923 | 95 | 44 | 0.015 | 19 | 21 | 2.2 |
| 10 | 43267012 | 43267267 | 255 | 74 | 4.60E-07 | 16 | 18 | 2.3 |
| 10 | 45419014 | 45419292 | 278 | 54 | 0.0019 | 13 | 14 | 0.8 |
| 10 | 46578641 | 46578925 | 284 | 44 | 0.022 | 12 | 13 | 1.2 |
| 10 | 48523366 | 48523743 | 377 | 77 | 1.40E-10 | 14 | 16 | 1.8 |
| 10 | 48523919 | 48524284 | 365 | 95 | 1.10E-10 | 15 | 16 | 1.0 |
| 10 | 51074741 | 51074922 | 181 | 34 | 0.00035 | 17 | 19 | 2.2 |
| 10 | 59176592 | 59176972 | 380 | 59 | 0.00067 | 13 | 15 | 2.2 |
| 10 | 62814953 | 62815263 | 310 | 66 | 3.00E-08 | 17 | 19 | 2.0 |
| 10 | 77637918 | 77638137 | 219 | 88 | 7.80E-05 | 8 | 9 | 1.0 |
| 10 | 77638414 | 77638737 | 323 | 72 | 3.70E-06 | 15 | 17 | 2.4 |
| 10 | 81874064 | 81874186 | 122 | 22 | 9.30E-05 | 25 | 27 | 1.7 |
| 10 | 81874417 | 81874567 | 150 | 40 | 9.10E-10 | 21 | 23 | 1.9 |
| 10 | 81875193 | 81875395 | 202 | 69 | 1.50E-17 | 24 | 26 | 1.6 |
| 10 | 86366012 | 86366791 | 779 | 229 | 7.50E-17 | 13 | 14 | 0.8 |
| 10 | 86366914 | 86367276 | 362 | 93 | 1.10E-09 | 13 | 14 | 1.1 |
| 10 | 89535366 | 89535590 | 224 | 43 | 0.00096 | 22 | 25 | 2.1 |
| 10 | 89535686 | 89535834 | 148 | 44 | 7.30E-10 | 24 | 26 | 1.2 |
| 10 | 91887392 | 91888028 | 636 | 90 | 2.00E-05 | 18 | 20 | 2.3 |
| 10 | 96043287 | 96043678 | 391 | 111 | 4.50E-06 | 10 | 11 | 0.5 |
| 10 | 97185791 | 97186019 | 228 | 44 | 0.015 | 16 | 18 | 2.4 |
| 10 | 97320104 | 97320837 | 733 | 93 | 2.10E-05 | 18 | 20 | 1.7 |
| 10 | 98030915 | 98031119 | 204 | 36 | 1.80E-23 | 20 | 22 | 1.5 |
| 10 | 99329374 | 99329583 | 209 | 48 | 4.70E-13 | 19 | 21 | 1.5 |
| 10 | 99329635 | 99329739 | 104 | 30 | 0.013 | 16 | 18 | 1.9 |
| 10 | 99329780 | 99330180 | 400 | 102 | 1.30E-09 | 12 | 14 | 1.9 |
| 10 | 99535288 | 99535513 | 225 | 74 | 0.001 | 17 | 19 | 1.4 |
| 10 | 101775510 | 101776101 | 591 | 155 | 4.40E-26 | 17 | 19 | 2.2 |
| 10 | 102408853 | 102409059 | 206 | 58 | 0.00044 | 13 | 14 | 0.6 |
| 10 | 103277088 | 103277189 | 101 | 29 | 1.10E-14 | 31 | 32 | 1.3 |
| 10 | 111077729 | 111078112 | 383 | 91 | 4.90E-07 | 15 | 17 | 2.2 |
| 10 | 114042970 | 114043480 | 510 | 61 | 0.0028 | 13 | 13 | 0.8 |
| 10 | 114404504 | 114404786 | 282 | 82 | 0.023 | 11 | 13 | 2.3 |
| 10 | 116272112 | 116272623 | 511 | 80 | 3.50E-07 | 14 | 16 | 2.0 |
| 10 | 116273246 | 116273579 | 333 | 58 | 0.00044 | 12 | 14 | 1.8 |
| 10 | 129963667 | 129964117 | 450 | 128 | 9.10E-06 | 13 | 14 | 1.6 |
| 10 | 129964223 | 129964499 | 276 | 94 | 0.023 | 12 | 13 | 1.9 |
| 10 | 129971592 | 129971795 | 203 | 53 | 0.004 | 16 | 18 | 1.8 |
| 10 | 129971795 | 129972125 | 330 | 59 | 0.019 | 11 | 12 | 1.5 |
| 10 | 132036632 | 132036738 | 106 | 39 | 2.50E-05 | 17 | 18 | 1.0 |
| 10 | 132307334 | 132307539 | 205 | 60 | 0.0014 | 16 | 18 | 1.4 |
| 10 | 132942528 | 132942716 | 188 | 51 | 1.10E-05 | 15 | 17 | 2.0 |
| 11 | 2884167 | 2884292 | 125 | 47 | 0.0045 | 17 | 19 | 1.6 |
| 11 | 2884913 | 2885517 | 604 | 166 | 3.70E-18 | 16 | 18 | 1.3 |
| 11 | 7251923 | 7252159 | 236 | 49 | 2.00E-10 | 16 | 18 | 1.6 |
| 11 | 7252220 | 7252544 | 324 | 54 | 2.40E-16 | 18 | 20 | 2.0 |
| 11 | 8019167 | 8019266 | 99 | 26 | 0.017 | 20 | 23 | 2.2 |
| 11 | 8019286 | 8019432 | 146 | 27 | 0.024 | 22 | 24 | 1.6 |
| 11 | 8019748 | 8019983 | 235 | 71 | 0.0046 | 12 | 13 | 1.3 |
| 11 | 8080962 | 8081119 | 157 | 39 | 0.0057 | 16 | 17 | 1.7 |
| 11 | 8081204 | 8081420 | 216 | 74 | 2.30E-14 | 15 | 16 | 1.8 |
| 11 | 8262862 | 8263080 | 218 | 56 | 9.10E-08 | 16 | 17 | 1.0 |
| 11 | 8594107 | 8594234 | 127 | 31 | 4.60E-05 | 17 | 19 | 1.4 |
| 11 | 13963110 | 13963461 | 351 | 96 | 5.40E-88 | 29 | 31 | 1.8 |
| 11 | 14973792 | 14973873 | 81 | 17 | 0.0012 | 23 | 25 | 2.1 |
| 11 | 15114726 | 15114848 | 122 | 25 | 5.20E-07 | 31 | 33 | 2.4 |
| 11 | 16606632 | 16607339 | 707 | 115 | 0.00029 | 11 | 14 | 2.4 |
| 11 | 17351380 | 17352250 | 870 | 145 | 1.80E-14 | 15 | 18 | 2.3 |
| 11 | 18721114 | 18721698 | 584 | 90 | 4.30E-06 | 13 | 15 | 1.9 |
| 11 | 18791679 | 18791768 | 89 | 35 | 0.0018 | 17 | 19 | 2.1 |
| 11 | 32435018 | 32435100 | 82 | 22 | 4.80E-08 | 20 | 22 | 2.4 |
| 11 | 32893709 | 32893968 | 259 | 51 | 0.0038 | 10 | 10 | 0.1 |
| 11 | 35619292 | 35619738 | 446 | 85 | 4.20E-10 | 15 | 16 | 1.7 |
| 11 | 44727129 | 44727221 | 92 | 39 | 0.026 | 18 | 20 | 1.7 |
| 11 | 45665057 | 45665307 | 250 | 77 | 0.00079 | 11 | 12 | 1.0 |
| 11 | 46237800 | 46238011 | 211 | 44 | 0.0026 | 18 | 21 | 2.2 |
| 11 | 46295374 | 46295588 | 214 | 43 | 8.50E-07 | 14 | 16 | 2.1 |
| 11 | 47187051 | 47187760 | 709 | 95 | 1.60E-07 | 21 | 23 | 2.2 |
| 11 | 57476287 | 57476838 | 551 | 148 | 0.0099 | 10 | 11 | 1.0 |
| 11 | 57638297 | 57638505 | 208 | 17 | 0.0016 | 18 | 19 | 1.9 |
| 11 | 63491044 | 63491246 | 202 | 59 | 5.20E-23 | 27 | 28 | 1.1 |
| 11 | 64000527 | 64000627 | 100 | 26 | 0.00033 | 25 | 27 | 1.7 |
| 11 | 64035885 | 64036481 | 596 | 146 | 5.40E-06 | 12 | 13 | 1.3 |
| 11 | 65786552 | 65786714 | 162 | 49 | 2.60E-10 | 17 | 19 | 2.0 |
| 11 | 65787048 | 65787260 | 212 | 68 | 8.60E-05 | 16 | 18 | 1.6 |
| 11 | 65833665 | 65833921 | 256 | 49 | 3.80E-07 | 12 | 12 | 0.7 |
| 11 | 66856962 | 66857267 | 305 | 112 | 0.0016 | 12 | 14 | 1.9 |
| 11 | 67118134 | 67118559 | 425 | 111 | 9.00E-07 | 18 | 20 | 2.1 |
| 11 | 75241565 | 75241707 | 142 | 42 | 0.0042 | 11 | 11 | 0.9 |
| 11 | 75242002 | 75242204 | 202 | 64 | 0.00042 | 14 | 17 | 2.3 |
| 11 | 75668507 | 75668635 | 128 | 26 | 0.0013 | 21 | 23 | 1.9 |
| 11 | 82733257 | 82733422 | 165 | 57 | 0.0004 | 15 | 17 | 1.8 |
| 11 | 86671371 | 86672247 | 876 | 114 | 2.90E-05 | 14 | 16 | 1.9 |
| 11 | 86672247 | 86672513 | 266 | 50 | 4.90E-05 | 17 | 18 | 1.2 |
| 11 | 88174760 | 88175207 | 447 | 54 | 2.90E-14 | 20 | 21 | 0.9 |
| 11 | 88175219 | 88175303 | 84 | 18 | 0.00068 | 24 | 27 | 2.3 |
| 11 | 93197237 | 93198146 | 909 | 159 | 1.10E-05 | 12 | 12 | 0.4 |
| 11 | 94740324 | 94740654 | 330 | 98 | 5.50E-15 | 14 | 16 | 2.1 |
| 11 | 94740661 | 94740805 | 144 | 38 | 7.80E-07 | 17 | 18 | 1.2 |
| 11 | 104163821 | 104164033 | 212 | 45 | 9.30E-30 | 31 | 31 | 0.8 |
| 11 | 104164033 | 104164477 | 444 | 73 | 1.50E-29 | 24 | 26 | 1.3 |
| 11 | 106021503 | 106022099 | 596 | 56 | 0.025 | 11 | 13 | 1.6 |
| 11 | 107018174 | 107018439 | 265 | 93 | 0.0051 | 13 | 14 | 1.4 |
| 11 | 110711794 | 110712119 | 325 | 91 | 1.30E-08 | 14 | 14 | 0.6 |
| 11 | 110712274 | 110712468 | 194 | 68 | 0.0038 | 13 | 13 | 0.2 |
| 11 | 110712970 | 110713438 | 468 | 45 | 9.90E-06 | 19 | 20 | 0.8 |
| 11 | 111298997 | 111299141 | 144 | 39 | 6.10E-12 | 27 | 29 | 2.2 |
| 11 | 111299179 | 111299853 | 674 | 120 | 2.00E-06 | 16 | 17 | 1.0 |
| 11 | 114058891 | 114059150 | 259 | 39 | 0.0003 | 17 | 19 | 2.2 |
| 11 | 123358155 | 123358570 | 415 | 51 | 0.0092 | 8 | 9 | 1.2 |
| 11 | 123430574 | 123430680 | 106 | 37 | 2.00E-07 | 18 | 19 | 0.9 |
| 11 | 123430737 | 123430943 | 206 | 54 | 7.40E-26 | 18 | 19 | 1.2 |
| 11 | 128686970 | 128687074 | 104 | 29 | 0.026 | 14 | 16 | 1.5 |
| 11 | 128692988 | 128693150 | 162 | 24 | 0.006 | 13 | 15 | 2.2 |
| 11 | 128693174 | 128693294 | 120 | 31 | 7.70E-14 | 18 | 19 | 1.5 |
| 11 | 128693294 | 128693475 | 181 | 33 | 2.20E-05 | 13 | 15 | 1.9 |
| 11 | 128693818 | 128694170 | 352 | 29 | 5.20E-06 | 16 | 18 | 1.7 |
| 11 | 128694193 | 128694642 | 449 | 80 | 3.20E-20 | 19 | 20 | 1.4 |
| 11 | 128694805 | 128694893 | 88 | 12 | 1.20E-07 | 32 | 32 | 0.8 |
| 11 | 128694901 | 128695018 | 117 | 31 | 8.10E-11 | 22 | 22 | 0.9 |
| 11 | 130427605 | 130427696 | 91 | 21 | 0.015 | 21 | 24 | 2.4 |
| 11 | 134069058 | 134069177 | 119 | 23 | 1.70E-16 | 16 | 16 | 0.9 |
| 11 | 134276299 | 134276401 | 102 | 33 | 9.90E-08 | 22 | 24 | 1.8 |
| 12 | 460640 | 460840 | 200 | 54 | 1.30E-05 | 18 | 20 | 1.4 |
| 12 | 2052400 | 2052644 | 244 | 74 | 1.80E-05 | 15 | 16 | 1.3 |
| 12 | 2053435 | 2053754 | 319 | 64 | 3.90E-05 | 14 | 16 | 1.5 |
| 12 | 2053964 | 2054125 | 161 | 59 | 3.20E-11 | 18 | 19 | 1.2 |
| 12 | 4164733 | 4164815 | 82 | 21 | 0.01 | 21 | 23 | 1.6 |
| 12 | 4269236 | 4269349 | 113 | 40 | 0.0013 | 18 | 20 | 1.9 |
| 12 | 4910555 | 4910840 | 285 | 33 | 7.70E-06 | 16 | 18 | 1.8 |
| 12 | 5432103 | 5432318 | 215 | 38 | 9.20E-09 | 18 | 20 | 2.3 |
| 12 | 5433044 | 5433216 | 172 | 35 | 3.80E-14 | 22 | 24 | 2.2 |
| 12 | 5433222 | 5433357 | 135 | 26 | 0.01 | 15 | 17 | 1.7 |
| 12 | 26195640 | 26196113 | 473 | 71 | 0.043 | 11 | 12 | 0.7 |
| 12 | 27969954 | 27970359 | 405 | 79 | 1.20E-05 | 10 | 11 | 1.0 |
| 12 | 27970517 | 27970638 | 121 | 37 | 0.013 | 17 | 18 | 0.9 |
| 12 | 42483219 | 42483564 | 345 | 81 | 0.034 | 12 | 13 | 0.7 |
| 12 | 48004123 | 48004408 | 285 | 63 | 0.0045 | 17 | 18 | 1.7 |
| 12 | 48004445 | 48004805 | 360 | 73 | 9.90E-12 | 20 | 22 | 2.0 |
| 12 | 48183702 | 48183938 | 236 | 67 | 4.90E-09 | 16 | 18 | 1.2 |
| 12 | 48904034 | 48904365 | 331 | 35 | 2.90E-08 | 18 | 20 | 2.4 |
| 12 | 52006817 | 52006907 | 90 | 26 | 0.00033 | 17 | 17 | 0.4 |
| 12 | 52006941 | 52007189 | 248 | 66 | 2.40E-41 | 29 | 30 | 1.3 |
| 12 | 59596452 | 59597341 | 889 | 104 | 0.00014 | 10 | 10 | 0.8 |
| 12 | 63667846 | 63668605 | 759 | 86 | 2.50E-09 | 12 | 14 | 1.7 |
| 12 | 63668615 | 63668726 | 111 | 21 | 0.011 | 19 | 19 | 0.3 |
| 12 | 64824238 | 64824943 | 705 | 138 | 2.30E-79 | 25 | 27 | 2.1 |
| 12 | 65741882 | 65742515 | 633 | 125 | 2.60E-36 | 19 | 21 | 2.2 |
| 12 | 66189062 | 66189295 | 233 | 54 | 3.10E-11 | 17 | 19 | 2.1 |
| 12 | 66235225 | 66235363 | 138 | 42 | 0.0096 | 22 | 24 | 1.8 |
| 12 | 98457037 | 98457291 | 254 | 55 | 1.20E-06 | 22 | 23 | 0.8 |
| 12 | 98894954 | 98895048 | 94 | 36 | 1.60E-05 | 27 | 29 | 2.1 |
| 12 | 102958180 | 102958685 | 505 | 127 | 1.20E-16 | 16 | 18 | 1.9 |
| 12 | 104215573 | 104216143 | 570 | 114 | 5.30E-14 | 21 | 23 | 1.4 |
| 12 | 104216147 | 104216257 | 110 | 28 | 7.90E-16 | 36 | 38 | 2.0 |
| 12 | 104458268 | 104458548 | 280 | 44 | 3.30E-10 | 20 | 22 | 1.7 |
| 12 | 106583538 | 106583619 | 81 | 22 | 2.00E-07 | 24 | 27 | 2.4 |
| 12 | 106585947 | 106586282 | 335 | 82 | 9.30E-28 | 18 | 20 | 2.0 |
| 12 | 109833376 | 109833550 | 174 | 57 | 1.70E-05 | 16 | 17 | 1.0 |
| 12 | 111034815 | 111034912 | 97 | 42 | 2.00E-06 | 17 | 18 | 1.2 |
| 12 | 113056773 | 113057013 | 240 | 72 | 0.021 | 12 | 12 | 0.5 |
| 12 | 113154553 | 113154640 | 87 | 17 | 0.0017 | 17 | 19 | 2.0 |
| 12 | 121536806 | 121537436 | 630 | 81 | 0.00021 | 16 | 17 | 0.6 |
| 12 | 127166252 | 127166390 | 138 | 43 | 9.50E-05 | 10 | 12 | 1.8 |
| 12 | 128854060 | 128854247 | 187 | 24 | 0.0047 | 15 | 16 | 1.1 |
| 12 | 130162062 | 130162196 | 134 | 29 | 2.10E-06 | 17 | 19 | 2.4 |
| 12 | 132887725 | 132888332 | 607 | 102 | 8.30E-19 | 13 | 14 | 1.0 |
| 12 | 132908345 | 132908461 | 116 | 38 | 2.60E-23 | 35 | 37 | 2.0 |
| 12 | 132908545 | 132908635 | 90 | 15 | 4.10E-08 | 29 | 31 | 2.1 |
| 12 | 132908635 | 132908844 | 209 | 27 | 3.60E-15 | 22 | 25 | 2.3 |
| 12 | 132909104 | 132909208 | 104 | 20 | 5.60E-05 | 22 | 24 | 2.4 |
| 13 | 21075676 | 21075914 | 238 | 27 | 0.00029 | 25 | 26 | 1.0 |
| 13 | 25371997 | 25372105 | 108 | 40 | 0.0003 | 19 | 21 | 1.9 |
| 13 | 25372160 | 25372309 | 149 | 41 | 8.50E-08 | 20 | 22 | 1.5 |
| 13 | 25372341 | 25372499 | 158 | 41 | 2.20E-07 | 21 | 23 | 2.1 |
| 13 | 26557692 | 26557887 | 195 | 70 | 0.0046 | 13 | 15 | 1.1 |
| 13 | 28100382 | 28100617 | 235 | 66 | 4.40E-17 | 25 | 26 | 1.3 |
| 13 | 28100755 | 28101019 | 264 | 56 | 2.30E-11 | 13 | 15 | 1.7 |
| 13 | 28494184 | 28495140 | 956 | 203 | 7.40E-09 | 12 | 14 | 1.7 |
| 13 | 28532192 | 28532505 | 313 | 70 | 8.40E-14 | 19 | 21 | 2.4 |
| 13 | 32426704 | 32427687 | 983 | 151 | 0.00045 | 13 | 14 | 0.7 |
| 13 | 33016291 | 33016557 | 266 | 86 | 3.40E-07 | 18 | 20 | 2.4 |
| 13 | 36345930 | 36346263 | 333 | 54 | 2.60E-08 | 11 | 13 | 1.9 |
| 13 | 36346348 | 36346494 | 146 | 61 | 0.00032 | 9 | 10 | 1.1 |
| 13 | 36920065 | 36920471 | 406 | 135 | 2.20E-05 | 13 | 14 | 0.7 |
| 13 | 38687162 | 38687527 | 365 | 71 | 1.80E-05 | 14 | 16 | 2.3 |
| 13 | 41457099 | 41457441 | 342 | 81 | 2.40E-05 | 15 | 16 | 1.2 |
| 13 | 41457868 | 41458784 | 916 | 154 | 0.044 | 11 | 12 | 1.4 |
| 13 | 42574340 | 42574634 | 294 | 49 | 9.70E-06 | 18 | 20 | 2.2 |
| 13 | 42575058 | 42575145 | 87 | 19 | 7.40E-05 | 20 | 22 | 2.0 |
| 13 | 43879841 | 43880031 | 190 | 47 | 0.003 | 9 | 9 | 0.5 |
| 13 | 44373923 | 44374238 | 315 | 58 | 3.30E-18 | 21 | 23 | 1.8 |
| 13 | 49220134 | 49220315 | 181 | 54 | 7.70E-06 | 16 | 18 | 2.0 |
| 13 | 52850807 | 52851072 | 265 | 52 | 2.40E-07 | 18 | 20 | 1.8 |
| 13 | 76885229 | 76885770 | 541 | 138 | 8.60E-07 | 14 | 16 | 1.2 |
| 13 | 77697816 | 77698076 | 260 | 68 | 0.0023 | 15 | 15 | 0.4 |
| 13 | 77698117 | 77698405 | 288 | 70 | 0.0018 | 12 | 12 | 0.2 |
| 13 | 78608987 | 78609310 | 323 | 37 | 9.50E-05 | 15 | 17 | 2.0 |
| 13 | 87671607 | 87671938 | 331 | 65 | 8.90E-12 | 14 | 16 | 1.4 |
| 13 | 87672434 | 87672525 | 91 | 21 | 0.0046 | 19 | 20 | 1.2 |
| 13 | 87672525 | 87672985 | 460 | 46 | 7.20E-05 | 12 | 14 | 2.2 |
| 13 | 94711449 | 94711615 | 166 | 60 | 1.00E-11 | 19 | 20 | 1.4 |
| 13 | 102394580 | 102394876 | 296 | 65 | 5.50E-09 | 12 | 13 | 0.5 |
| 13 | 106534459 | 106535238 | 779 | 172 | 1.50E-29 | 21 | 23 | 2.2 |
| 13 | 106535238 | 106535477 | 239 | 82 | 7.90E-06 | 18 | 19 | 1.1 |
| 13 | 106535550 | 106535802 | 252 | 95 | 3.10E-14 | 18 | 19 | 0.6 |
| 13 | 110307262 | 110307392 | 130 | 43 | 1.00E-08 | 22 | 24 | 2.0 |
| 13 | 112689884 | 112690644 | 760 | 226 | 0.00024 | 10 | 10 | 0.3 |
| 13 | 113725093 | 113725381 | 288 | 43 | 0.0027 | 17 | 19 | 2.2 |
| 13 | 113863215 | 113863393 | 178 | 39 | 5.00E-05 | 25 | 25 | 0.6 |
| 14 | 26598230 | 26598332 | 102 | 35 | 0.031 | 11 | 13 | 2.3 |
| 14 | 35535235 | 35535531 | 296 | 52 | 3.80E-13 | 18 | 20 | 1.9 |
| 14 | 51094574 | 51094775 | 201 | 38 | 0.0017 | 10 | 12 | 2.2 |
| 14 | 52068459 | 52068810 | 351 | 46 | 3.80E-05 | 14 | 17 | 2.2 |
| 14 | 52069168 | 52069374 | 206 | 26 | 1.90E-05 | 18 | 20 | 1.9 |
| 14 | 52267701 | 52267811 | 110 | 29 | 5.60E-13 | 25 | 27 | 2.2 |
| 14 | 55128990 | 55129842 | 852 | 207 | 3.00E-30 | 20 | 22 | 1.8 |
| 14 | 56118530 | 56118895 | 365 | 104 | 0.0022 | 15 | 15 | 0.4 |
| 14 | 59464413 | 59465114 | 701 | 188 | 0.0025 | 11 | 11 | 0.5 |
| 14 | 60327711 | 60327993 | 282 | 44 | 6.70E-21 | 33 | 35 | 1.7 |
| 14 | 60721669 | 60722087 | 418 | 85 | 6.20E-06 | 22 | 24 | 1.6 |
| 14 | 61812719 | 61812830 | 111 | 40 | 7.70E-08 | 18 | 20 | 1.7 |
| 14 | 61812904 | 61813054 | 150 | 36 | 9.10E-05 | 15 | 17 | 1.3 |
| 14 | 64338787 | 64338987 | 200 | 26 | 0.035 | 11 | 11 | 0.8 |
| 14 | 70187788 | 70187896 | 108 | 33 | 4.40E-16 | 30 | 31 | 1.4 |
| 14 | 70188515 | 70188607 | 92 | 26 | 8.30E-05 | 16 | 17 | 0.9 |
| 14 | 70188846 | 70189154 | 308 | 66 | 5.80E-15 | 15 | 16 | 0.9 |
| 14 | 71932065 | 71932289 | 224 | 56 | 5.90E-08 | 14 | 15 | 1.2 |
| 14 | 71932305 | 71932875 | 570 | 160 | 4.90E-30 | 16 | 17 | 1.6 |
| 14 | 77271151 | 77271253 | 102 | 30 | 0.00056 | 19 | 21 | 2.1 |
| 14 | 77498343 | 77498790 | 447 | 115 | 0.0036 | 12 | 14 | 1.4 |
| 14 | 90060614 | 90060941 | 327 | 97 | 0.045 | 12 | 13 | 0.8 |
| 14 | 90060979 | 90061282 | 303 | 48 | 0.037 | 14 | 16 | 2.0 |
| 14 | 91418011 | 91418407 | 396 | 109 | 2.40E-11 | 18 | 20 | 1.8 |
| 14 | 92323269 | 92323411 | 142 | 46 | 0.0018 | 15 | 16 | 1.3 |
| 14 | 92323412 | 92323616 | 204 | 70 | 0.00049 | 11 | 12 | 1.4 |
| 14 | 94768688 | 94768952 | 264 | 52 | 4.00E-08 | 18 | 20 | 1.9 |
| 14 | 94769907 | 94770045 | 138 | 42 | 0.014 | 15 | 16 | 1.5 |
| 14 | 99972406 | 99972847 | 441 | 37 | 0.0056 | 19 | 21 | 2.3 |
| 14 | 100159114 | 100159291 | 177 | 54 | 2.30E-06 | 16 | 18 | 2.0 |
| 14 | 100159374 | 100159660 | 286 | 84 | 1.80E-05 | 16 | 18 | 1.2 |
| 14 | 100159751 | 100159937 | 186 | 46 | 0.00029 | 20 | 21 | 1.6 |
| 14 | 100567669 | 100567819 | 150 | 48 | 0.025 | 16 | 17 | 1.4 |
| 14 | 100568575 | 100568973 | 398 | 107 | 2.70E-06 | 13 | 15 | 2.3 |
| 14 | 101781458 | 101781974 | 516 | 103 | 1.30E-53 | 26 | 27 | 1.3 |
| 14 | 102555465 | 102555562 | 97 | 27 | 0.017 | 11 | 12 | 0.5 |
| 14 | 102928411 | 102928717 | 306 | 68 | 8.60E-06 | 15 | 16 | 1.1 |
| 14 | 103189000 | 103189109 | 109 | 32 | 2.50E-09 | 16 | 16 | 0.9 |
| 14 | 103522280 | 103522650 | 370 | 101 | 0.013 | 14 | 16 | 2.1 |
| 14 | 103522729 | 103523128 | 399 | 102 | 0.024 | 12 | 14 | 1.7 |
| 14 | 104136327 | 104136500 | 173 | 65 | 0.00012 | 14 | 16 | 1.4 |
| 14 | 104462023 | 104462103 | 80 | 23 | 2.40E-05 | 18 | 20 | 2.2 |
| 14 | 105167709 | 105168083 | 374 | 109 | 0.0083 | 13 | 14 | 1.7 |
| 15 | 25862771 | 25863201 | 430 | 126 | 4.00E-24 | 19 | 21 | 2.3 |
| 15 | 25863274 | 25863496 | 222 | 58 | 2.80E-05 | 14 | 16 | 1.8 |
| 15 | 28106686 | 28106932 | 246 | 60 | 3.90E-07 | 16 | 17 | 1.2 |
| 15 | 28106932 | 28107183 | 251 | 73 | 8.40E-18 | 20 | 21 | 1.4 |
| 15 | 29570461 | 29570734 | 273 | 86 | 0.0034 | 16 | 18 | 1.9 |
| 15 | 29571040 | 29571322 | 282 | 83 | 0.00081 | 18 | 19 | 1.4 |
| 15 | 34437153 | 34437439 | 286 | 80 | 5.80E-12 | 18 | 20 | 2.2 |
| 15 | 40283047 | 40283347 | 300 | 59 | 0.0069 | 16 | 18 | 2.0 |
| 15 | 40442112 | 40442360 | 248 | 68 | 0.008 | 18 | 19 | 0.5 |
| 15 | 45377671 | 45378549 | 878 | 159 | 1.20E-12 | 13 | 15 | 1.4 |
| 15 | 48645060 | 48645335 | 275 | 55 | 0.00085 | 14 | 15 | 1.3 |
| 15 | 48645336 | 48645614 | 278 | 64 | 0.00064 | 11 | 12 | 1.1 |
| 15 | 48645626 | 48645912 | 286 | 28 | 0.0013 | 15 | 16 | 0.7 |
| 15 | 52805050 | 52805395 | 345 | 38 | 0.015 | 15 | 17 | 2.1 |
| 15 | 52805396 | 52805632 | 236 | 35 | 7.10E-06 | 22 | 24 | 2.1 |
| 15 | 62067997 | 62068227 | 230 | 84 | 0.041 | 14 | 15 | 1.4 |
| 15 | 62164653 | 62165056 | 403 | 109 | 5.60E-37 | 26 | 28 | 2.1 |
| 15 | 63600494 | 63601712 | 1218 | 262 | 0.044 | 10 | 11 | 0.3 |
| 15 | 68578366 | 68578783 | 417 | 96 | 6.10E-05 | 11 | 13 | 1.6 |
| 15 | 70853746 | 70853921 | 175 | 56 | 2.70E-07 | 14 | 16 | 2.3 |
| 15 | 72272645 | 72272995 | 350 | 62 | 0.0036 | 16 | 18 | 1.3 |
| 15 | 74366086 | 74366232 | 146 | 39 | 3.70E-06 | 23 | 24 | 1.1 |
| 15 | 77083781 | 77083956 | 175 | 25 | 0.012 | 21 | 23 | 2.0 |
| 15 | 78264718 | 78264909 | 191 | 37 | 0.0018 | 19 | 21 | 1.9 |
| 15 | 79091414 | 79091852 | 438 | 87 | 0.00012 | 11 | 13 | 2.3 |
| 15 | 79431869 | 79432501 | 632 | 165 | 2.70E-25 | 17 | 19 | 2.0 |
| 15 | 82047416 | 82047531 | 115 | 38 | 0.0076 | 17 | 19 | 1.9 |
| 15 | 82445420 | 82445776 | 356 | 31 | 4.90E-08 | 11 | 11 | 0.1 |
| 15 | 82647826 | 82647940 | 114 | 34 | 1.50E-12 | 16 | 18 | 2.1 |
| 15 | 82709764 | 82709867 | 103 | 30 | 0.0051 | 16 | 17 | 1.0 |
| 15 | 82952314 | 82952949 | 635 | 200 | 2.60E-05 | 11 | 11 | 0.6 |
| 15 | 83107250 | 83107333 | 83 | 25 | 0.009 | 19 | 20 | 1.0 |
| 15 | 83107454 | 83107680 | 226 | 76 | 6.50E-20 | 22 | 25 | 2.3 |
| 15 | 83206957 | 83207320 | 363 | 94 | 9.10E-22 | 14 | 16 | 1.8 |
| 15 | 83207320 | 83207422 | 102 | 28 | 0.00033 | 17 | 18 | 0.9 |
| 15 | 83207483 | 83207565 | 82 | 35 | 0.0014 | 17 | 19 | 2.1 |
| 15 | 84080034 | 84080205 | 171 | 27 | 0.0011 | 16 | 18 | 1.9 |
| 15 | 84080255 | 84080469 | 214 | 31 | 3.30E-09 | 16 | 18 | 2.1 |
| 15 | 84633303 | 84634255 | 952 | 227 | 0.0028 | 11 | 11 | 0.5 |
| 15 | 88256980 | 88257185 | 205 | 49 | 1.40E-09 | 22 | 23 | 1.8 |
| 15 | 89399495 | 89399700 | 205 | 39 | 0.00038 | 18 | 20 | 1.8 |
| 15 | 90884255 | 90884375 | 120 | 27 | 2.80E-12 | 30 | 31 | 1.3 |
| 15 | 93088676 | 93088959 | 283 | 46 | 0.038 | 15 | 16 | 1.0 |
| 15 | 93089050 | 93089260 | 210 | 57 | 0.0014 | 14 | 15 | 1.2 |
| 15 | 93089260 | 93089353 | 93 | 32 | 0.0043 | 20 | 21 | 0.4 |
| 15 | 100879267 | 100879517 | 250 | 86 | 0.0025 | 14 | 15 | 1.0 |
| 15 | 100919676 | 100920017 | 341 | 73 | 5.80E-13 | 23 | 25 | 2.1 |
| 15 | 101489213 | 101489860 | 647 | 212 | 2.10E-07 | 10 | 11 | 0.9 |
| 16 | 1153188 | 1153291 | 103 | 45 | 4.40E-06 | 21 | 23 | 1.8 |
| 16 | 1153635 | 1153861 | 226 | 69 | 1.60E-05 | 15 | 18 | 2.3 |
| 16 | 3046883 | 3046983 | 100 | 32 | 3.40E-08 | 27 | 29 | 2.0 |
| 16 | 4328138 | 4328410 | 272 | 29 | 1.60E-05 | 20 | 21 | 0.9 |
| 16 | 9090436 | 9090563 | 127 | 33 | 0.0054 | 20 | 23 | 2.2 |
| 16 | 10182999 | 10183423 | 424 | 98 | 4.90E-30 | 17 | 20 | 2.2 |
| 16 | 12901894 | 12902032 | 138 | 48 | 0.0004 | 14 | 16 | 2.0 |
| 16 | 12902067 | 12902247 | 180 | 50 | 1.20E-05 | 14 | 17 | 2.3 |
| 16 | 21820228 | 21820623 | 395 | 82 | 4.30E-11 | 20 | 21 | 0.6 |
| 16 | 23182412 | 23182638 | 226 | 49 | 0.0031 | 19 | 21 | 2.2 |
| 16 | 23835776 | 23835860 | 84 | 30 | 2.40E-16 | 31 | 34 | 2.2 |
| 16 | 23836003 | 23836226 | 223 | 79 | 2.30E-31 | 22 | 24 | 2.2 |
| 16 | 23836494 | 23836722 | 228 | 44 | 2.10E-17 | 18 | 19 | 0.9 |
| 16 | 25692028 | 25692114 | 86 | 30 | 0.0015 | 20 | 22 | 2.0 |
| 16 | 25692199 | 25692334 | 135 | 43 | 3.40E-11 | 23 | 25 | 1.7 |
| 16 | 25692334 | 25692515 | 181 | 67 | 2.30E-06 | 15 | 16 | 1.5 |
| 16 | 25692607 | 25692735 | 128 | 48 | 4.70E-13 | 17 | 19 | 2.0 |
| 16 | 25692740 | 25692829 | 89 | 26 | 0.045 | 14 | 16 | 1.6 |
| 16 | 28063055 | 28063205 | 150 | 39 | 1.40E-08 | 18 | 19 | 0.8 |
| 16 | 28063387 | 28063548 | 161 | 59 | 3.00E-05 | 15 | 16 | 0.8 |
| 16 | 30417432 | 30418372 | 940 | 287 | 0.015 | 10 | 11 | 0.6 |
| 16 | 31202423 | 31202599 | 176 | 42 | 4.20E-06 | 20 | 21 | 0.8 |
| 16 | 31202603 | 31202761 | 158 | 38 | 0.018 | 17 | 18 | 0.8 |
| 16 | 47143203 | 47143379 | 176 | 44 | 0.015 | 12 | 14 | 1.5 |
| 16 | 54282357 | 54282653 | 296 | 54 | 0.037 | 20 | 21 | 1.8 |
| 16 | 56589226 | 56589446 | 220 | 67 | 5.80E-05 | 12 | 13 | 1.1 |
| 16 | 56625619 | 56625849 | 230 | 37 | 0.012 | 16 | 17 | 0.9 |
| 16 | 57984852 | 57985037 | 185 | 35 | 0.048 | 19 | 20 | 1.5 |
| 16 | 58463484 | 58463871 | 387 | 131 | 8.40E-20 | 15 | 17 | 1.1 |
| 16 | 58463871 | 58464113 | 242 | 72 | 0.0018 | 10 | 11 | 1.0 |
| 16 | 58464380 | 58464691 | 311 | 70 | 0.011 | 13 | 15 | 1.7 |
| 16 | 66844436 | 66844549 | 113 | 28 | 0.00035 | 14 | 16 | 2.1 |
| 16 | 67150854 | 67151363 | 509 | 64 | 0.0076 | 20 | 22 | 1.7 |
| 16 | 67170080 | 67170425 | 345 | 46 | 0.00017 | 27 | 29 | 1.9 |
| 16 | 67170495 | 67170643 | 148 | 22 | 0.039 | 22 | 23 | 0.5 |
| 16 | 67537390 | 67537997 | 607 | 121 | 2.60E-15 | 21 | 22 | 0.5 |
| 16 | 67538239 | 67538563 | 324 | 74 | 3.30E-31 | 29 | 31 | 1.6 |
| 16 | 68084416 | 68084662 | 246 | 20 | 0.0014 | 30 | 31 | 1.2 |
| 16 | 68084662 | 68084999 | 337 | 49 | 7.20E-07 | 20 | 20 | 0.3 |
| 16 | 68448264 | 68448644 | 380 | 86 | 9.00E-07 | 13 | 14 | 1.1 |
| 16 | 79598689 | 79599294 | 605 | 114 | 5.10E-21 | 17 | 18 | 0.9 |
| 16 | 79599337 | 79599588 | 251 | 81 | 2.00E-06 | 14 | 15 | 0.9 |
| 16 | 80932120 | 80932689 | 569 | 110 | 2.90E-08 | 16 | 18 | 1.4 |
| 16 | 85169736 | 85169942 | 206 | 71 | 4.10E-13 | 12 | 13 | 1.2 |
| 16 | 86567139 | 86567288 | 149 | 40 | 5.60E-09 | 14 | 16 | 2.2 |
| 16 | 86567913 | 86568004 | 91 | 22 | 0.00017 | 19 | 21 | 2.3 |
| 16 | 89247814 | 89247936 | 122 | 30 | 4.80E-06 | 14 | 16 | 2.0 |
| 17 | 1179151 | 1179585 | 434 | 78 | 9.30E-06 | 15 | 17 | 1.2 |
| 17 | 2024089 | 2024458 | 369 | 87 | 0.0029 | 11 | 12 | 1.4 |
| 17 | 4584485 | 4584854 | 369 | 109 | 2.60E-06 | 13 | 14 | 1.3 |
| 17 | 5078063 | 5078277 | 214 | 47 | 5.10E-06 | 21 | 24 | 2.3 |
| 17 | 5078411 | 5078596 | 185 | 54 | 5.10E-16 | 23 | 24 | 1.6 |
| 17 | 7705363 | 7705507 | 144 | 34 | 0.04 | 16 | 17 | 1.3 |
| 17 | 8326928 | 8327118 | 190 | 39 | 4.50E-06 | 21 | 23 | 1.6 |
| 17 | 9003187 | 9003613 | 426 | 128 | 2.10E-37 | 21 | 22 | 1.6 |
| 17 | 9003613 | 9003893 | 280 | 44 | 0.0038 | 12 | 14 | 1.7 |
| 17 | 9021626 | 9022101 | 475 | 129 | 0.015 | 11 | 12 | 0.6 |
| 17 | 9022198 | 9022546 | 348 | 95 | 0.00042 | 13 | 15 | 1.5 |
| 17 | 10729687 | 10729937 | 250 | 59 | 0.00023 | 15 | 15 | 0.4 |
| 17 | 16569054 | 16569207 | 153 | 34 | 1.70E-09 | 18 | 19 | 1.2 |
| 17 | 18411580 | 18412036 | 456 | 26 | 0.0096 | 11 | 12 | 1.1 |
| 17 | 19088969 | 19089236 | 267 | 25 | 0.0024 | 14 | 16 | 1.7 |
| 17 | 19867715 | 19868152 | 437 | 135 | 5.20E-10 | 16 | 18 | 2.3 |
| 17 | 20908440 | 20908845 | 405 | 65 | 2.50E-05 | 20 | 20 | 0.9 |
| 17 | 31391234 | 31391436 | 202 | 43 | 3.90E-13 | 22 | 24 | 2.2 |
| 17 | 31391696 | 31391793 | 97 | 40 | 5.10E-05 | 24 | 25 | 1.2 |
| 17 | 33291487 | 33291929 | 442 | 97 | 2.60E-22 | 15 | 17 | 2.1 |
| 17 | 33291985 | 33292085 | 100 | 37 | 0.029 | 15 | 16 | 1.3 |
| 17 | 33292127 | 33292310 | 183 | 64 | 0.0062 | 13 | 14 | 1.2 |
| 17 | 34580261 | 34580463 | 202 | 41 | 0.0011 | 13 | 16 | 2.2 |
| 17 | 35448473 | 35448680 | 207 | 29 | 0.0099 | 13 | 15 | 2.4 |
| 17 | 38453660 | 38453769 | 109 | 39 | 0.047 | 14 | 15 | 1.0 |
| 17 | 39605714 | 39605985 | 271 | 85 | 0.003 | 9 | 11 | 2.1 |
| 17 | 42679379 | 42680088 | 709 | 112 | 0.021 | 7 | 9 | 1.1 |
| 17 | 45432744 | 45432989 | 245 | 58 | 1.80E-12 | 21 | 23 | 1.7 |
| 17 | 47694967 | 47695307 | 340 | 57 | 2.10E-06 | 13 | 15 | 1.8 |
| 17 | 47733591 | 47733831 | 240 | 81 | 3.90E-06 | 13 | 15 | 1.9 |
| 17 | 48597832 | 48598163 | 331 | 77 | 6.80E-14 | 17 | 18 | 1.4 |
| 17 | 49496316 | 49496438 | 122 | 32 | 1.10E-07 | 19 | 22 | 2.4 |
| 17 | 49496607 | 49496933 | 326 | 81 | 9.80E-07 | 13 | 15 | 2.0 |
| 17 | 49497405 | 49497710 | 305 | 67 | 3.60E-07 | 17 | 18 | 1.6 |
| 17 | 49993772 | 49993886 | 114 | 35 | 5.80E-06 | 11 | 11 | 0.5 |
| 17 | 50508436 | 50508757 | 321 | 71 | 1.80E-12 | 17 | 19 | 2.2 |
| 17 | 55265378 | 55265728 | 350 | 68 | 2.60E-18 | 22 | 23 | 0.7 |
| 17 | 56834166 | 56834476 | 310 | 83 | 2.70E-13 | 20 | 21 | 1.8 |
| 17 | 60421460 | 60421647 | 187 | 33 | 3.40E-06 | 24 | 25 | 1.3 |
| 17 | 66964676 | 66964793 | 117 | 48 | 0.00059 | 16 | 18 | 1.9 |
| 17 | 66964845 | 66965247 | 402 | 109 | 0.00028 | 12 | 14 | 1.9 |
| 17 | 68199243 | 68199639 | 396 | 114 | 1.60E-16 | 18 | 20 | 2.4 |
| 17 | 73645176 | 73645470 | 294 | 92 | 0.0005 | 12 | 13 | 0.9 |
| 17 | 74356857 | 74357037 | 180 | 64 | 2.80E-17 | 14 | 15 | 1.7 |
| 17 | 76710619 | 76710775 | 156 | 38 | 0.012 | 8 | 8 | 0.1 |
| 17 | 76868713 | 76869266 | 553 | 124 | 2.30E-16 | 17 | 19 | 2.4 |
| 17 | 77372606 | 77373378 | 772 | 132 | 3.10E-36 | 20 | 21 | 0.9 |
| 17 | 77373399 | 77373701 | 302 | 77 | 1.70E-31 | 25 | 27 | 2.0 |
| 17 | 77373701 | 77374581 | 880 | 137 | 7.40E-96 | 35 | 37 | 2.2 |
| 17 | 79183567 | 79183776 | 209 | 47 | 1.60E-10 | 15 | 17 | 1.7 |
| 17 | 80476134 | 80476330 | 196 | 52 | 0.048 | 13 | 15 | 2.1 |
| 17 | 80476452 | 80476741 | 289 | 101 | 5.80E-07 | 12 | 14 | 2.2 |
| 17 | 80476745 | 80476981 | 236 | 56 | 0.025 | 15 | 17 | 1.9 |
| 17 | 80477324 | 80477489 | 165 | 54 | 0.00045 | 15 | 17 | 2.0 |
| 17 | 82228819 | 82229081 | 262 | 96 | 0.00047 | 12 | 14 | 1.2 |
| 18 | 500226 | 500398 | 172 | 38 | 3.80E-05 | 17 | 18 | 1.5 |
| 18 | 500474 | 500731 | 257 | 97 | 2.30E-16 | 20 | 21 | 1.0 |
| 18 | 2846910 | 2847448 | 538 | 129 | 8.00E-11 | 16 | 18 | 1.1 |
| 18 | 3499084 | 3499255 | 171 | 38 | 0.017 | 18 | 20 | 2.1 |
| 18 | 3499255 | 3499342 | 87 | 29 | 5.60E-13 | 25 | 26 | 1.2 |
| 18 | 5542916 | 5543197 | 281 | 64 | 1.80E-13 | 21 | 22 | 1.5 |
| 18 | 5543611 | 5543775 | 164 | 60 | 1.10E-06 | 15 | 17 | 1.8 |
| 18 | 5543859 | 5543968 | 109 | 28 | 7.50E-06 | 18 | 19 | 1.0 |
| 18 | 5543968 | 5544048 | 80 | 22 | 0.00077 | 23 | 26 | 2.3 |
| 18 | 5630605 | 5630688 | 83 | 22 | 0.00074 | 17 | 18 | 0.9 |
| 18 | 8706625 | 8706753 | 128 | 31 | 3.60E-07 | 27 | 28 | 1.7 |
| 18 | 9707980 | 9708153 | 173 | 42 | 0.00035 | 19 | 21 | 1.6 |
| 18 | 9708283 | 9708729 | 446 | 125 | 2.80E-21 | 19 | 20 | 1.4 |
| 18 | 10454590 | 10454897 | 307 | 108 | 0.0017 | 14 | 15 | 1.1 |
| 18 | 11689774 | 11690038 | 264 | 80 | 0.00032 | 14 | 15 | 0.7 |
| 18 | 12307769 | 12307879 | 110 | 19 | 0.0061 | 27 | 30 | 2.4 |
| 18 | 21242298 | 21242797 | 499 | 129 | 0.00065 | 11 | 14 | 2.2 |
| 18 | 25350257 | 25351012 | 755 | 115 | 3.20E-37 | 23 | 25 | 2.3 |
| 18 | 26548061 | 26548186 | 125 | 40 | 2.30E-13 | 15 | 16 | 0.9 |
| 18 | 32772086 | 32772543 | 457 | 63 | 1.20E-06 | 12 | 14 | 1.9 |
| 18 | 44680206 | 44680517 | 311 | 92 | 1.30E-05 | 10 | 11 | 0.2 |
| 18 | 46028031 | 46028253 | 222 | 37 | 4.90E-08 | 13 | 14 | 0.9 |
| 18 | 46333952 | 46334145 | 193 | 56 | 0.0019 | 14 | 15 | 0.7 |
| 18 | 46334188 | 46334366 | 178 | 56 | 0.00025 | 13 | 14 | 1.4 |
| 18 | 48975375 | 48975656 | 281 | 64 | 0.01 | 12 | 13 | 1.2 |
| 18 | 49560772 | 49561357 | 585 | 92 | 0.037 | 11 | 13 | 1.8 |
| 18 | 59269058 | 59269278 | 220 | 64 | 3.40E-14 | 18 | 20 | 1.4 |
| 18 | 61893132 | 61893503 | 371 | 44 | 6.10E-05 | 23 | 24 | 1.0 |
| 18 | 69400337 | 69400760 | 423 | 85 | 2.90E-15 | 16 | 18 | 1.3 |
| 18 | 69400878 | 69401020 | 142 | 49 | 1.60E-07 | 17 | 18 | 0.8 |
| 18 | 69401020 | 69401203 | 183 | 70 | 5.00E-09 | 14 | 15 | 1.5 |
| 18 | 72543598 | 72543710 | 112 | 42 | 1.40E-19 | 20 | 22 | 1.9 |
| 18 | 77249887 | 77249980 | 93 | 14 | 4.90E-06 | 21 | 23 | 1.7 |
| 18 | 79068937 | 79069018 | 81 | 28 | 0.0026 | 16 | 17 | 1.5 |
| 18 | 79394510 | 79395334 | 824 | 144 | 1.20E-12 | 17 | 18 | 1.0 |
| 18 | 79395745 | 79396087 | 342 | 105 | 6.30E-10 | 15 | 15 | 0.3 |
| 18 | 79396161 | 79396320 | 159 | 41 | 0.00046 | 15 | 15 | 0.5 |
| 18 | 79396505 | 79396780 | 275 | 57 | 0.0013 | 15 | 16 | 1.1 |
| 18 | 79798449 | 79798737 | 288 | 76 | 4.40E-36 | 25 | 27 | 2.2 |
| 19 | 589899 | 589987 | 88 | 37 | 0.023 | 16 | 17 | 0.8 |
| 19 | 590176 | 590377 | 201 | 76 | 0.0022 | 17 | 17 | 0.9 |
| 19 | 590437 | 590702 | 265 | 78 | 0.0046 | 16 | 19 | 2.3 |
| 19 | 917518 | 917779 | 261 | 68 | 4.30E-05 | 12 | 13 | 1.0 |
| 19 | 1000517 | 1000638 | 121 | 50 | 5.90E-06 | 15 | 16 | 1.5 |
| 19 | 1446396 | 1446520 | 124 | 42 | 1.90E-05 | 13 | 15 | 1.9 |
| 19 | 1468968 | 1469994 | 1026 | 295 | 0.0041 | 10 | 12 | 1.1 |
| 19 | 1754526 | 1754714 | 188 | 59 | 1.80E-10 | 17 | 19 | 2.4 |
| 19 | 2290182 | 2290418 | 236 | 76 | 2.60E-17 | 17 | 19 | 1.4 |
| 19 | 3585683 | 3585774 | 91 | 33 | 7.60E-05 | 20 | 22 | 2.0 |
| 19 | 3785654 | 3785828 | 174 | 40 | 1.50E-14 | 22 | 24 | 1.5 |
| 19 | 3785829 | 3785982 | 153 | 46 | 4.50E-17 | 24 | 25 | 1.1 |
| 19 | 3785987 | 3786075 | 88 | 29 | 1.10E-21 | 34 | 36 | 2.2 |
| 19 | 3786075 | 3786202 | 127 | 33 | 7.10E-07 | 17 | 17 | 0.7 |
| 19 | 3786204 | 3786313 | 109 | 22 | 9.00E-05 | 27 | 29 | 1.2 |
| 19 | 7293439 | 7293543 | 104 | 33 | 0.0069 | 11 | 12 | 0.9 |
| 19 | 8209725 | 8210128 | 403 | 62 | 5.60E-08 | 20 | 21 | 1.0 |
| 19 | 8609751 | 8610776 | 1025 | 132 | 1.10E-05 | 13 | 15 | 2.3 |
| 19 | 9786098 | 9786216 | 118 | 30 | 0.0019 | 12 | 12 | 0.2 |
| 19 | 10419311 | 10419392 | 81 | 26 | 0.0017 | 17 | 19 | 1.6 |
| 19 | 11849083 | 11849165 | 82 | 19 | 1.40E-05 | 37 | 38 | 1.4 |
| 19 | 11888185 | 11888266 | 81 | 21 | 0.0083 | 19 | 19 | 0.5 |
| 19 | 12064924 | 12065039 | 115 | 28 | 3.00E-07 | 15 | 16 | 0.8 |
| 19 | 12092506 | 12092621 | 115 | 23 | 0.0052 | 11 | 13 | 1.7 |
| 19 | 12156287 | 12156463 | 176 | 40 | 2.40E-06 | 12 | 13 | 1.4 |
| 19 | 12640121 | 12640280 | 159 | 31 | 0.005 | 20 | 21 | 1.2 |
| 19 | 12885451 | 12885906 | 455 | 95 | 6.20E-12 | 14 | 15 | 1.2 |
| 19 | 14979463 | 14979569 | 106 | 41 | 6.50E-06 | 15 | 18 | 2.4 |
| 19 | 15223765 | 15223849 | 84 | 21 | 0.006 | 26 | 27 | 1.5 |
| 19 | 17448108 | 17448483 | 375 | 94 | 0.04 | 13 | 15 | 2.2 |
| 19 | 18895824 | 18896016 | 192 | 64 | 5.60E-05 | 12 | 13 | 1.2 |
| 19 | 19627724 | 19628198 | 474 | 90 | 3.00E-24 | 25 | 27 | 1.9 |
| 19 | 19628284 | 19628630 | 346 | 82 | 2.50E-10 | 18 | 20 | 1.6 |
| 19 | 21851751 | 21852223 | 472 | 64 | 3.70E-12 | 13 | 14 | 1.2 |
| 19 | 29525314 | 29525423 | 109 | 23 | 3.80E-05 | 12 | 14 | 1.3 |
| 19 | 29526982 | 29527145 | 163 | 28 | 2.30E-14 | 21 | 23 | 1.9 |
| 19 | 29528696 | 29528887 | 191 | 48 | 2.10E-20 | 22 | 24 | 1.3 |
| 19 | 30228714 | 30228843 | 129 | 44 | 4.30E-06 | 12 | 13 | 1.1 |
| 19 | 31348656 | 31348997 | 341 | 69 | 7.70E-49 | 31 | 32 | 1.8 |
| 19 | 31349000 | 31349256 | 256 | 67 | 3.30E-31 | 22 | 22 | 0.6 |
| 19 | 31350815 | 31350932 | 117 | 37 | 0.0022 | 14 | 15 | 0.6 |
| 19 | 34481934 | 34482023 | 89 | 28 | 0.02 | 23 | 24 | 1.4 |
| 19 | 36418467 | 36418659 | 192 | 44 | 5.10E-07 | 16 | 17 | 1.7 |
| 19 | 36418695 | 36418885 | 190 | 46 | 2.80E-08 | 16 | 18 | 1.2 |
| 19 | 36604912 | 36605084 | 172 | 17 | 0.029 | 20 | 22 | 2.2 |
| 19 | 36916207 | 36916347 | 140 | 22 | 0.013 | 16 | 16 | 0.4 |
| 19 | 36916348 | 36916487 | 139 | 32 | 4.70E-11 | 19 | 19 | 0.7 |
| 19 | 38264370 | 38264816 | 446 | 111 | 9.40E-30 | 23 | 25 | 2.4 |
| 19 | 39515059 | 39515197 | 138 | 29 | 0.0038 | 18 | 19 | 1.1 |
| 19 | 42324357 | 42324479 | 122 | 34 | 0.0044 | 18 | 19 | 1.6 |
| 19 | 42401779 | 42402070 | 291 | 76 | 0.00041 | 17 | 17 | 0.5 |
| 19 | 44401557 | 44401681 | 124 | 24 | 0.018 | 13 | 15 | 2.2 |
| 19 | 44753355 | 44754470 | 1115 | 79 | 3.90E-07 | 14 | 14 | 0.7 |
| 19 | 45091506 | 45091914 | 408 | 73 | 0.00096 | 6 | 8 | 2.1 |
| 19 | 47239670 | 47239890 | 220 | 56 | 4.80E-14 | 21 | 23 | 1.3 |
| 19 | 48752490 | 48752833 | 343 | 71 | 6.00E-12 | 20 | 22 | 2.3 |
| 19 | 50050625 | 50051253 | 628 | 89 | 6.20E-47 | 29 | 31 | 2.3 |
| 19 | 50328476 | 50328899 | 423 | 124 | 0.00072 | 13 | 15 | 1.8 |
| 19 | 52336226 | 52336419 | 193 | 35 | 1.50E-08 | 17 | 19 | 2.1 |
| 19 | 52336485 | 52336900 | 415 | 53 | 1.50E-10 | 18 | 20 | 2.4 |
| 19 | 53467198 | 53467365 | 167 | 23 | 5.80E-06 | 27 | 29 | 1.7 |
| 19 | 53979742 | 53980087 | 345 | 47 | 8.70E-09 | 19 | 21 | 2.2 |
| 19 | 53982698 | 53982821 | 123 | 41 | 6.20E-05 | 22 | 24 | 2.1 |
| 19 | 56393483 | 56393577 | 94 | 26 | 8.40E-08 | 20 | 22 | 1.1 |
| 19 | 56508101 | 56508589 | 488 | 27 | 0.00011 | 13 | 14 | 1.9 |
| 19 | 56538590 | 56538709 | 119 | 27 | 0.0069 | 12 | 14 | 2.2 |
| 19 | 56567509 | 56567659 | 150 | 32 | 8.80E-05 | 10 | 10 | 0.4 |
| 19 | 57947708 | 57947812 | 104 | 25 | 2.90E-08 | 21 | 22 | 1.1 |
| 19 | 58002184 | 58002428 | 244 | 42 | 1.70E-06 | 12 | 12 | 0.8 |
| 19 | 58440067 | 58440492 | 425 | 61 | 1.40E-38 | 23 | 24 | 1.6 |
| 19 | 58538412 | 58538685 | 273 | 57 | 2.40E-06 | 15 | 16 | 0.9 |
| 19 | 58562324 | 58563350 | 1026 | 232 | 6.50E-06 | 11 | 13 | 1.8 |
| 20 | 1803001 | 1803310 | 309 | 41 | 0.00059 | 18 | 20 | 2.3 |
| 20 | 1803310 | 1803395 | 85 | 23 | 3.00E-07 | 26 | 27 | 0.7 |
| 20 | 1803466 | 1803631 | 165 | 50 | 5.40E-16 | 21 | 23 | 1.8 |
| 20 | 2692261 | 2692633 | 372 | 108 | 1.00E-07 | 17 | 19 | 2.4 |
| 20 | 4822664 | 4823070 | 406 | 68 | 1.80E-16 | 21 | 23 | 1.7 |
| 20 | 8132322 | 8132569 | 247 | 68 | 0.00075 | 8 | 9 | 0.7 |
| 20 | 18056237 | 18056716 | 479 | 107 | 3.10E-19 | 21 | 23 | 1.5 |
| 20 | 19212640 | 19212898 | 258 | 90 | 5.00E-09 | 17 | 19 | 2.0 |
| 20 | 19758675 | 19759222 | 547 | 77 | 7.50E-09 | 22 | 24 | 1.7 |
| 20 | 20368014 | 20368418 | 404 | 122 | 2.90E-12 | 17 | 18 | 1.6 |
| 20 | 20368421 | 20368885 | 464 | 157 | 3.70E-15 | 14 | 15 | 1.6 |
| 20 | 20369094 | 20369415 | 321 | 98 | 4.30E-07 | 17 | 18 | 1.3 |
| 20 | 21510576 | 21510832 | 256 | 49 | 2.90E-20 | 29 | 31 | 2.4 |
| 20 | 23049168 | 23049674 | 506 | 115 | 7.50E-23 | 18 | 20 | 2.4 |
| 20 | 24469280 | 24469433 | 153 | 44 | 0.00051 | 16 | 18 | 2.2 |
| 20 | 25585418 | 25585624 | 206 | 63 | 0.012 | 13 | 13 | 0.7 |
| 20 | 31587579 | 31588124 | 545 | 117 | 0.0081 | 11 | 12 | 0.7 |
| 20 | 32482406 | 32482854 | 448 | 74 | 1.60E-05 | 20 | 22 | 1.7 |
| 20 | 32583097 | 32583214 | 117 | 40 | 4.40E-07 | 21 | 22 | 1.2 |
| 20 | 34709105 | 34709620 | 515 | 86 | 0.01 | 14 | 14 | 0.3 |
| 20 | 38260517 | 38260854 | 337 | 72 | 0.0018 | 13 | 15 | 2.1 |
| 20 | 38729376 | 38729463 | 87 | 19 | 0.00074 | 17 | 19 | 1.9 |
| 20 | 38805677 | 38805904 | 227 | 45 | 2.50E-05 | 20 | 21 | 1.3 |
| 20 | 40688583 | 40689049 | 466 | 110 | 1.20E-05 | 12 | 14 | 1.7 |
| 20 | 41366409 | 41366540 | 131 | 48 | 6.00E-08 | 18 | 19 | 1.1 |
| 20 | 43507729 | 43507820 | 91 | 21 | 8.90E-05 | 14 | 15 | 0.7 |
| 20 | 44810481 | 44810641 | 160 | 58 | 1.70E-05 | 15 | 17 | 1.8 |
| 20 | 46174649 | 46174736 | 87 | 26 | 7.20E-07 | 21 | 23 | 2.3 |
| 20 | 46308369 | 46308608 | 239 | 50 | 9.90E-08 | 14 | 14 | 0.5 |
| 20 | 48827495 | 48827731 | 236 | 51 | 1.20E-15 | 18 | 20 | 1.9 |
| 20 | 48827731 | 48827818 | 87 | 30 | 0.021 | 9 | 10 | 0.6 |
| 20 | 48827818 | 48827968 | 150 | 60 | 9.10E-06 | 12 | 13 | 0.7 |
| 20 | 48828379 | 48828540 | 161 | 42 | 6.40E-06 | 12 | 12 | 0.7 |
| 20 | 49982629 | 49982860 | 231 | 73 | 0.0017 | 11 | 12 | 1.2 |
| 20 | 51022822 | 51022932 | 110 | 37 | 7.50E-05 | 13 | 14 | 1.3 |
| 20 | 54172985 | 54173279 | 294 | 59 | 7.70E-07 | 18 | 21 | 2.4 |
| 20 | 61252229 | 61252321 | 92 | 36 | 2.20E-10 | 25 | 26 | 1.2 |
| 20 | 61252957 | 61253051 | 94 | 28 | 0.00074 | 16 | 18 | 1.6 |
| 20 | 63006668 | 63006802 | 134 | 33 | 3.60E-05 | 19 | 20 | 1.8 |
| 20 | 63553643 | 63554011 | 368 | 57 | 4.40E-05 | 17 | 19 | 1.8 |
| 20 | 63652268 | 63652415 | 147 | 40 | 1.60E-05 | 20 | 22 | 2.4 |
| 20 | 63652469 | 63652583 | 114 | 33 | 3.40E-08 | 25 | 26 | 1.8 |
| 20 | 63652977 | 63653066 | 89 | 31 | 0.00035 | 20 | 22 | 1.9 |
| 20 | 63830354 | 63830523 | 169 | 61 | 1.50E-16 | 17 | 19 | 1.7 |
| 21 | 20998319 | 20998453 | 134 | 38 | 4.50E-06 | 18 | 20 | 1.5 |
| 21 | 26843789 | 26844338 | 549 | 59 | 3.70E-05 | 12 | 14 | 1.1 |
| 21 | 26844338 | 26844451 | 113 | 34 | 0.00022 | 10 | 11 | 0.5 |
| 21 | 26844454 | 26845417 | 963 | 196 | 4.20E-14 | 8 | 10 | 1.6 |
| 21 | 26846248 | 26847116 | 868 | 87 | 1.20E-22 | 19 | 21 | 2.2 |
| 21 | 33019918 | 33020147 | 229 | 48 | 1.70E-06 | 15 | 17 | 2.4 |
| 21 | 33070588 | 33070890 | 302 | 84 | 0.00012 | 11 | 12 | 1.7 |
| 21 | 33071949 | 33072211 | 262 | 54 | 4.60E-12 | 24 | 26 | 2.3 |
| 21 | 34670241 | 34670381 | 140 | 30 | 1.10E-05 | 22 | 23 | 1.4 |
| 21 | 37006682 | 37007005 | 323 | 102 | 0.029 | 12 | 13 | 0.9 |
| 21 | 45018593 | 45018701 | 108 | 36 | 0.0018 | 20 | 22 | 2.4 |
| 21 | 45642913 | 45643045 | 132 | 28 | 0.0043 | 16 | 18 | 2.0 |
| 21 | 45644142 | 45644353 | 211 | 43 | 7.80E-05 | 19 | 20 | 1.3 |
| 21 | 46097798 | 46097902 | 104 | 28 | 3.20E-06 | 22 | 24 | 2.0 |
| 22 | 19149619 | 19149793 | 174 | 51 | 4.50E-12 | 22 | 25 | 2.4 |
| 22 | 19149793 | 19149904 | 111 | 35 | 0.0055 | 16 | 18 | 2.4 |
| 22 | 19718647 | 19718822 | 175 | 41 | 3.00E-09 | 15 | 17 | 2.1 |
| 22 | 19760764 | 19760898 | 134 | 48 | 0.031 | 15 | 17 | 1.5 |
| 22 | 21657771 | 21658107 | 336 | 69 | 4.50E-07 | 19 | 22 | 2.4 |
| 22 | 24424059 | 24424379 | 320 | 82 | 0.00012 | 12 | 13 | 0.3 |
| 22 | 26657191 | 26657327 | 136 | 28 | 0.00012 | 23 | 25 | 2.2 |
| 22 | 28442031 | 28442545 | 514 | 57 | 0.0016 | 12 | 14 | 1.2 |
| 22 | 28442548 | 28443791 | 1243 | 71 | 9.90E-31 | 24 | 26 | 1.3 |
| 22 | 29073869 | 29074222 | 353 | 67 | 4.70E-17 | 27 | 29 | 2.1 |
| 22 | 29315229 | 29315725 | 496 | 157 | 4.10E-07 | 12 | 14 | 1.5 |
| 22 | 31084583 | 31085769 | 1186 | 179 | 3.40E-51 | 21 | 23 | 2.4 |
| 22 | 32175967 | 32176136 | 169 | 42 | 8.00E-06 | 18 | 20 | 2.3 |
| 22 | 37420284 | 37420620 | 336 | 50 | 0.0028 | 14 | 15 | 1.7 |
| 22 | 38455131 | 38455269 | 138 | 56 | 4.80E-05 | 12 | 14 | 1.3 |
| 22 | 38873000 | 38873444 | 444 | 44 | 0.00086 | 17 | 19 | 2.1 |
| 22 | 39457124 | 39457308 | 184 | 64 | 0.0062 | 9 | 10 | 0.9 |
| 22 | 39457843 | 39458030 | 187 | 56 | 0.048 | 14 | 15 | 1.7 |
| 22 | 41910069 | 41911189 | 1120 | 257 | 3.30E-37 | 20 | 22 | 1.6 |
| 22 | 41914841 | 41915193 | 352 | 108 | 2.90E-09 | 13 | 13 | 0.9 |
| 22 | 41976801 | 41976948 | 147 | 62 | 6.40E-06 | 9 | 9 | 0.3 |
| 22 | 42074329 | 42074584 | 255 | 48 | 1.50E-07 | 22 | 23 | 1.8 |
| 22 | 42283738 | 42283877 | 139 | 35 | 0.0029 | 21 | 22 | 0.7 |
| 22 | 42720618 | 42720885 | 267 | 75 | 0.013 | 11 | 12 | 0.4 |
| 22 | 44752756 | 44752918 | 162 | 26 | 0.0074 | 15 | 16 | 0.9 |
| 22 | 45009149 | 45009408 | 259 | 39 | 0.004 | 18 | 19 | 1.3 |
| 22 | 45009869 | 45010010 | 141 | 44 | 4.00E-05 | 18 | 20 | 1.5 |
| 22 | 45010090 | 45010243 | 153 | 42 | 0.00053 | 18 | 19 | 1.1 |
| 22 | 46536874 | 46537596 | 722 | 236 | 4.80E-18 | 15 | 17 | 2.0 |
| 22 | 46537687 | 46537794 | 107 | 28 | 0.012 | 19 | 19 | 0.6 |
| 22 | 48489864 | 48490109 | 245 | 69 | 0.024 | 10 | 12 | 1.6 |
| 22 | 48575090 | 48575392 | 302 | 72 | 2.80E-08 | 13 | 15 | 1.6 |
| 22 | 48575461 | 48575568 | 107 | 44 | 0.0017 | 15 | 15 | 0.8 |
| 22 | 48575568 | 48575886 | 318 | 116 | 6.40E-20 | 17 | 20 | 2.3 |
| 22 | 48576668 | 48576982 | 314 | 94 | 2.00E-11 | 13 | 16 | 2.4 |
| 22 | 49670465 | 49670573 | 108 | 45 | 1.30E-10 | 20 | 21 | 1.0 |
| 22 | 49670648 | 49670733 | 85 | 22 | 0.03 | 16 | 17 | 0.7 |
| KI270712.1 | 4383 | 4502 | 119 | 21 | 0.0019 | 12 | 13 | 1.7 |
